# Interventions for mental health and substance use in brain injury populations: systematic evidence map and review with interactive data visualization

**DOI:** 10.3389/fdgth.2026.1748621

**Published:** 2026-10-12

**Authors:** Cole J. Kennedy, Yichun Zhao, Simon Kurkimaki, Nicholas Gavas, Emily L. Spargo, Bernard Dupriez-Mitchell, Devon Rees, Zahra Premji, Erica Woodin, Julia Schmidt, Janelle Breese Biagioni, Mauricio A. Garcia-Barrera

**Affiliations:** 1 Department of Psychology, University of Victoria, Victoria, BC, Canada; 2 Institute on Aging & Lifelong Health, University of Victoria, Victoria, BC, Canada; 3 Canadian Institute for Substance Use Research, University of Victoria, Victoria, BC, Canada; 4 Department of Computer Science, University of Victoria, Victoria, BC, Canada; 5 Department of Psychology, University of Utah, Salt Lake City, UT, United States; 6 Libraries, University of Victoria, Victoria, BC, Canada; 7 Department of Occupational Science and Occupational Therapy, Faculty of Medicine, University of British Columbia, Vancouver, BC, Canada; 8 Rehabilitation Research Program, Centre for Aging SMART, Vancouver Coastal Health Research Institute, Vancouver, BC, Canada; 9 CGB Centre for Traumatic Life Losses, Victoria, BC, Canada; 10 British Columbia Brain Injury Association, Victoria, BC, Canada

**Keywords:** acquired brain injury, mental health, neurology, psychiatry, stroke, substance use, systematic evidence mapping, traumatic brain injury

## Abstract

**Background:**

Brain injury, mental health and substance use disorders are leading causes of death and disability worldwide. Despite high rates of comorbidity, it remains unclear how to best support individuals with concurrent brain injury and mental health or substance use problems. The objectives of this study were to: 1) conduct a systematic evidence mapping review on interventions for mental health and substance use in brain injury populations, and 2) develop an interactive user interface to visualize the findings.

**Methods:**

Systematic evidence mapping methodology was used to identify and organize relevant research. PsycINFO, SCOPUS, MEDLINE, the Cochrane Library (CENTRAL and CDSR), Web of Science Core Collection and CINAHL were searched. The AMSTAR-2 was used to evaluate methodological quality and all outcomes are reported according to PRISMA guidelines. Through an iterative prototyping design, the evidence map user interface was developed.

**Results:**

Of the 76,199 records identified, 628 studies involving 815,295 participants with brain injury were included. Records were comprised of 381 impact evaluations, 161 systematic reviews and/or meta-analyses, 39 trial registry records, 43 study protocols and 4 clinical practice guidelines. Most AMSTAR-2 ratings fell within the critically low or low quality range. Cerebrovascular injuries, pharmacological interventions and studies examining depression were among the most researched topics. The development, features and applications of the interactive evidence map are described.

**Conclusions:**

Several patterns across the evidence base emerged and evidence gaps were identified, informing priorities for future research. These findings can be used by clinicians to inform treatment planning and by researchers to guide priority areas for future investigations.

**Systematic Review Registration:**

https://doi.org/10.17605/OSF.IO/P64MT

## 1 Introduction

Acquired brain injury (ABI) is a leading cause of death and disability worldwide, affecting well over 80 million people each year and costing the global economy upwards of 900 billion (US) dollars annually (1–4). Approximately 60% of individuals with ABI are diagnosed with a mental health/substance use (MHSU) disorder (5–7), the treatment of which presents many complex challenges. Depression, anxiety, post-traumatic stress disorder, alcohol use disorder and other commonly occurring MHSU problems after ABI substantially impair rehabilitation and recovery (6–9). While a growing body of research indicates that MHSU disorders are both a risk factor for and a frequent result of ABI (5, 7, 10), health comorbidities, regardless of when they arise, are linked to reduced quality of life, higher healthcare costs and shortened life expectancy following ABI (11, 12). Despite high rates of comorbidity and a breadth of services dedicated to each in isolation, MHSU conditions and ABI have historically been treated as separate entities (13).

It is principally understood that individuals with concurrent ABI and MHSU have distinct treatment needs (7, 14–16). Post-injury neurocognitive deficits in emotional regulation, self-awareness, communication, memory, attention and executive functioning are known to reduce the efficacy of frontline MHSU interventions, both pharmacological and behavioural (6, 16, 17). Despite this, problems with MHSU are generally treated using stand-alone and additive interventions (13, 15). Numerous studies have explored MHSU interventions with ABI-specific adaptations, including common treatments such as psychotherapy and pharmacotherapy regimens (18), more specialized acute interventions, such as transcranial direct current stimulation (19) and hyperbaric oxygen therapy (20), or even the application of traditional medicine, such as acupuncture and herbal remedies (21, 22). While diverse in methodological approach and topical coverage, the current evidence base is incredibly heterogeneous in its findings, generalizability and applicability to frontline care.

There are no known comprehensive systematic reviews of interventions for treating MHSU problems in the ABI population. Those that do exist primarily focus on specific sub-types of ABI, such as traumatic brain injury (TBI) (13) or cerebrovascular incidents (23–26), or target discrete categories of MHSU disorders, such as mood disorders (23–27), trauma and stress-related disorders (28), or other specified behavioral disturbances (29). No systematic review has examined interventions of diverse modalities that target broad MHSU disorders across multiple types of ABI. One possible explanation for the lack of comprehensive systematic reviews is the sheer breadth and depth of ABI and MHSU as scientific phenomena, complicated by the multidisciplinarity of personnel involved in their research and clinical practice. Indeed, the range of intervention types and the vastly divergent taxonomy of ABI and MHSU render it difficult, if not impossible, to conduct a traditional systematic review without narrowing scope.

Evidence mapping offers a unique, modern solution to this problem. Systematic Evidence Maps (SEMs) are a contemporary offshoot of evidence synthesis that provide a broad and comprehensive overview of an evidence base (30). In contrast to other new-age methods such as rapid or scoping reviews, SEMs show what evidence *exists*, not what it says (31). The most fundamental aspects of SEMs are a systematic literature search, narrative synthesis and data visualization output, often a visual figure or graph (30, 32). The narrative synthesis provides a descriptive overview of the evidence base by summarizing the populations, methods, interventions, outcomes and evidence gaps within a topic area, while the data visualization component enhances the usability and accessibility of these findings (32). Regarding the latter, there has been an increasing emphasis on creating more interactive data visualizations that serve as practical resources for stakeholders (e.g., researchers, policy makers, funding agencies, clinicians, etc.) rather than static figures within academic publications (30, 31, 33). Notably, TBI (34–36) and mental health (37–39) are two previously mapped subjects, yet no SEM has targeted these topics together.

The current study describes an initiative called Database of Evidence Concerning Interventions Supporting the Intersections Of Neurotrauma–Mental health & Addictions Problems (DECISION–MAP), a collaborative research project which aims to systematically map evidence on MHSU interventions for people with ABI. We aimed to address the broad question, what evidence exists on different types of interventions for treating MHSU problems in people with ABI? Equally as important, we sought to identify evidence gaps, that is, what evidence doesn't exist? In doing so, we wanted to understand to what degree the current evidence base reflects interventions and outcomes of interest to the broad community of stakeholders. Central to this project was the development of an interactive and open-source SEM user interface. Building on foundations of data visualization and recommendations from experts in the field (30–33), our goal was to create a SEM platform that allows knowledge users to engage with the evidence, enhancing their comprehension and supporting their evidence-based decision-making processes (40).

With these overarching goals and research questions in mind, the specific objectives of the current study were to: (1) systematically identify and organize evidence on interventions that target MHSU in ABI populations; (2) characterize the methodological and thematic landscape of the evidence base; (3) evaluate the methodological quality of included systematic reviews; (4) identify evidence gaps and generate an evidence-informed agenda to guide future research; and (5) develop an interactive SEM interface for knowledge users. Importantly, these objectives stemmed directly from stakeholders calls to action on intersectional ABI and MHSU research (14, 41).

## 2 Methods

The methods for this study followed current standards for conducting SEMs (32) and were based on methodological guidance from Cochrane (29), the Campbell Collaboration (30) and the Collaboration for Environmental Evidence (31). As mentioned earlier, SEMs are a contemporary form of evidence synthesis used to systematically identify and organize existing literature on comparatively (vs. traditional systematic reviews) broad research topics (30), helping to reveal information on characteristics of interest (e.g., interventions, populations), trends and knowledge gaps, which can be used to promote evidence-informed research, clinical and policy decision-making (32). The key components are a systematic literature search, descriptive narrative synthesis and a data visualization output. Unlike many traditional systematic reviews, most SEMs do not directly evaluate study findings (i.e., efficacy) but instead are uniquely distinguished by their purpose of synthesizing an evidence landscape and producing a practical data visualization (32, 33). As the evidence identification and selection process remains the same for both approaches, reporting for this review followed the Preferred Reporting Items for Systematic Reviews and Meta-Analyses (PRISMA) guidelines (42). An *a priori* protocol created according to the Reporting Standards for Systematic Evidence Synthesis (ROSES) (43) for SEMs was registered on Open Science Framework (https://doi.org/10.17605/OSF.IO/P64MT).

Stakeholder consultation is crucial for producing SEMs with practical implications for knowledge end-users (31, 32, 34, 36). Therefore, our objectives, scope, coverage, framework, design and reporting involved numerous rounds of stakeholder consultation. An expert group (*n* = 11) comprised of researchers, clinical psychologists, occupational therapists, people with lived experience of ABI and MHSU, family members of people affected by ABI and MHSU, service providers and other key stakeholders oversaw the development and execution of the project. Consultation with other stakeholders outside the advisory committee was also sought for several aspects of the project.

### 2.1 Literature search

Seven electronic databases were systematically searched: PsycINFO (EBSCOhost), SCOPUS (Elsevier), MEDLINE (Ovid), Cochrane Databases of Systematic Reviews (Ovid), Cochrane Central Register of Controlled Trials (Ovid), Web of Science Core Collection (Clarivate), and CINAHL (EbscoHost). Database searches were executed on October 16, 2024. Relevant keywords and subject headings were mined from seed articles and used to develop the search strategy. As a result of this process, over 100 subject headings and 150 search terms were compiled into a comprehensive search strategy that was applied and translated across databases to describe the following broad search concepts: 1) ABI, 2) MHSU and 3) Interventions. Expert consultation with multi-disciplinary researchers and a librarian (ZP) with expertise in health-related evidence synthesis led to the identification of the chosen information sources and formulation of our comprehensive search strategy. The full search strategy can be found in Supplementary Table S1.

### 2.2 Eligibility criteria

Research records that evaluated interventions for MHSU problems (i.e., Intervention) amongst people with ABI (i.e., Population) that reported on MHSU-related outcomes (i.e., Outcomes) were sought. Specifically, current (i.e., published in the last decade) retrospective and prospective peer-reviewed impact evaluations, clinical practice guidelines, systematic reviews and meta-analyses that reported primary or secondary research findings on interventions involving participants with any and all types of non-progressive ABI were eligible. For our purposes, reviews were considered systematic reviews if they (1) met the Cochrane Collaboration's definition of systematic reviews (44) and (2) explicitly referred to the review as systematic anywhere in the title or main text. Quantitative, qualitative and mixed-method impact evaluations of any design (randomized and non-randomized controlled trials, experimental and quasi-experimental designs, non-experimental designs) with or without a comparator (i.e., Comparison) were eligible for inclusion. Pilot and feasibility studies were included only if they had an impact evaluation component (i.e., reported efficacy data). On-going research, in the form of published study protocols for impact evaluations and clinical trial registry records, were also eligible for inclusion. Given our broad scope, no specific MHSU-related outcomes were sought. Reports that contributed to understanding interventions for persons with ABI and MHSU were considered relevant, so long as one or more of the primary outcomes were MHSU-related and that all other eligibility criteria were met. Table 1 provides an overview of these desired characteristics based on the PICO (Population, Intervention, Comparison, Outcome) format, and Supplementary Table S2 provides a summary of our inclusion and exclusion criteria.

**Table 1:** DECISION-MAP PICO elements.

PICO Components	Delimitation for DECISION-MAP
**P**opulation	People with non-progressive ABI
**I**ntervention	Interventions targeting MHSU-related symptoms
**C**omparison	Any or no comparator
**O**utcome	MHSU-related outcomes

Acquired brain injury (ABI), mental health/substance use (MHSU).

Non-English language records, theses and dissertations, grey literature (e.g., government reports, letters, editorials), animal studies, studies of non-ABI participants or with progressive ABI (e.g., cerebral neoplasms, infections), clinical case studies or series where *n* < 10, published secondary research study protocols, non-impact evaluations, including articles that were narrative, commentaries, or describe a theoretical framework or rationale for interventions, and studies whose primary outcome(s) were not MHSU-related were excluded. Assuming all other inclusion criteria were met, conditional rules were created and followed related to these exclusion criteria: (1) samples of patient-caregiver dyads were eligible for inclusion so long as (i) the intervention was focused on patients and (ii) ≥ 50% of the sample was comprised of patients with ABI; (2) studies involving patients with a range of neurological diagnoses sampled from neurology wards, intensive care units or other similar hospital-based settings were eligible for inclusion if ≥ 90% of the sample was composed of patients with ABI. These exceptions were made to address the complexity of capturing a broad spectrum of study types and designs without excluding potentially informative records. The publication date criterion was set for two key reasons: (1) ABI and MHSU are highly researched topics with hundreds of thousands of published articles and it was not feasible to evaluate all records to date without restricting our comprehensive coverage of conditions, publication types and outcomes; (2) articles published prior to 2014 can be considered outdated relative to the rate of scientific advancement (45) and our objective was to capture “current”, state-of-the-art evidence. Altogether, these inclusion and exclusion criteria were selected as the best fit with our SEM objectives.

### 2.3 Data selection

Identified records were exported from databases and uploaded to the web-based systematic review management software Covidence (46). Given the size of the reviewer team, a training manual was first developed. Then, training and pilot-testing using a portion (200 records each pilot) of the search yield was conducted prior to both stages of article selection. Formal screening did not begin until a minimum of 80% agreement rate was reached on pilot tests. Once achieved, the full search yield was uploaded to Covidence and duplicate records were automatically removed. Following extensive training and preparation of resources and materials, six reviewers independently applied eligibility criteria and selected records for inclusion at the title and abstract stage. To ensure accuracy, a random proportion (10%) of excluded records was evaluated by the team informationist (CJK). Full-text screening was performed independently by two reviewers for all records and all disagreements were resolved by the team informationist. Further discussion and consultation were used to solve disagreements when required. Inter-rater reliability was calculated using Cohens kappa (*κ*). Published study protocols or clinical trial registries for included full text records were identified and removed. To ensure transparency, all decisions were recorded in Covidence and are reported in a flow diagram according to PRISMA guidelines (42).

### 2.4 Data extraction

Pre-determined data elements were extracted from all included records (see Supplementary Table S3). Data extraction was completed by six members of the research team and organized using Microsoft Excel. Information from each included record was carefully extracted and entered into the data extraction form by one reviewer then double-checked by a second reviewer. The team informationist resolved any discrepancies by re-examining full texts to ensure accuracy.

### 2.5 Quality assessment

While risk of bias and critical appraisal assessments are considered optional for SEMs (47), we elected to evaluate the systematic reviews included in our study for two key reasons: (1) to understand the methodological strength of the evidence base, (2) to provide knowledge users with an indication of overall confidence to inform their own evaluations of systematic reviews, and their potential usefulness for their own decision-making processes. Therefore, A Measurement Tool to Assess Systematic Reviews 2nd edition (AMSTAR-2) (48) was used to evaluate the methodological quality of included systematic reviews. The AMSTAR-2 is a widely used tool with strong test-retest reliability and content validity for critically appraising systematic reviews involving randomized and/or non-randomized studies of healthcare interventions (48). As the tool is only designed to appraise systematic reviews (with or without meta-analyses), standalone meta-analyses (i.e., without systematic reviews) were not evaluated. Using the web-based AMSTAR-2 checklist, each of the 16 items were evaluated and an overall quality descriptor was generated (“critically low”, “low”, “moderate”, or “high”). Training and pilot-tests were conducted prior to initiating formal AMSTAR-2 assessments. Two reviewers independently performed the quality assessments and disagreements were resolved by the team informationist via revaluation and consensus.

### 2.6 Data synthesis & visualization

The primary outcomes of a SEM consist of a narrative synthesis describing the key characteristics of the evidence base (i.e., populations, interventions, outcomes) and a data visualization output (31). The methodological system used to organize evidence by categories and subcategories within a SEM is referred to as the *map framework* (31, 47), a critical component that is best formulated through consultive processes with stakeholders (31). Using the standard intervention-outcome matrix configuration (31, 47), our framework organizes interventions based on setting and mode of service delivery on the *y*-axis, and adopts the biopsychosocial model (49) to categorize outcomes on the *x*-axis. Each “higher-level” category is further divided into nested, “lower-level” sub-categories. For example, psychological outcome sub-categories are organized by Diagnostic and Statistical Manual Of Mental Disorders, Fifth Edition, Text Revision (DSM-5-TR) taxonomy (50). Our framework was designed iteratively and informed by multiple sources, including review of previous literature, incorporation of interventions and outcomes of interest to stakeholders identified through parallel prioritization studies (41) and numerous rounds of expert consultation with multidisciplinary researchers, clinicians and other key stakeholders. Through these iterative and collaborative processes, we refined the framework to provide an eloquent and parsimonious approach to organizing data within the map.

The development and testing process for our primary data visualization output, the DECISION–MAP user interface, used an iterative prototyping design methodology and was derived based on feedback through on-going consultation with the expert group (*n* = 11) and over fifty others outside the group, including multidisciplinary researchers, service providers, clinicians, policy makers, people with lived experience and other key knowledge users. Rather than formal metrics, we collected qualitative feedback from stakeholders and integrated it accordingly. A web-based format was selected to offer greater flexibility, allowing access through any web browser on personal computers. To align with the map framework, a 2-D matrix (intervention-outcome) approach was used to organize studies within the visualization. This standard configuration promotes user friendliness while allowing for parsimonious organization of studies, with flexibility for additional complexity and refinement through the integration of filters (31, 47). The fundamental key requirement was to provide a visualization which represents the evidence landscape, both where evidence exists and where it does not. Other key requirements, as identified through stakeholder consultation, included but are not limited the ability to differentiate between study types, interventions, outcomes and types of ABI, and to sort evidence based on whether it is freely accessible (i.e., open access) or behind a pay wall (i.e., standard subscription). Through iterative prototyping and piloting, several other requirements were identified and subsequent modifications were made.

The DECISION–MAP user interface was developed using JavaScript and Vue.js. Interactive design elements were prioritized to enhance user engagement and all evidence sources are hyperlinked. The tabular “bubble” map serves as the core component, allowing users to visualize clusters of evidence and identify gaps in the evidence landscape. Included reports (i.e., data) are plotted on the map and represented as “bubbles”, the size of which depending on the number of reports included within each quadrant, with larger bubbles indicating a larger body of evidence, and bubble colour indicating the type of evidence (i.e., impact evaluation, systematic review, on-going research, clinical practice guideline). Filters and controls were implemented to enable users to customize evidence presented within the map to their personal parameters. DECISION–MAP is open-sourced and free to access globally (http://www.decision-map.com).

## 3 Results

Our database searches identified 76,199 potentially eligible records. After removal of duplicates, 48,603 records were screened. Full texts for articles were sought and 2,157 were retrieved and evaluated against inclusion criteria. Of these, 1,529 were excluded. There was fair inter-rater agreement (81%) and reliability (*κ* = 0.54) for the screening process. Figure 1 is a PRISMA flow chart detailing the article selection process.

**Figure 1:**
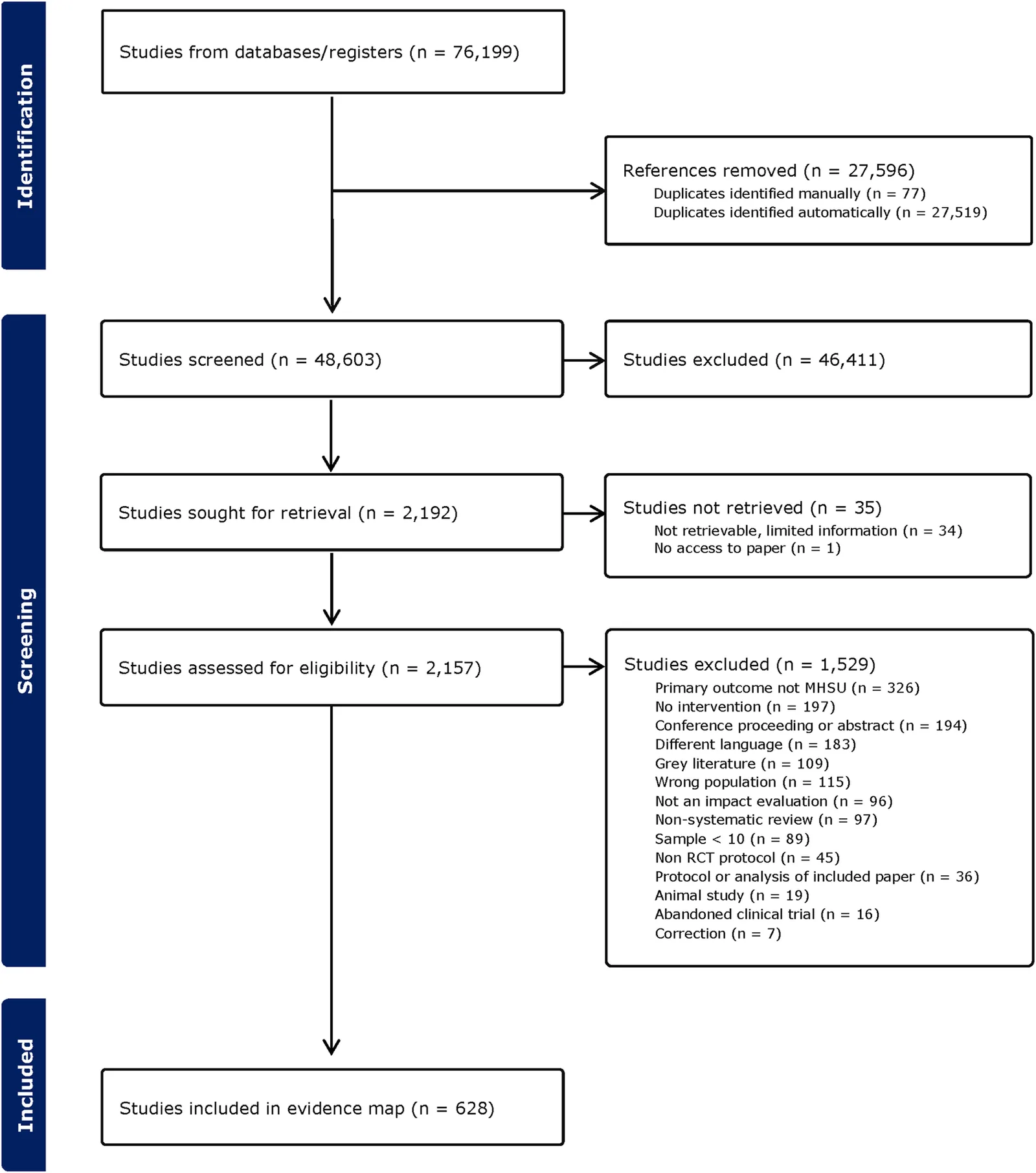
PRISMA flowchart.

The final sample included 628 studies conducted between 2014 and 2024. Temporal publication trends steadily increased, peaking at 101 records in 2022. The majority of records originated from China (*n* = 176), followed by the United States (*n* = 121), United Kingdom (*n* = 33), Canada (*n* = 18), Australia (*n* = 18), South Korea (*n* = 15), Taiwan (*n* = 14), Netherlands (*n* = 11), Spain (*n* = 10) and several others (see Figure 2 and Supplementary Table S4). Systematic reviews and meta-analyses involved primary studies conducted in multiple different countries. Of the included records, 546 were completed studies, comprised of 381 impact evaluations, 136 systematic reviews (with and without meta-analyses), 25 standalone meta-analyses and 4 clinical practice guidelines. There were a total of 82 on-going research records, including 39 clinical trial registries and 43 published study protocols. Regarding accessibility, 59% of all included records were open access (*n* = 372) and the remainder (*n* = 256) were subscription-based publications. Supplementary Table S4 is a summary of all included research records.

**Figure 2:**
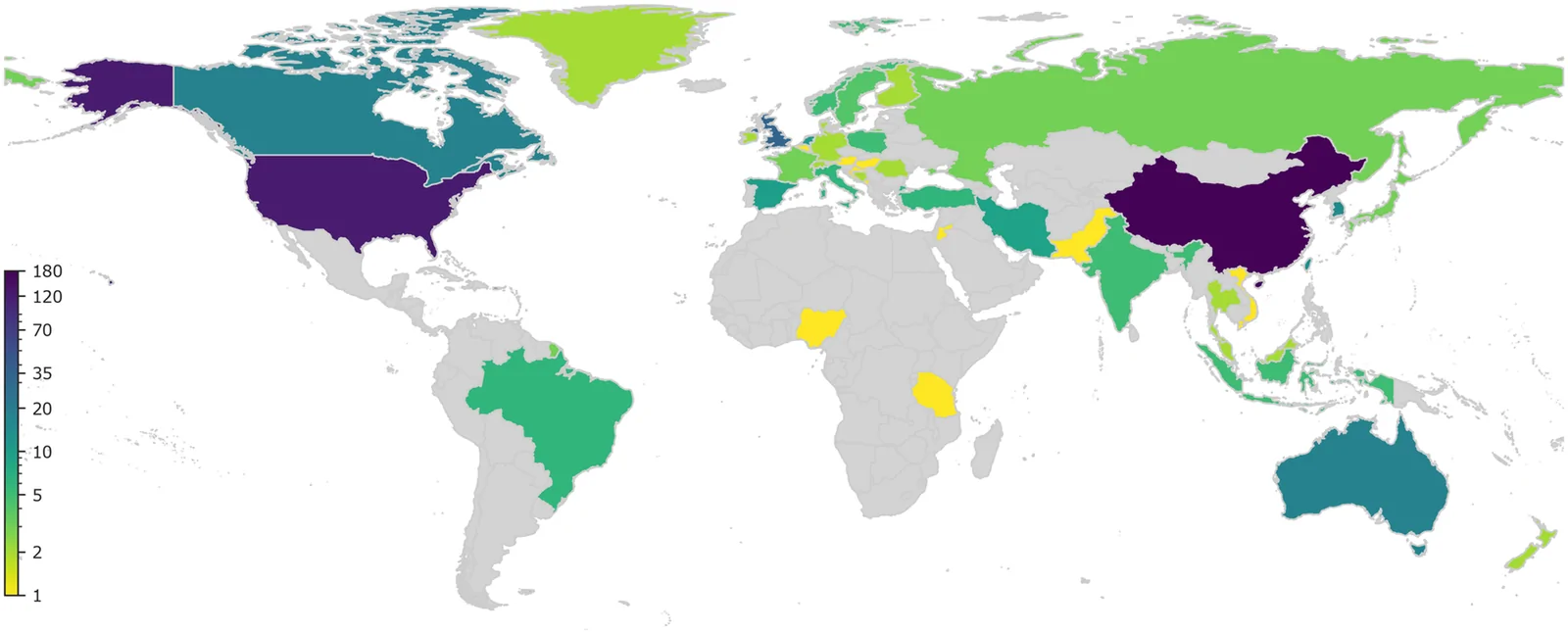
World geographical heat map depicting number of records by country. Scale adjusted to more clearly differentiate lower bounds by colour.

### 3.1 Population characteristics

Our review covers research involving 815,295 people with ABI. The average age of participants was 56 years (*SD* = 11.5 years) and most studies included individuals of both male and female sex. Few (*n* *=* 8) included male subjects only, and one study's sample was comprised exclusively of female sex participants. Relatively few (*n* *=* 86, 14%) articles reported information on participants' race or ethnicity or sexual orientation and gender. Of the articles that reported this level of participant demographic information, there were 5,902 Black, Indigenous, and people of color and 8 transgender or non-binary individuals. Sixty-four studies specifically sampled military populations, involving 56,254 service members or veterans with ABI (7% of the total sample). Regarding sub-types of ABI investigated, ischemic and hemorrhagic stroke was the most commonly investigated (*n* *=* 434), primarily comprised of studies on post-stroke depression (*n* *=* 369), followed by non-specific (i.e., any severity) TBI (*n* *=* 108), mild TBI and/or concussion (*n* *=* 44), moderate TBI (*n* = 2), both moderate and severe TBI (*n* *=* 6) and severe TBI (*n* = 5). Several (*n* = 28) studies involved participants with ABI in general and did not delineate between sub-types. One study focused specifically on participants with anoxic brain injuries (51).

### 3.2 Study designs

Of the 455 primary research studies, majority (*n* = 351, 77%) employed experimental (randomized controlled trial) designs. The second most common study design was lower-tier quasi-experimental designs (*n* = 37), followed by non-experimental designs (*n* = 36) and upper-tier quasi-experimental designs (*n* = 31). Figure 3 depicts the number of publications by study design for impact evaluations. Of the 160 systematic reviews and meta-analyses, 92 were systematic reviews with meta-analyses, 43 were systematic reviews without meta-analyses and 25 were standalone meta-analyses. Eight were Cochrane commissioned reviews. Few studies employed qualitative (*n* = 16) or mixed-methods (*n* = 30) approaches, with the overwhelming majority of evidence being quantitative in nature.

**Figure 3:**
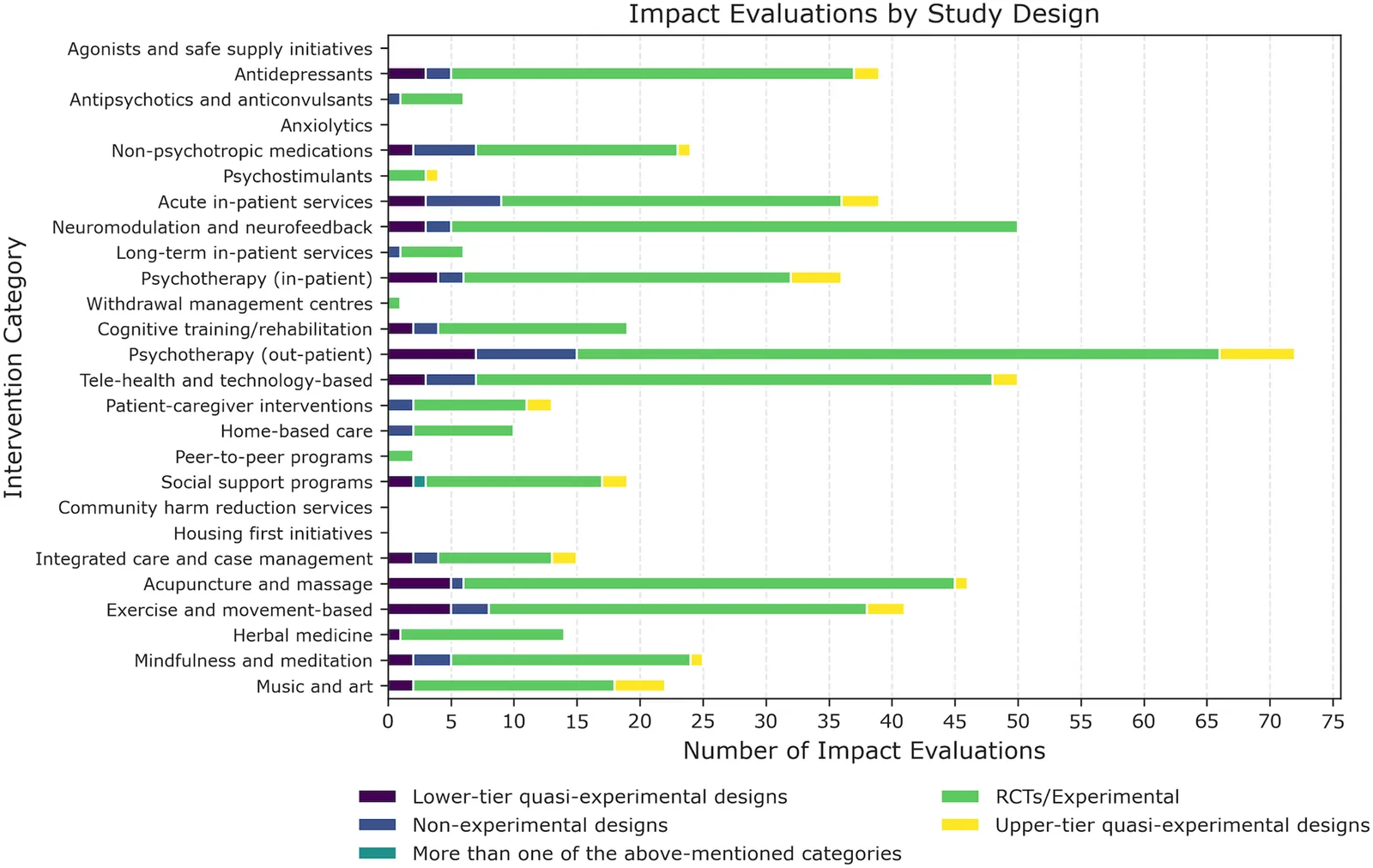
Number of impact evaluations organized by study design.

### 3.3 Assessment of methodological quality

The AMSTAR-2 ratings for included systematic reviews with or without meta-analyses (*n* = 135) were as follows: 54 (40%) were rated as critically low (i.e., more than one critical flaw with or without non-critical weaknesses), 55 (41%) were rated as low (i.e., one critical flaw with or without non-critical weaknesses), 12 (9%) were rated as moderate (i.e., more than one weakness but no critical flaws) and 14 (10%) were rated as high (i.e., no or one non-critical weakness) quality. The agreement rate between appraisers was 81%, indicating good agreement for overall AMSTAR-2 ratings across evaluated articles. Supplementary Table S5 lists the AMSTAR-2 ratings for all included systematic reviews.

### 3.4 Evidence by interventions

Figure 4 is an intervention-outcome heat map depicting the number of records in each quadrant of the matrix. Pharmacological interventions were the most common across, involving 70 primary studies (*n* = 55 randomized controlled trials) and 55 secondary studies. The majority of pharmacological interventions focused on depressive disorders (*n* *=* 97), with antidepressants (*n* = 86) being the predominant class of studied pharmaceuticals. Thirty articles studied the use of non-psychotropic medications, such as N-acetylcysteine (52), pioglitazone (53), and vitamin D (54) or N-Pep-12 (55) supplements. Fewer studies (*n* = 8) explored antipsychotic and anticonvulsant medications, which were primarily used to treat agitation and aggression in TBI patients. Anxiolytics and psychostimulants were the least represented. Many studies involving pharmacological interventions were cross-categorized in more than one intervention category (*n* = 28, 27% of all pharmaceutical interventions), indicating that pharmaceuticals are often examined as adjunctive treatments with other types of interventions; most commonly, out-patient psychotherapy with antidepressants.

**Figure 4:**
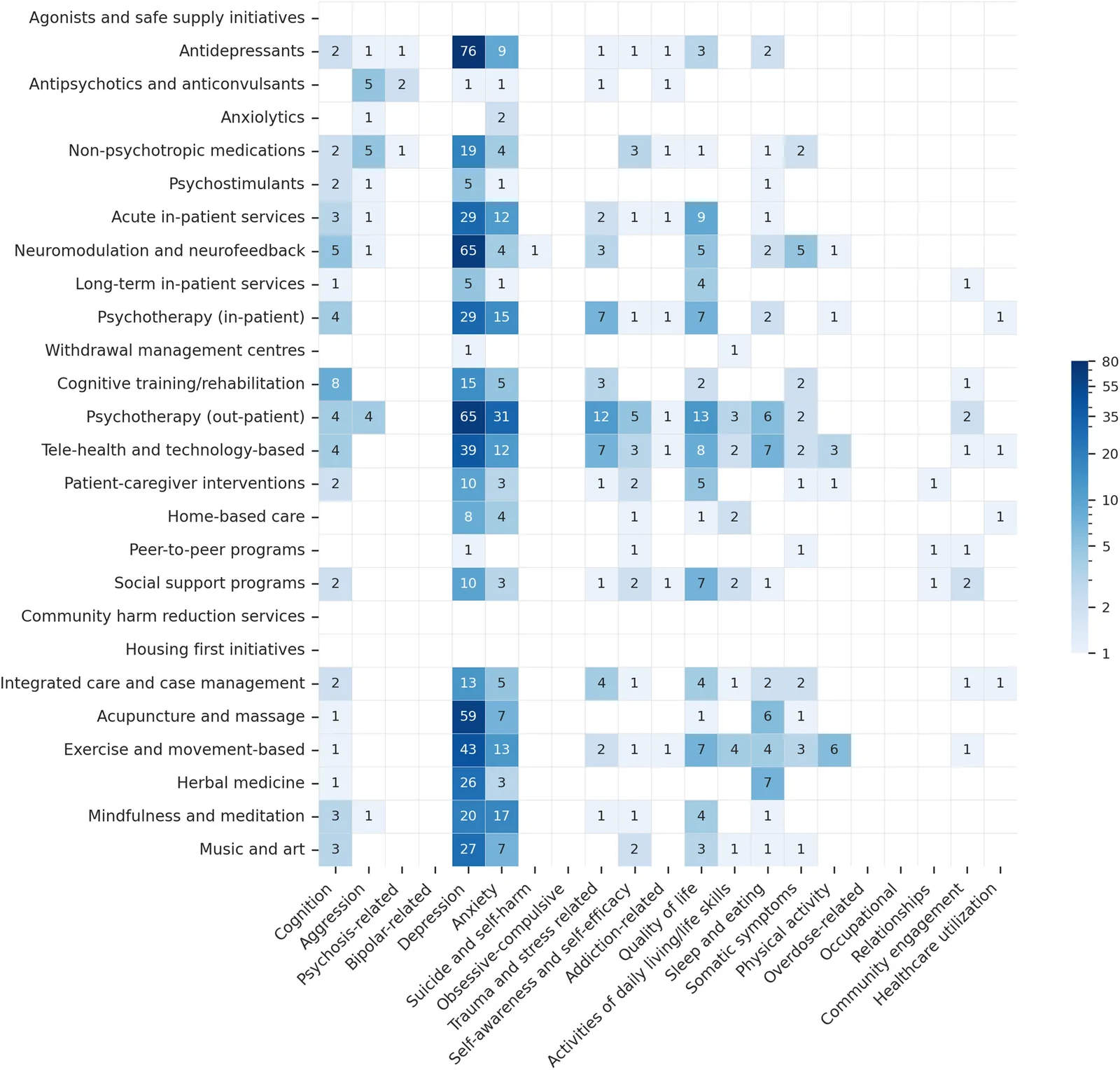
Heat map matrix illustrating number of records in given intervention-outcome quadrants. Darker tiles indicate greater number of records. White tiles indicate evidence gaps.

Studies involving in-patient and out-patient interventions were well represented, with a relatively equal distribution of evidence across the two categories (*n* *=* 160 in-patient, *n* *=* 169 out-patient). Regarding in-patient interventions, 73 studies examined neuromodulation and neurofeedback, primarily for depression (*n* *=* 66), but also for treating anxiety (*n* = 7) and trauma and stress-related (*n* = 6) disorders. Neuromodulation and neurofeedback interventions had a total of 23 systematic reviews and meta-analyses, more so than any other in-patient or out-patient intervention category, coming in second to antidepressants for the greatest number of synthesis research articles in the map overall. Forty-one studies examined the impact of interventions delivered in acute in-patient settings, such as evidence-based and comprehensive nursing regimes (56) or hyperbaric oxygen therapy (57). Psychotherapy was the second highest populated sub-category, with most psychotherapy studies being conducted in out-patient (*n* *=* 101) compared to in-patient (*n* = 42) settings. Sixty in-patient and out-patient psychotherapy research records involved telehealth and technology-based methods, such as remote cognitive behavioural therapy (58) or virtual reality (59) interventions. Only one study involved withdrawal management, specifically an alcohol withdrawal assessment protocol in an in-patient TBI rehabilitation setting (60). Notably, the out-patient intervention category demonstrated significant potential for growth in future findings, as evidenced by it having the highest number of on-going research records (*n* = 26) within our sample.

Community-based interventions were less commonly examined among the included records. Evidence in this category primarily encompassed studies that evaluated patient-caregiver (*n* *=* 17) or home-based care (*n* *=* 11) interventions. Three studies focused on peer-to-peer programs (61–63). Congruent with the rest of the evidence base, most of these studies involved samples of stroke (*n* *=* 35) or people with TBI (*n* *=* 11) and focused on depression or anxiety as outcomes. Studies examining integrated care were sparser. Of the records in this category, 20 reported on integrated care and case management activities, involving social workers, nurses, psychologists, occupational therapists and other professionals delivering multidisciplinary and coordinated healthcare services. No research examining community-based harm reduction services or housing first initiatives was identified, indicating an evidence gap for these types of interventions in the literature.

The alternative approaches category was the third most populated intervention category within the map, containing 136 primary and 59 secondary studies. Fifty-percent of studies within this category investigated the effects of acupuncture (*n* *=* 68) or herbal medicine (*n* *=* 31), including 28 systematic reviews and meta-analyses, primarily for treatment of post-stroke depression. Studies on exercise and movement-based interventions were well represented (*n* *=* 54) and involved a broad range of physical exercise activities, such as aerobic exercise (64), including aquatic aerobic exercise (65), yoga (66), tai chi (67) and surfing (68). Thirty studies investigated interventions using mindfulness and meditation-based approaches, mostly involving stroke (*n* = 22) but also TBI (*n* = 7) samples. Music and art interventions were the least of the studied alternative approaches, with 22 primary and five secondary studies. Notably, alternative approaches housed the second most ongoing research records (*n* = 24) of any broad-level intervention category within the map, suggesting that more researchers are turning their focus to investigating the impacts of less conventional approaches for ameliorating MHSU symptoms in ABI populations.

### 3.5 Evidence by outcomes

Identified study outcomes were organized using the biopsychosocial model into one of three broad categories (i.e., physical, psychological or social), each containing sub-level categories. Of the three, social outcomes were the least represented among the included records (*n* = 14), comprised of nine impact evaluations, three clinical trial registries and two registered study protocols. Within this category, community engagement was most common (*n* = 9), followed by healthcare utilization (*n* = 3) and relationships (*n* = 2). Physical outcomes were the second least represented (*n* = 62) with 48 primary and 14 secondary research records, comprised of 42 impact evaluations, six systematic reviews and meta-analyses, one clinical practice guideline, five published study protocols and four clinical trial registries. Studies evaluating the effects of interventions on sleeping and eating related outcomes were most common (*n* = 34), primarily focusing on fatigue or insomnia, followed by somatic symptoms (*n* = 18) and physical activity (*n* = 10) related outcomes. Overdose-related outcomes emerged as a substantial evidence gap, demonstrated by the lack of records examining overdose-related outcomes across all the included studies.

Psychological outcomes were the most studied among the included records, with 442 primary and 162 secondary research articles, comprised of 368 impact evaluations, 155 systematic reviews and meta-analyses (42 systematic reviews, 25 standalone meta-analyses, 88 systematic reviews with meta-analyses), four clinical practice guidelines, 40 published study protocols and 37 clinical trial registries. Majority evaluated the impact of interventions on depression (*n* = 463) or anxiety (*n* = 128), many of which examined both (*n* = 112) through combined measures such as the Hospital Anxiety and Depression Scale (69). Quality of life was the third most populated outcome category (*n* *=* 74), followed by cognition (*n* *=* 41), trauma and stress-related (*n* *=* 33) and sleep and eating (*n* = 34). In comparison to other psychological outcomes, studies measuring aggression or trauma and stress-related outcomes were more likely to involve people with TBI as participants in their investigations. Eight studies examined addiction-related outcomes, mostly severity or frequency of alcohol use, and were comprised of seven primary research studies and one evidence synthesis report. No research examining obsessive-compulsive or bipolar-related outcomes was identified. Altogether, the psychological outcomes category encompassed 89% of all included records within our sample.

## 4 Discussion

This study evaluated and synthesized the landscape of completed and ongoing research on interventions for MHSU problems amongst individuals with ABI. Importantly, we examined what evidence exists, not treatment efficacy. Central to this initiative was the development of an interactive SEM platform. This section first examines the evidence identification, organization and evaluation aspects of the project, then turns to discussion of the interactive data visualization platform.

Majority of the research originated from China, the United States, United Kingdom, Canada and Australia, with a relatively steady increase in the amount of research being published over time. Impact evaluations testing the effect of interventions, predominately using randomized controlled trial designs, were the most represented study type, followed by systematic reviews and meta-analyses. In terms of broader methodologies employed, most research was quantitative, with relatively few qualitative or mixed-methods studies. Across the evidence base, only four relevant clinical practice guidelines were identified (15, 70–72).

Evidence and gap analyses revealed several patterns, most notably a strong affinity for research examining post-stroke depression. Given that nearly one in three stroke patients will suffer from depression (73), paired with the upwards trend of stroke incidence overall (3), the large body of research on this area is to be expected. The traditionality of examining non-traumatic and traumatic subtypes independently may also influence the predominance of research dedicated to post-stroke depression, as opposed to investigations on depression in the broader ABI population. In terms of treatments studied, pharmacological interventions, particularly anti-depressants (e.g., sertraline), and psychotherapeutic interventions, primarily cognitive behavioural therapy, possessed the most evidence, as did neuromodulation (e.g., repetitive transcranial magnetic stimulation) and alternative approaches (e.g., acupuncture). In comparison, there was a dearth of published research focusing on substance use and addiction overall.

Several evidence gaps were identified. There was little-to-no peer-reviewed research on withdrawal management centres, long-term in-patient services, or peer-to-peer programs as interventions. Although these modalities are well supported as effective treatments for a broad range of MHSU-related complications in general, greater research is needed to examine the transferability of this effectiveness to individuals with neurocognitive and other common post-ABI impairments. Given the diagnostic challenges of disentangling ABI and MHSU symptomatology (6, 13, 16), paired with the known high rates of co-occurrence (7, 8, 10), it is probable that many people with unrecognized ABIs are frequenting these treatment settings already, without their ABI-specific needs addressed. Peer-to-peer programs, in particular, have shown great promise in both ABI and MHSU populations separately, suggesting strong potential for supporting individuals with concurrent presentations (62).

Community harm reduction services, agonists and safe supply initiatives and housing first initiatives were the only three intervention categories for which no peer-reviewed evidence was identified. In Canada, all of these are increasingly commonplace strategies with proven efficacy and endorsements of support by stakeholders (41, 74–77). In regard to the former two categories, previous research indicates that individuals with history of TBI are at higher risk for developing problematic substance use (7, 78, 79) and that they are ten-times more likely to die from accidental drug poisoning than the general public (12). In cases of non-fatal drug poisoning, opioid-induced respiratory depression may lead to cerebral hypoxia, which if not resolved quickly, can leave survivors with mental and physical disability from hypoxic, anoxic and/or ischemic injuries (80, 81). The issues of housing and homelessness are also deeply engrained in the intersections between MHSU and ABI, with meta-analytic evidence suggesting that roughly half of all people who are homeless have experienced a TBI (82). The lack of peer-reviewed literature on harm reduction and housing interventions, in combination with our result of no studies examining overdose-related outcomes, represents a substantial evidence gap. Notably, these areas have been declared as top priorities amongst community-based stakeholders for future research (41, 77).

In regard to the outcomes studied, very few records examined healthcare utilization, psychosis-related or relationship-related outcomes. Healthcare utilization is an important consideration for those with concurrent ABI and MHSU problems (10), especially given that stigma associated with each cluster of conditions has been found to deter patients from seeking healthcare services (83, 84). While research on psychosis-related disorders post-ABI has been scarce in comparison to other more common forms of psychopathology, there is evidence to suggest an increased risk of schizophrenia and bi-polar disorders after ABI (7), including a dose-response relationship of more severe TBIs being associated with greater risk of psychotic disorder onset (85). While researchers are yet to establish causality between ABI and psychosis, the lack of knowledge on treating or preventing psychosis-related symptoms post-ABI serves as a notable gap in the evidence landscape.

Across the over six-hundred records included in this review, only one study examined suicide and self-harm: a clinical trial registry for a randomized controlled trial using intermittent theta burst brain stimulation for treating suicidal ideation and impulsivity in veterans with mTBI (86). This finding is incredibly concerning given that suicide risk is doubled for people with history of TBI (12). The lack of peer-reviewed information on interventions directly targeting suicidality is a significant gap in knowledge that requires immediate attention.

The most generalized evidence gap was the limited research focusing on interventions and related outcomes for substance use and addictions. Substance intoxication is not only a common feature of psychiatric dysfunction following ABI (7, 78, 79), but it is also a leading predictor of accident-related ABI (5, 87). Within our sample, the few records that did include substance use and addiction-related outcomes primarily studied alcohol use disorder in veteran populations. Whether civilian or military, individuals with ABI have higher rates of problematic substance use, extending beyond just alcohol (5–7). Furthermore, there is evidence to suggest that case-specific factors (e.g., age at injury, severity of injury, number of injuries) are linked to self-reported and objective neurocognitive deficits (e.g., processing speed, working memory) as well as severity of substance use (88), highlighting a need for a broad range of intervention modalities that can be applied using a patient-centered, tailored approach. The overall limited amount of peer-reviewed research on how to best support the unique treatment needs of those with concurrent ABI and substance use concerns represents a tremendous gap in the evidence base that must be addressed.

### 4.1 Methodological quality of evidence

Given the volume of systematic reviews within our sample, examination of their methodological quality was an integral component of this study. Methodological quality of systematic reviews as assessed by the AMSTAR-2 varied, with the strong majority of reviews falling within the critically low to low range, indicating a pitfall in the confidence of synthesis reports. Some of the more common items where synthesis studies fell short on were related to the comprehensiveness of search strategies (e.g., number of databases searched), reporting of information from included articles (e.g., sources of funding, detailed participant characteristics) and quantitative analyses (e.g., failing to statistically examine effect of publication bias), when applicable. This finding of low confidence is concerning, given the substantial growth of synthesis reviews as sources of information (89) and our reliance on them for clinical decision-making, including when determining best practices for diagnostic or treatment approaches (90). Our findings reinforce the need for knowledge users to be critical of the evidence base, recognize that not all systematic reviews are created equal, challenge the idea that reviews are “objective” and to consider evaluating methodological quality themselves using well-validated tools, such as the AMSTAR-2 (48, 90). Notably, the identification of critically low methodological quality is an emerging pattern in the broad literature, demonstrating that this is an issue affecting not only the studies on ABI and MHSU, but the field of synthesis research at large (see Supplementary Figure 1) (91). SEMs offer a potential remedy for circumnavigating this issue as some allow knowledge users to explore synthesis evidence by quality, helping to inform their decision-making processes. Nevertheless, researchers should strive to produce the most methodologically sound and rigorous systematic reviews to enhance the quality of the knowledge synthesis evidence base.

### 4.2 Dynamic SEM visualization platform

The DECISION–MAP user interface delivers an interactive and dynamic visualization of the evidence base. The tabular bubble map enables users to visualize clusters of evidence and identify gaps. Each bubble represents the included records within a given quadrant. Bubble size corresponds to the number of records, with larger bubbles indicating a larger body of evidence, and bubble color indicates the type of evidence. When users hover their cursor over a bubble, the quadrant-specific (i.e., Y and X intercept) information is displayed as a tooltip. Within it, studies are hyperlinked to their original full text sources, allowing users to readily engage with the evidence. To promote further accessibility, particularly for users outside of academia who are likely without journal subscriptions (e.g., community-based care providers), the SEM can be restricted to presenting open access (i.e., free) articles only. Filters allows users to sort evidence by geographical coverage, population characteristics, method or study design, personalizing data output so that it is most relevant to users' information needs, which are likely to differ based on role or profession. For example, clinicians may use these controls to filter evidence based on patient attributes, including type of ABI (e.g., mild, moderate or severe TBI, stroke, etc.) or target problem (e.g., sleep, substance use, trauma and stress-related, etc.), to guide individualized treatment planning, whereas funding agencies might apply portfolio-level filters and view evidence gaps to prioritize strategic investments, avoid duplication and commission synthesis studies where uncertainty remains. Furthermore, previous research suggests that interactive data visualizations might be especially preferred by policy makers (92), potentially increasing their impact for effecting systemic change. In addition to research agenda setting, literature reviews, study planning and other key uses, researchers—a prime intended user audience—may also leverage the tool when conducting syntheses of secondary research, such as umbrella reviews or meta-meta analyses, which in many cases are considered “gold standard” for informing clinical care (90).

Our vision was to create a data visualization platform with practical utility to as many stakeholders as possible—not just researchers, clinicians and policy makers. It was through consultation with community-based service providers that workflows and requirements were understood and additional features were created, such as the ability to “tag” studies with comments, or to copy information to users' clipboards. To the best of our knowledge, DECISION–MAP is the first interactive SEM to incorporate these functions. In our case, such innovations would not have been possible without stakeholders' input, further underscoring the value of stakeholder involvement in SEM projects (31, 47).

In addition to stakeholder engagement, DECISION–MAP is further distinguished from other previous SEMs by its visualization approach. Approximately two years ago, an analysis of health-related scoping and evidence-mapping reviews found that most visualizations were static, mainly bar and pie charts (33). Furthermore, only two out of the over 200 studies included in the analysis included interactive data visualization outputs; only one of which hyperlinked references to their sources. While more interactive SEMs have since been published (32, 93), most use pre-existing software's such as EPPI-Mapper or Tableau (31–33), limiting interface functional and structural flexibility. Additionally, these software can come at notable financial cost to researchers. By building an interactive data visualization user interface from the ground up, we were able to employ an interactive prototyping design that could dynamically incorporate user feedback while offering complete customizability to best achieve our objectives.

Continued engagement with stakeholders in the form of knowledge mobilization is crucial to support the uptake of evidence mapping initiatives like DECISION–MAP. Without awareness for these low-barrier knowledge exploration platforms, individuals and organizations are unable to utilize them in their research, clinical or policy-related tasks. Formal implementation studies on how SEMs are adopted, used and sustained in real-world decision-making contexts, while identifying opportunities for improvement, are needed to promote the widespread uptake of SEMs and evaluate their true impact on the health information landscape.

### 4.3 Limitations & future directions

This systematic evidence mapping review had five main limitations. First, our search strategy was limited to records published from 2014 onwards, and therefore, it is possible that informative articles published prior to then may have been missed. However, most of the over one-hundred included systematic reviews captured evidence published prior to 2014, meaning that this information is not entirely absent from our analysis. Second, while our inter-rater agreement and reliability was fair and steps were taken to ensure accuracy (e.g., pilot, training, spot-checking), it is possible that potentially relevant records may been excluded in the screening process. Third, we did not include grey literature, which means we may have excluded potentially informative program reviews, government-funded service evaluations or other (non-peer-reviewed) reports. It is possible that grey literature, which can be more common than peer-reviewed studies in emerging areas, may exist within the evidence gaps we identified, and that the map may be susceptible to publication bias and the favouring of elitist sources of information. Targeted, narrower scope evidence syntheses that include grey literature are required to make definitive claims on the absolute absence of real-world intervention activities in these areas. Fourth, articles published in different languages are not reflected here, as our team is primarily English-speaking and was limited to evaluating English-language publications only. Fifth, as with most evidence syntheses, the time between a systematic search and the final output can create an environment of aging evidence. While we plan to conduct a literature update and are dedicated to maintaining the tool, our comprehensive SEM methodology requires significant resources (i.e., time and personnel) to do so, particularly during the data selection and extraction processes, due to the large evidence base. Recent advances in artificial intelligence and machine learning have shown promise for overcoming this challenge by streamlining screening and data extraction (32) and, most recently, automatically updating interactive evidence map user interfaces (93). We encourage researchers to expand upon these limitations and incorporate innovative approaches to overcome them in their future investigations.

## 5 Conclusion

This paper presents a comprehensive SEM covering a diverse spectrum of interventions for MHSU in ABI populations. This is the first ever review undertaken to capture and describe this information, serving as a significant knowledge source for researchers, practitioners and healthcare policy makers. Such a wide breadth of study types is uncommon, even for SEMs, rendering this work a first in the field of evidence mapping and health-related knowledge synthesis. Development of the interactive DECISION–MAP user interface (http://www.decision-map.com) serves as a valuable tool to researchers, clinicians, funding agencies and policy makers to aid in their primary study and systematic review planning, treatment formulation and evidence-based funding and healthcare policy decisions. Importantly, our findings highlight several knowledge gaps in pressing areas of intersectional ABI and MHSU research. These provide an evidence-based agenda for researchers and funding decision-makers to maximize the impact of future studies and support advancements in designing and delivering effective interventions for these highly prevalent and commonly co-occurring health conditions.

## Data Availability

The original contributions presented in the study are included in the article/Supplementary Material, further inquiries can be directed to the corresponding author/s.
