# Supplementary material for: Interventions for mental health and substance use in brain injury populations: systematic evidence map and review with interactive data visualization
Source: Front Digit Health. 2026 Oct 12;8:1748621. doi: 10.3389/fdgth.2026.1748621 (PMC13696407; doi:10.3389/fdgth.2026.1748621)
Supplement: Supplementary file 1 [file Datasheet1.docx]

Supplementary Material

# 1 Supplementary Figures

## 1.1 Supplementary Figure 1. *Time Series of Systematic Reviews by AMSTAR-2 Ratings.*

# 2 Supplementary Tables

## 2.1 Supplementary Table 1. *Full Search Strategy.*

| **DECISION-MAP Search Equation – MEDLINE**  **(Ovid)** | | | | |
| --- | --- | --- | --- | --- |
| ABI | 1 | Brain Injuries/ | 55671 |  |
|  | 2 | exp brain hemorrhage, traumatic/ or exp brain injuries, diffuse/ or brain injuries, traumatic/ or brain concussion/ or brain contusion/ or brain injury, chronic/ | 24966 | Subject headings, ABI |
|  | 3 | craniocerebral trauma/ or head injuries, closed/ or head injuries, penetrating/ or intracranial hemorrhage, traumatic/ | 28089 |  |
|  | 4 | exp brain ischemia/ or intracranial hemorrhages/ or exp stroke/ or exp hypoxia, brain/ | 232103 |  |
|  | 5 | ("traumatic brain injur*" or tbi or neurotrauma* or "neuro-trauma*").tw,kf. | 54468 | Keyword terms including acronyms, ABI.  The concussion search string from the following paper were adapted and used: Schneider et al. (in press). The Amsterdam 2022 Process: A Summary of the Methodology for the 6th International Consensus Conference on Concussion in Sport. British Journal of Sports Medicine. |
|  | 6 | (head adj2 (injur* or impact* or trauma*)).tw,kf. | 38193 |  |
|  | 7 | (brain adj2 (injur* or impact* or trauma*)).tw,kf. | 92285 |  |
|  | 8 | (concussion* or concussed or concussive or "mild traumatic brain injur*" or mtbi).tw,kf. | 16424 |  |
|  | 9 | (craniocerebral adj2 (trauma* or injur*)).tw,kf. | 3339 |  |
|  | 10 | ("acquired brain injur*" or abi or "non-traumatic brain injur*" or "non traumatic brain injur*" or stroke or "brain damage" or "cerebrovascular event*" or "cerebrovascular accident*").tw,kf. | 340469 |  |
|  | 11 | ("brain injur*" adj2 (hypoxic or hypoxia or anoxic or ischemia or ischemic)).tw,kf. | 6095 |  |
|  | 12 | or/1-11 | 565316 | Combination line for brain injury concept combined with OR |
| MHSU | 13 | exp mental health/ or mental disorders/ or exp anxiety disorders/ or exp "bipolar and related disorders"/ or "disruptive, impulse control, and conduct disorders"/ or exp dissociative disorders/ or exp mood disorders/ or exp "attention deficit and disruptive behavior disorders"/ or exp personality disorders/ or exp "schizophrenia spectrum and other psychotic disorders"/ or somatoform disorders/ or exp conversion disorder/ or exp "trauma and stressor related disorders"/ or affective symptoms/ or depression/ or self-injurious behavior/ or suicide/ | 824041 | Subject headings, mental health and mental disorders |
|  | 14 | exp substance-related disorders/ or exp drinking behavior/ or exp drug-seeking behavior/ or impulsive behavior/ or exp "marijuana use"/ or exp "recreational drug use"/ or exp smoking/ or exp "tobacco use"/ or drug overdose/ | 503742 | Substance use and addiction related behaviour subject headings |
|  | 15 | (mental* adj2 (disorder* or illness* or "ill-health")).tw,kf. | 101458 | Proximity operators for key MH related terms |
|  | 16 | (psychological* adj2 (disorder* or illness* or distress or dysfunction)).tw,kf. | 34012 |  |
|  | 17 | addiction*.tw,kf. | 60582 | Keyword for addiction as a category term |
|  | 18 | (substance* adj2 (use* or abuse* or misuse*)).tw,kf. | 82429 | Proximity operators for key addiction related terms |
|  | 19 | (drug* adj2 (use* or abuse* or misuse*)).tw,kf. | 170096 |  |
|  | 20 | (overdose* adj1 (drug* or substance* or opiate* or opioid*)).tw,kf. | 6234 |  |
|  | 21 | (dependence adj2 (drug* or substance* or physical or psychological)).tw,kf. | 10901 |  |
|  | 22 | ("affective disorder*" or "agoraphobia" or "anxiety*" or "anxiety disorder*" or "acute stress" or depression* or depressed or "depressive disorder*" or "dysthymia" or "generalized anxiety disorder" or gad or "mania" or "mood disorder*" or mdd or "major depression" or "moderate depression" or "depressive symptom*" or "obsessive compulsive disorder" or "obsessive-compulsive disorder" or ocd or psychosis or phobias or "phobic disorder*" or "panic disorder*" or "panic attack*" or "post-traumatic stress" or "post-traumatic stress disorder" or ptsd or "social anxiety disorder").tw,kf. | 749903 | Keyword terms including acronyms, MH disorders  ^1^Research (see Scholten et al. 2006 for SR) indicates that anxiety disorders (namely, generalized anxiety disorder, acute stress disorder, panic disorder, agoraphobia, specific phobia, social phobia, obsessive-compulsive disorder [OCD], and PTSD) and depressive disorders (dysthymia, bipolar disorder, and MDD) are the most commonly observed MH disorders following ABI. |
|  | 23 | ("addictive behaviour*" or "polysubstance addiction*" or "dual addiction" or "substance use disorder*" or sud).tw,kf. | 22015 | Keyword terms including acronyms, addiction |
|  | 24 | or/13-23 | 1827642 | Combination line for MHSU concept combined with OR |
| Intervention | 25 | Program Evaluation/ or exp clinical protocol/ or exp guideline/ or guidelines as topic/ or exp practice guideline/ or practice guidelines as topic/ or health planning guidelines/ | 458084 | Relevant subject headings |
|  | 26 | (experimental or evaluat* or impact* or assess* or dif-dif or PSM or "double difference" or difference-in-difference or RDD or "difference in difference" or "statistical matching*" or "propensity score matching" or "covariate matching" or "coarsened-exact matching" or "propensity weighted" or "regression analysis" or "multiple regression" or "statistical regression" or "regression discontinuity*" or "experimental design" or "cohort analysis" or "quantitative method*" or "qualitative method*" or "program evaluation" or "program development" or "interrupted time series" or ITS or (before adj5 after) or (pre adj5 post) or ((pretest or "pre test") and (posttest or "post test")) or ((("fixed effect*" or "random effect*") adj3 (model or estimation)) or "instrumental variable" or "synthetic control") or ((quantitative or "comparison group*" or counterfactual or "counter factual" or counter-factual or experiment*) adj3 (design or study or analysis))).mp. | 12567617 | Search terms describing impact evaluations adapted from Barretta et al.’s (2021) evidence map on energy efficiency impact evaluations |
|  | 27 | Clinical Studies as Topic/ or exp Clinical Trial/ or exp Clinical Trials as Topic/ or Clinical Trial Protocol/ or Clinical Trial Protocols as Topic/ or Multicenter Study/ or Multicenter Studies as Topic/ or "Multicenter Study (topic)"/ or Randomization/ or Random Allocation/ or Double-Blind Method/ or Double Blind Procedure/ or Double-Blind Studies/ or Single-Blind Method/ or Single Blind Procedure/ or Single-Blind Studies/ or Placebos/ or Placebo/ or Control Groups/ or Control Group/ or Cross-Over Studies/ or Crossover Procedure/ or Non-Randomized Controlled Trials as Topic/ | 1574875 | Search string for clinical trails (ALL) adapted from CADTH (2021).  All Clinical Trials - MEDLINE, Embase, PsycInfo. In: CADTH Search Filters Database. Ottawa: CADTH; 2023: https://searchfilters.cadth.ca/link/34. Accessed 2023-01-04. |
|  | 28 | (pragmatic study or pragmatic studies).ti,ab,kf. | 574 |  |
|  | 29 | (pragmatic or practical) adj3 trial*.ti,ab,hw,kf. | 7495 |  |
|  | 30 | (random* or sham or placebo*).ti,ab,hw,kf. | 1782130 |  |
|  | 31 | ((singl* or doubl*) adj (blind* or dumm* or mask*)).ti,ab,hw,kf. | 264836 |  |
|  | 32 | ((tripl* or trebl*) adj (blind* or dumm* or mask*)).ti,ab,hw,kf. | 1548 |  |
|  | 33 | (control* adj3 (study or studies or trial* or group*)).ti,ab,hw,kf. | 1897175 |  |
|  | 34 | (clinical adj3 (study or studies or trial*)).ti,ab,hw,kf. | 1413034 |  |
|  | 35 | (Nonrandom* or non random* or non-random* or quasi-random* or quasirandom*).ti,ab,hw,kf. | 53506 |  |
|  | 36 | (phase adj3 (study or studies or trial*)).ti,ab,hw,kf. | 175158 |  |
|  | 37 | ((crossover or cross-over) adj3 (study or studies or trial*)).ti,ab,hw,kf. | 75987 |  |
|  | 38 | ((multicent* or multi-cent*) adj3 (study or studies or trial*)).ti,ab,hw,kf. | 402463 |  |
|  | 39 | allocated.ti,ab,hw. | 82073 |  |
|  | 40 | ((open label or open-label) adj5 (study or studies or trial*)).ti,ab,hw,kf. | 43727 |  |
|  | 41 | ((equivalence or superiority or non-inferiority or noninferiority) adj3 (study or studies or trial*)).ti,ab,hw,kf. | 11717 |  |
|  | 42 | ((quasiexperimental or quasi-experimental) adj3 (study or studies or trial*)).ti,ab,hw,kf. | 11738 |  |
|  | 43 | trial.ti,kf. | 300954 |  |
|  | 44 | or/25-43 | 14196564 | Combination line for intervention concept combined with OR |
|  | 45 | 12 and 24 and 44 | 25895 | Combination line: ABI AND MHSU AND Intervention |
|  | 46 | limit 45 to yr="2015 - current" | 11849 | Limited to 2015 to current |
|  | 47 | (address or autobiography or bibliography or biography or congress or dataset or dictionary or directory or editorial or "expression of concern" or festschrift or government document or interactive tutorial or interview or lecture or legal case or legislation or letter or news or newspaper article or overall or patient education handout or periodical index or personal narrative or portrait or webcasts).pt. | 2410555 | Irrelevant publication types |
|  | 48 | 46 not 47 | 11789 | Result after exclusion of irrelevant publication type |
|  | 49 | exp Animals/ not humans.sh. | 5101452 | Final result after excluding animal studies |
|  | 50 | 48 not 49 | 10893 |  |

| **DECISION-MAP Search Equation – Cochrane Central Register of Controlled Trials**  **(Ovid)** | | | | |
| --- | --- | --- | --- | --- |
| **Concept** | **#** | **Searches** | **Results** | **Annotations** |
| ABI | 1 | Brain Injuries/ | 2211 |  |
|  | 2 | exp brain hemorrhage, traumatic/ or exp brain injuries, diffuse/ or brain injuries, traumatic/ or brain concussion/ or brain contusion/ or brain injury, chronic/ | 1364 | Exploded subject heading for brain hemorrhage and brain injuries diffuse |
|  | 3 | craniocerebral trauma/ or head injuries, closed/ or head injuries, penetrating/ or intracranial hemorrhage, traumatic/ | 493 | Additional subject headings of relevance |
|  | 4 | exp brain ischemia/ or intracranial hemorrhages/ or exp stroke/ or exp hypoxia, brain/ | 15547 |  |
|  | 5 | ("traumatic brain injur*" or tbi or neurotrauma* or "neuro-trauma*").tw,kw. | 5138 | Keyword terms including acronyms  The concussion search string from the following paper were adapted and used: Schneider et al. (in press). The Amsterdam 2022 Process: A Summary of the Methodology for the 6th International Consensus Conference on Concussion in Sport. British Journal of Sports Medicine. |
|  | 6 | (head adj2 (injur* or impact* or trauma*)).tw,kw. | 2327 |  |
|  | 7 | (brain adj2 (injur* or impact* or trauma*)).tw,kw. | 7377 |  |
|  | 8 | (concussion* or concussed or concussive or "mild traumatic brain injur*" or mtbi).tw,kw. | 1311 |  |
|  | 9 | (craniocerebral adj2 (trauma* or injur*)).tw,kw. | 174 |  |
|  | 10 | ("acquired brain injur*" or abi or "non-traumatic brain injur*" or "non traumatic brain injur*" or stroke or "brain damage" or "cerebrovascular event*" or "cerebrovascular accident*").tw,kw. | 66164 |  |
|  | 11 | ("brain injur*" adj2 (hypoxic or hypoxia or anoxic or ischemia or ischemic)).tw,kw. | 214 |  |
|  | 12 | or/1-11 | 76741 | Combination line for brain injury concept combined with OR |
| MHSU | 13 | exp mental health/ or mental disorders/ or exp anxiety disorders/ or exp "bipolar and related disorders"/ or "disruptive, impulse control, and conduct disorders"/ or exp dissociative disorders/ or exp mood disorders/ or exp "attention deficit and disruptive behavior disorders"/ or exp personality disorders/ or exp "schizophrenia spectrum and other psychotic disorders"/ or somatoform disorders/ or exp conversion disorder/ or exp "trauma and stressor related disorders"/ or affective symptoms/ or depression/ or self-injurious behavior/ or suicide/ | 59517 | Exploded subject heading for mental health and mental disorders |
|  | 14 | exp substance-related disorders/ or exp drinking behavior/ or exp drug-seeking behavior/ or impulsive behavior/ or exp "marijuana use"/ or exp "recreational drug use"/ or exp smoking/ or exp "tobacco use"/ or drug overdose/ | 27623 | Substance use and addiction related behaviour subject headings |
|  | 15 | (mental* adj2 (disorder* or illness* or "ill-health")).tw,kw. | 12913 | Proximity operators for key MH related terms |
|  | 16 | (psychological* adj2 (disorder* or illness* or distress or dysfunction)).tw,kw. | 5428 |  |
|  | 17 | addiction*.tw,kw. | 8825 | Keyword for addiction as a category term |
|  | 18 | (substance* adj2 (use* or abuse* or misuse*)).tw,kw. | 9409 | Proximity operators for key addiction related terms |
|  | 19 | (drug* adj2 (use* or abuse* or misuse*)).tw,kw. | 20069 |  |
|  | 20 | (overdose* adj1 (drug* or substance* or opiate* or opioid*)).tw,kw. | 1008 |  |
|  | 21 | (dependence adj2 (drug* or substance* or physical or psychological)).tw,kw. | 2796 |  |
|  | 22 | ("affective disorder*" or "agoraphobia" or "anxiety*" or "anxiety disorder*" or "acute stress" or depression* or depressed or "depressive disorder*" or "dysthymia" or "generalized anxiety disorder" or gad or "mania" or "mood disorder*" or mdd or "major depression" or "moderate depression" or "depressive symptom*" or "obsessive compulsive disorder" or "obsessive-compulsive disorder" or ocd or psychosis or phobias or "phobic disorder*" or "panic disorder*" or "panic attack*" or "post-traumatic stress" or "post-traumatic stress disorder" or ptsd or "social anxiety disorder").tw,kw. | 139089 | Keyword terms including acronyms, MH disorders  ^1^Research (see Scholten et al. 2006 for SR) indicates that anxiety disorders (namely, generalized anxiety disorder, acute stress disorder, panic disorder, agoraphobia, specific phobia, social phobia, obsessive-compulsive disorder [OCD], and PTSD) and depressive disorders (dysthymia, bipolar disorder, and MDD) are the most commonly observed MH disorders following ABI. |
|  | 23 | ("addictive behaviour*" or "polysubstance addiction*" or "dual addiction" or "substance use disorder*" or sud).tw,kw. | 2472 | Keyword terms including acronyms, addiction |
|  | 24 | or/13-23 | 210340 | Combination line for MHSU concept combined with OR |
| Intervention | 25 | Program Evaluation/ or exp clinical protocol/ or exp guideline/ or guidelines as topic/ or exp practice guideline/ or practice guidelines as topic/ or health planning guidelines/ | 33377 | Relevant subject headings |
|  | 26 | (experimental or evaluat* or impact* or assess* or dif-dif or PSM or "double difference" or difference-in-difference or RDD or "difference in difference" or "statistical matching*" or "propensity score matching" or "covariate matching" or "coarsened-exact matching" or "propensity weighted" or "regression analysis" or "multiple regression" or "statistical regression" or "regression discontinuity*" or "experimental design" or "cohort analysis" or "quantitative method*" or "qualitative method*" or "program evaluation" or "program development" or "interrupted time series" or ITS or (before adj5 after) or (pre adj5 post) or ((pretest or "pre test") and (posttest or "post test")) or ((("fixed effect*" or "random effect*") adj3 (model or estimation)) or "instrumental variable" or "synthetic control") or ((quantitative or "comparison group*" or counterfactual or "counter factual" or counter-factual or experiment*) adj3 (design or study or analysis))).mp. | 1176973 | Search terms describing impact evaluations adapted from Barretta et al.’s (2021) evidence map on energy efficiency impact evaluations |
|  | 27 | Clinical Studies as Topic/ or exp Clinical Trial/ or exp Clinical Trials as Topic/ or Clinical Trial Protocol/ or Clinical Trial Protocols as Topic/ or Multicenter Study/ or Multicenter Studies as Topic/ or "Multicenter Study (topic)"/ or Randomization/ or Random Allocation/ or Double-Blind Method/ or Double Blind Procedure/ or Double-Blind Studies/ or Single-Blind Method/ or Single Blind Procedure/ or Single-Blind Studies/ or Placebos/ or Placebo/ or Control Groups/ or Control Group/ or Cross-Over Studies/ or Crossover Procedure/ or Non-Randomized Controlled Trials as Topic/ | 273326 | Search string for clinical trails (ALL) adapted from CADTH (2021).  All Clinical Trials - MEDLINE, Embase, PsycInfo. In: CADTH Search Filters Database. Ottawa: CADTH; 2023: https://searchfilters.cadth.ca/link/34. Accessed 2023-01-04. |
|  | 28 | (pragmatic study or pragmatic studies).ti,ab,kw. | 344 |  |
|  | 29 | (pragmatic or practical) adj3 trial*.ti,ab,hw,kw. | 6317 |  |
|  | 30 | (random* or sham or placebo*).ti,ab,hw,kw. | 1308013 |  |
|  | 31 | ((singl* or doubl*) adj (blind* or dumm* or mask*)).ti,ab,hw,kw. | 386946 |  |
|  | 32 | ((tripl* or trebl*) adj (blind* or dumm* or mask*)).ti,ab,hw,kw. | 2527 |  |
|  | 33 | (control* adj3 (study or studies or trial* or group*)).ti,ab,hw,kw. | 977728 |  |
|  | 34 | (clinical adj3 (study or studies or trial*)).ti,ab,hw,kw. | 673884 |  |
|  | 35 | (Nonrandom* or non random* or non-random* or quasi-random* or quasirandom*).ti,ab,hw,kw. | 10168 |  |
|  | 36 | (phase adj3 (study or studies or trial*)).ti,ab,hw,kw. | 132451 |  |
|  | 37 | ((crossover or cross-over) adj3 (study or studies or trial*)).ti,ab,hw,kw. | 90381 |  |
|  | 38 | ((multicent* or multi-cent*) adj3 (study or studies or trial*)).ti,ab,hw,kw. | 136137 |  |
|  | 39 | allocated.ti,ab,hw. | 72403 |  |
|  | 40 | ((open label or open-label) adj5 (study or studies or trial*)).ti,ab,hw,kw. | 52266 |  |
|  | 41 | ((equivalence or superiority or non-inferiority or noninferiority) adj3 (study or studies or trial*)).ti,ab,hw,kw. | 13839 |  |
|  | 42 | ((quasiexperimental or quasi-experimental) adj3 (study or studies or trial*)).ti,ab,hw,kw. | 5137 |  |
|  | 43 | trial.ti,kw. | 734167 |  |
|  | 44 | or/25-43 | 1681586 | Combination line for intervention concept combined with OR |
|  | 45 | 12 and 24 and 44 | 5543 | Combination line: ABI AND MHSU AND Intervention |
|  | 46 | limit 45 to yr="2015 - current" | 3478 | Limited to 2015 to current |
|  | 47 | (address or autobiography or bibliography or biography or congress or dataset or dictionary or directory or editorial or "expression of concern" or festschrift or government document or interactive tutorial or interview or lecture or legal case or legislation or letter or news or newspaper article or overall or patient education handout or periodical index or personal narrative or portrait or webcasts).pt. | 18844 | Irrelevant publication types |
|  | 48 | 46 not 47 | 3472 | Result after exclusion of irrelevant publication type |
|  | 49 | exp Animals/ not humans.sh. | 2683 | Animal filter |
|  | 50 | 48 not 49 | 3472 |  |

| **DECISION-MAP Search Equation –Cochrane Database of Systematic Reviews**  **(Ovid)** | | | | |
| --- | --- | --- | --- | --- |
| ABI | 1 | ("traumatic brain injur*" or tbi or neurotrauma* or "neuro-trauma*").ti,ab,kw. | 60 |  |
|  | 2 | (head adj2 (injur* or impact* or trauma*)).ti,ab,kw. | 60 | The concussion search string from the following paper were adapted and used: Schneider et al. (in press). The Amsterdam 2022 Process: A Summary of the Methodology for the 6th International Consensus Conference on Concussion in Sport. British Journal of Sports Medicine. |
|  | 3 | (brain adj2 (injur* or impact* or trauma*)).ti,ab,kw. | 84 |  |
|  | 4 | (concussion* or concussed or concussive or "mild traumatic brain injur*" or mtbi).ti,ab,kw. | 4 |  |
|  | 5 | (craniocerebral adj2 (trauma* or injur*)).ti,ab,kw. | 7 |  |
|  | 6 | ("acquired brain injur*" or abi or "non-traumatic brain injur*" or "non traumatic brain injur*" or stroke or "brain damage" or "cerebrovascular event*" or "cerebrovascular accident*").ti,ab,kw. | 543 |  |
|  | 7 | ("brain injur*" adj2 (hypoxic or hypoxia or anoxic or ischemia or ischemic)).ti,ab,kw. | 0 |  |
|  | 8 | or/1-7 | 623 | Combination line for brain injury concept combined with OR |
| MHSU | 9 | (mental* adj2 (disorder* or illness* or "ill-health")).ti,ab,kw. | 245 | Proximity operators for key MH related terms |
|  | 10 | (psychological* adj2 (disorder* or illness* or distress or dysfunction)).ti,ab,kw. | 56 |  |
|  | 11 | addiction*.ti,ab,kw. | 104 | Keyword for addiction as a category term |
|  | 12 | (substance* adj2 (use* or abuse* or misuse*)).ti,ab,kw. | 56 | Proximity operators for key addiction related terms |
|  | 13 | (drug* adj2 (use* or abuse* or misuse*)).ti,ab,kw. | 232 |  |
|  | 14 | (overdose* adj1 (drug* or substance* or opiate* or opioid*)).ti,ab,kw. | 2 |  |
|  | 15 | (dependence adj2 (drug* or substance* or physical or psychological)).ti,ab,kw. | 40 |  |
|  | 16 | ("affective disorder*" or "agoraphobia" or "anxiety*" or "anxiety disorder*" or "acute stress" or depression* or depressed or "depressive disorder*" or "dysthymia" or "generalized anxiety disorder" or gad or "mania" or "mood disorder*" or mdd or "major depression" or "moderate depression" or "depressive symptom*" or "obsessive compulsive disorder" or "obsessive-compulsive disorder" or ocd or psychosis or phobias or "phobic disorder*" or "panic disorder*" or "panic attack*" or "post-traumatic stress" or "post-traumatic stress disorder" or ptsd or "social anxiety disorder").ti,ab,kw. | 972 | Keyword terms including acronyms, MH disorders  ^1^Research (see Scholten et al. 2006 for SR) indicates that anxiety disorders (namely, generalized anxiety disorder, acute stress disorder, panic disorder, agoraphobia, specific phobia, social phobia, obsessive-compulsive disorder [OCD], and PTSD) and depressive disorders (dysthymia, bipolar disorder, and MDD) are the most commonly observed MH disorders following ABI. |
|  | 17 | ("addictive behaviour*" or "polysubstance addiction*" or "dual addiction" or "substance use disorder*" or sud).ti,ab,kw. | 15 | Keyword terms including acronyms, addiction |
|  | 18 | or/9-17 | 1388 | Combination line for MHSU concept combined with OR |
| Intervention | 19 | (experimental or evaluat* or impact* or assess* or dif-dif or PSM or "double difference" or difference-in-difference or RDD or "difference in difference" or "statistical matching*" or "propensity score matching" or "covariate matching" or "coarsened-exact matching" or "propensity weighted" or "regression analysis" or "multiple regression" or "statistical regression" or "regression discontinuity*" or "experimental design" or "cohort analysis" or "quantitative method*" or "qualitative method*" or "program evaluation" or "program development" or "interrupted time series" or ITS or (before adj5 after) or (pre adj5 post) or ((pretest or "pre test") and (posttest or "post test")) or ((("fixed effect*" or "random effect*") adj3 (model or estimation)) or "instrumental variable" or "synthetic control") or ((quantitative or "comparison group*" or counterfactual or "counter factual" or counter-factual or experiment*) adj3 (design or study or analysis))).mp. | 11006 | Search terms describing impact evaluations adapted from Barretta et al.’s (2021) evidence map on energy efficiency impact evaluations |
|  | 20 | (pragmatic study or pragmatic studies).ti,ab,kw. | 2 | Search string for clinical trails (ALL) adapted from CADTH (2021).  All Clinical Trials - MEDLINE, Embase, PsycInfo. In: CADTH Search Filters Database. Ottawa: CADTH; 2023: https://searchfilters.cadth.ca/link/34. Accessed 2023-01-04. |
|  | 21 | (pragmatic or practical) adj3 trial*.ti,ab,kw. | 25 |  |
|  | 22 | (random* or sham or placebo*).ti,ab,kw. | 8863 |  |
|  | 23 | ((singl* or doubl*) adj (blind* or dumm* or mask*)).ti,ab,kw. | 530 |  |
|  | 24 | ((tripl* or trebl*) adj (blind* or dumm* or mask*)).ti,ab,kw. | 1 |  |
|  | 25 | (control* adj3 (study or studies or trial* or group*)).ti,ab,kw. | 8579 |  |
|  | 26 | (clinical adj3 (study or studies or trial*)).ti,ab,kw. | 3426 |  |
|  | 27 | (Nonrandom* or non random* or non-random* or quasi-random* or quasirandom*).ti,ab,kw. | 1913 |  |
|  | 28 | (phase adj3 (study or studies or trial*)).ti,ab,kw. | 74 |  |
|  | 29 | ((crossover or cross-over) adj3 (study or studies or trial*)).ti,ab,kw. | 535 |  |
|  | 30 | ((multicent* or multi-cent*) adj3 (study or studies or trial*)).ti,ab,kw. | 180 |  |
|  | 31 | allocated.ti,ab. | 283 |  |
|  | 32 | ((open label or open-label) adj5 (study or studies or trial*)).ti,ab,kw. | 82 |  |
|  | 33 | ((equivalence or superiority or non-inferiority or noninferiority) adj3 (study or studies or trial*)).ti,ab,kw. | 18 |  |
|  | 34 | ((quasiexperimental or quasi-experimental) adj3 (study or studies or trial*)).ti,ab,kw. | 19 |  |
|  | 35 | trial.ti,kw. | 18 |  |
|  | 36 | or/19-35 | 11045 | Combination line for intervention concept combined with OR |
|  | 37 | 8 and 18 and 36 | 61 | Combination line: ABI AND MHSU AND Intervention |
|  | 38 | limit 37 to yr="2015 - current" | 24 | Limited to 2015 to current |

| **DECISION-MAP Search Equation – PsycINFO**  **(EBSCOhost)** | | | | |
| --- | --- | --- | --- | --- |
| ABI | S1 | DE "Brain Injuries" OR DE "Traumatic Brain Injury" OR DE "Brain Concussion" OR DE "Post-Concussive Symptoms" OR DE "Brain Damage" OR DE "Head Injuries" OR DE "Cerebral Ischemia" OR DE "Cerebral Infarction" OR DE "Cerebrovascular Accidents" OR DE "Anoxia" OR DE "Cerebral Hemorrhage" | 77,471 | Subject headings for ABI |
|  | S2 | TI ( ("traumatic brain injur*" OR "tbi" OR "neurotrauma*" OR "neuro-trauma*") ) OR AB ( ("traumatic brain injur*" OR "tbi" OR "neurotrauma*" OR "neuro-trauma*") ) OR KW ( ("traumatic brain injur*" OR "tbi" OR "neurotrauma*" OR "neuro-trauma*") ) | 21,479 | Keyword terms including acronyms  The concussion search string from the following paper were adapted and used: Schneider et al. (in press). The Amsterdam 2022 Process: A Summary of the Methodology for the 6th International Consensus Conference on Concussion in Sport. British Journal of Sports Medicine. |
|  | S3 | TI ( ("head" N2 ("injur*" OR "impact*" OR "trauma*")) ) OR AB ( ("head" N2 ("injur*" OR "impact*" OR "trauma*")) ) OR KW ( ("head" N2 ("injur*" OR "impact*" OR "trauma*")) ) | 9,132 |  |
|  | S4 | TI ( ("brain" N2 ("injur*" OR "impact*" OR "trauma*")) ) OR AB ( ("brain" N2 ("injur*" OR "impact*" OR "trauma*")) ) OR KW ( ("brain" N2 ("injur*" OR "impact*" OR "trauma*")) ) | 35,946 |  |
|  | S5 | TI ( ("concussion*" OR "concussed" OR "concussive" OR "mild traumatic brain injur*" OR "mtbi") ) OR AB ( ("concussion*" OR "concussed" OR "concussive" OR "mild traumatic brain injur*" OR "mtbi") ) OR KW ( ("concussion*" OR "concussed" OR "concussive" OR "mild traumatic brain injur*" OR "mtbi") ) | 6,809 |  |
|  | S6 | TI ( ("craniocerebral" N2 ("trauma*" OR "injur*")) ) OR AB ( ("craniocerebral" N2 ("trauma*" OR "injur*")) ) OR KW ( ("craniocerebral" N2 ("trauma*" OR "injur*")) ) | 211 |  |
|  | S7 | TI ( ("acquired brain injur*" OR "abi" OR "non-traumatic brain injur*" OR "non traumatic brain injur*" OR "stroke" OR "brain damage" OR "cerebrovascular event*" OR "cerebrovascular accident*") ) OR AB ( ("acquired brain injur*" OR "abi" OR "non-traumatic brain injur*" OR "non traumatic brain injur*" OR "stroke" OR "brain damage" OR "cerebrovascular event*" OR "cerebrovascular accident*") ) OR KW ( ("acquired brain injur*" OR "abi" OR "non-traumatic brain injur*" OR "non traumatic brain injur*" OR "stroke" OR "brain damage" OR "cerebrovascular event*" OR "cerebrovascular accident*") ) | 50,842 |  |
|  | S8 | TI ( ("brain injur*" N2 ("hypoxic" OR "hypoxia" OR "anoxic" OR "ischemia" OR "ischemic")) ) OR AB ( ("brain injur*" N2 ("hypoxic" OR "hypoxia" OR "anoxic" OR "ischemia" OR "ischemic")) ) OR KW ( ("brain injur*" N2 ("hypoxic" OR "hypoxia" OR "anoxic" OR "ischemia" OR "ischemic")) ) | 1,070 |  |
|  | S9 | S1 OR S2 OR S3 OR S4 OR S5 OR S6 OR S7 OR S8 | 105,254 | Combination line for brain injury concept combined with OR |
| MHSU | S10 | DE "Mental Health" OR DE "Mental Disorders" OR DE "Affective Disorders" OR DE "Anxiety Disorders" OR DE "Bipolar Disorder" OR DE "Borderline States" OR DE "Chronic Mental Illness" OR DE "Dissociative Disorders" OR DE "Attention Deficit Disorder" OR DE "Emotional and Behavioral Disorders "OR DE "Obsessive Compulsive Disorder" OR DE "Personality Disorders" OR DE "Psychosis" OR DE "Serious Mental Illness" OR DE "Sleep Wake Disorders" OR DE "Somatoform Disorders" OR DE "Stress and Trauma Related Disorders" OR DE "Thought Disorders" OR DE "Serious Mental Illness" OR DE "Major Depression" OR DE "Persistent Depressive Disorder" OR DE "Schizophrenia" OR DE "Acute Schizophrenia" OR DE "Schizoaffective Disorder" OR DE "Schizophreniform Disorder" OR DE "Nonsuicidal Self-Injury" OR DE "Self-Inflicted Wounds" OR DE "Suicide" OR DE "Conversion Disorder" OR DE "Psychosis" OR DE "Affective Psychosis" OR DE | 954,263 | Subject heading for mental health and mental disorders |
|  | S11 | DE "Substance Related and Addictive Disorders" OR DE "Addiction" OR DE "Drug Addiction" OR DE "Substance Use Disorder" OR DE "Alcohol Use Disorder" OR DE "Alcohol Abuse" OR DE "Cannabis Use Disorder" OR DE "Drug Abuse" OR DE "Drug Dependency" OR DE "Opioid Use Disorder" OR DE "Tobacco Use Disorder" OR DE "Substance Induced Psychotic Disorders" | 108,644 | Substance use and addiction related subject headings |
|  | S12 | TI ( ("mental*" N2 ("disorder*" OR "illness*" OR "ill-health")) ) OR AB ( ("mental*" N2 ("disorder*" OR "illness*" OR "ill-health")) ) OR KW ( ("mental*" N2 ("disorder*" OR "illness*" OR "ill-health")) ) | 123,733 | Proximity operators for key MH related terms |
|  | S13 | TI ( ("psychological*" N2 ("disorder*" OR "illness*" OR "distress" OR "dysfunction")) ) OR AB ( ("psychological*" N2 ("disorder*" OR "illness*" OR "distress" OR "dysfunction")) ) OR KW ( ("psychological*" N2 ("disorder*" OR "illness*" OR "distress" OR "dysfunction")) ) |  |  |
|  | S14 | TI "addiction*" OR AB "addiction*" OR KW "addiction*" | 51,850 | Keyword for addiction as a category term |
|  | S15 | TI ( ("substance*" N2 ("use*" OR "abuse*" OR "misuse*")) ) OR AB ( ("substance*" N2 ("use*" OR "abuse*" OR "misuse*")) ) OR KW ( ("substance*" N2 ("use*" OR "abuse*" OR "misuse*")) ) | 85,328 | Proximity operators for key addiction related terms |
|  | S16 | TI ( ("drug*" N2 ("use*" OR "abuse*" OR "misuse*")) ) OR AB ( ("drug*" N2 ("use*" OR "abuse*" OR "misuse*")) ) OR KW ( ("drug*" N2 ("use*" OR "abuse*" OR "misuse*")) ) | 77,909 |  |
|  | S17 | TI ( ("overdose*" N1 ("drug*" OR "substance*" OR "opiate*" OR "opioid*")) ) OR AB ( ("overdose*" N1 ("drug*" OR "substance*" OR "opiate*" OR "opioid*")) ) OR KW ( ("overdose*" N1 ("drug*" OR "substance*" OR "opiate*" OR "opioid*")) ) | 2,756 |  |
|  | S18 | TI ( ("dependence" N2 ("drug*" OR "substance*" OR "physical" OR "psychological")) ) OR AB ( ("dependence" N2 ("drug*" OR "substance*" OR "physical" OR "psychological")) ) OR KW ( ("dependence" N2 ("drug*" OR "substance*" OR "physical" OR "psychological")) ) | 10,669 |  |
|  | S19 | TI ( ("affective disorder*" OR "agoraphobia" OR "anxiety*" OR "anxiety disorder*" OR "acute stress" OR "depression*" OR "depressed" OR "depressive disorder*" OR "dysthymia" OR "generalized anxiety disorder" OR "gad" OR "mania" OR "mood disorder*" OR "mdd" OR "major depression" OR "moderate depression" OR "depressive symptom*" OR "obsessive compulsive disorder" OR "obsessive-compulsive disorder" OR "ocd" OR "psychosis" OR "phobias" OR "phobic disorder*" OR "panic disorder*" OR "panic attack*" OR "post-traumatic stress" OR "post-traumatic stress disorder" OR "ptsd" OR "social anxiety disorder") ) OR AB ( ("affective disorder*" OR "agoraphobia" OR "anxiety*" OR "anxiety disorder*" OR "acute stress" OR "depression*" OR "depressed" OR "depressive disorder*" OR "dysthymia" OR "generalized anxiety disorder" OR "gad" OR "mania" OR "mood disorder*" OR "mdd" OR "major depression" OR "moderate depression" OR "depressive symptom*" OR "obsessive compulsive disorder" OR "obsessive-compulsive disorder" OR "ocd" OR "psychosis" OR "phobias" OR "phobic disorder*" OR "panic disorder*" OR "panic attack*" OR "post-traumatic stress" OR "post-traumatic stress disorder" OR "ptsd" OR "social anxiety disorder") ) OR KW ( ("affective disorder*" OR "agoraphobia" OR "anxiety*" OR "anxiety disorder*" OR "acute stress" OR "depression*" OR "depressed" OR "depressive disorder*" OR "dysthymia" OR "generalized anxiety disorder" OR "gad" OR "mania" OR "mood disorder*" OR "mdd" OR "major depression" OR "moderate depression" OR "depressive symptom*" OR "obsessive compulsive disorder" OR "obsessive-compulsive disorder" OR "ocd" OR "psychosis" OR "phobias" OR "phobic disorder*" OR "panic disorder*" OR "panic attack*" OR "post-traumatic stress" OR "post-traumatic stress disorder" OR "ptsd" OR "social anxiety disorder") ) | 570,920 | Keyword terms including acronyms, MH disorders  ^1^Research (see Scholten et al. 2006 for SR) indicates that anxiety disorders (namely, generalized anxiety disorder, acute stress disorder, panic disorder, agoraphobia, specific phobia, social phobia, obsessive-compulsive disorder [OCD], and PTSD) and depressive disorders (dysthymia, bipolar disorder, and MDD) are the most commonly observed MH disorders following ABI. |
|  | S20 | TI ( ("addictive behaviour*" OR "polysubstance addiction*" OR "dual addiction" OR "substance use disorder*" OR "sud") ) OR AB ( ("addictive behaviour*" OR "polysubstance addiction*" OR "dual addiction" OR "substance use disorder*" OR "sud") ) OR KW ( ("addictive behaviour*" OR "polysubstance addiction*" OR "dual addiction" OR "substance use disorder*" OR "sud") ) | 20,360 | Keyword terms including acronyms, addiction |
|  | S21 | S10 OR S11 OR S12 OR S13 OR S14 OR S15 OR S16 OR S17 OR S18 OR S19 OR S20 | 1,386,989 | Combination line for MHSU concept combined with OR |
| Intervention | S22 | (DE "Mental Health Program Evaluation") OR (DE "Treatment Guidelines") | 11,345 | Relevant subject headings |
|  | S23 | TI ( ("experimental" OR "evaluat*" OR "impact*" OR "assess*" OR "dif-dif" OR "PSM" OR "double difference" OR "difference-in-difference" OR "RDD" OR "difference in difference" OR "statistical matching*" OR "propensity score matching" OR "covariate matching" OR "coarsened-exact matching" OR "propensity weighted" OR "regression analysis" OR "multiple regression" OR "statistical regression" OR "regression discontinuity*" OR "experimental design" OR "cohort analysis" OR "quantitative method*" OR "qualitative method*" OR "program evaluation" OR "program development" OR "interrupted time series" OR "ITS" OR ("before" N5 "after") OR ("pre" N5 "post") OR (("pretest" OR "pre test") and ("posttest" OR "post test")) OR ((("fixed effect*" OR "random effect*") N3 ("model" OR "estimation")) OR "instrumental variable" OR "synthetic control") OR (("quantitative" OR "comparison group*" OR "counterfactual" OR "counter factual" OR "counter-factual" OR "experiment*") N3 ("design" OR "study" OR "analysis"))) ) OR AB ( ("experimental" OR "evaluat*" OR "impact*" OR "assess*" OR "dif-dif" OR "PSM" OR "double difference" OR "difference-in-difference" OR "RDD" OR "difference in difference" OR "statistical matching*" OR "propensity score matching" OR "covariate matching" OR "coarsened-exact matching" OR "propensity weighted" OR "regression analysis" OR "multiple regression" OR "statistical regression" OR "regression discontinuity*" OR "experimental design" OR "cohort analysis" OR "quantitative method*" OR "qualitative method*" OR "program evaluation" OR "program development" OR "interrupted time series" OR "ITS" OR ("before" N5 "after") OR ("pre" N5 "post") OR (("pretest" OR "pre test") and ("posttest" OR "post test")) OR ((("fixed effect*" OR "random effect*") N3 ("model" OR "estimation")) OR "instrumental variable" OR "synthetic control") OR (("quantitative" OR "comparison group*" OR "counterfactual" OR "counter factual" OR "counter-factual" OR "experiment*") N3 ("design" OR "study" OR "analysis"))) ) OR KW ( ("experimental" OR "evaluat*" OR "impact*" OR "assess*" OR "dif-dif" OR "PSM" OR "double difference" OR "difference-in-difference" OR "RDD" OR "difference in difference" OR "statistical matching*" OR "propensity score matching" OR "covariate matching" OR "coarsened-exact matching" OR "propensity weighted" OR "regression analysis" OR "multiple regression" OR "statistical regression" OR "regression discontinuity*" OR "experimental design" OR "cohort analysis" OR "quantitative method*" OR "qualitative method*" OR "program evaluation" OR "program development" OR "interrupted time series" OR "ITS" OR ("before" N5 "after") OR ("pre" N5 "post") OR (("pretest" OR "pre test") and ("posttest" OR "post test")) OR ((("fixed effect*" OR "random effect*") N3 ("model" OR "estimation")) OR "instrumental variable" OR "synthetic control") OR (("quantitative" OR "comparison group*" OR "counterfactual" OR "counter factual" OR "counter-factual" OR "experiment*") N3 ("design" OR "study" OR "analysis"))) ) | 2,292,259 | Search terms describing impact evaluations adapted from Barretta et al.’s (2021) evidence map on energy efficiency impact evaluations |
|  | S24 | (((MH "Experimental Studies+") OR (MH "Multicenter Studies") OR (MH "Random Sample+") OR (MH "Placebos") OR (MH "Control (Research)+") OR (MH "Crossover Design") OR ((TI random* OR AB random*) OR (TI sham OR AB sham) OR (TI placebo* OR AB placebo*)) OR (((TI singl* OR AB singl*) OR (TI doubl* OR AB doubl*)) W1 ((TI blind* OR AB blind*) OR (TI dumm* OR AB dumm*) OR (TI mask* OR AB mask*))) OR (((TI tripl* OR AB tripl*) OR (TI trebl* OR AB trebl*)) W1 ((TI blind* OR AB blind*) OR (TI dumm* OR AB dumm*) OR (TI mask* OR AB mask*))) OR ((TI control* OR AB control*) N3 ((TI study OR AB study) OR (TI studies OR AB studies) OR (TI trial* OR AB trial*) OR (TI group* OR AB group*))) OR ((TI clinical OR AB clinical) N3 ((TI study OR AB study) OR (TI studies OR AB studies) OR (TI trial* OR AB trial*))) OR ((TI Nonrandom* OR AB Nonrandom*) OR (TI "non random*" OR AB "non random*") OR (TI "non-random*" OR AB "non-random*") OR (TI "quasi-random*" OR AB "quasi-random*") OR (TI quasirandom* OR AB quasirandom*)) OR ((TI phase OR AB phase) N3 ((TI study OR AB study) OR (TI studies OR AB studies) OR (TI trial* OR AB trial*))) OR (((TI crossover OR AB crossover) OR (TI "cross-over" OR AB "cross-over")) N3 ((TI study OR AB study) OR (TI studies OR AB studies) OR (TI trial* OR AB trial*))) OR (((TI multicent* OR AB multicent*) OR (TI "multi-cent*" OR AB "multi-cent*")) N3 ((TI study OR AB study) OR (TI studies OR AB studies) OR (TI trial* OR AB trial*))) OR (TI allocated OR AB allocated) OR (((TI "open label" OR AB "open label") OR (TI "open-label" OR AB "open-label")) N5 ((TI study OR AB study) OR (TI studies OR AB studies) OR (TI trial* OR AB trial*))) OR (((TI equivalence OR AB equivalence) OR (TI superiority OR AB superiority) OR (TI "non-inferiority" OR AB "non-inferiority") OR (TI noninferiority OR AB noninferiority)) N3 ((TI study OR AB study) OR (TI studies OR AB studies) OR (TI trial* OR AB trial*))) OR ((TI "pragmatic study" OR AB "pragmatic study") OR (TI "pragmatic studies" OR AB "pragmatic studies")) OR (((TI pragmatic OR AB pragmatic) OR (TI practical OR AB practical)) N3 (TI trial* OR AB trial*)) OR (((TI quasiexperimental OR AB quasiexperimental) OR (TI "quasi-experimental" OR AB "quasi-experimental")) N3 ((TI study OR AB study) OR (TI studies OR AB studies) OR (TI trial* OR AB trial*))) OR TI trial) | 473,358 | Search string for clinical trails (ALL) adapted from CADTH (2021).  All Clinical Trials - MEDLINE, Embase, PsycInfo. In: CADTH Search Filters Database. Ottawa: CADTH; 2023: https://searchfilters.cadth.ca/link/34. Accessed 2023-01-04. |
|  | S25 | S22 OR S23 OR S24 | 2,478,252 | Combination line for intervention concept combined with OR |
|  | S26 | S9 AND S21 AND S25 | 16,008 | Combination line: ABI AND MHSU AND Intervention |
|  | 46 | Limit publication Year: 2015–2023, limit to academic journal articles | 5,726 | Final result after limiting by publication date and type |

| **DECISION-MAP Search Equation – CINHAL Complete**  **(EBSCOhost)** | | | | |
| --- | --- | --- | --- | --- |
| ABI | S1 | (MH "Brain Injuries+") OR (MH "Brain Concussion+") OR (MH "Brain Contusions+") OR (MH "Brain Damage, Chronic+") OR (MH "Hypoxia-Ischemia, Brain+") OR (MH "Head Injuries+") OR (MH "Stroke+") | 85,863 | Subject headings for ABI |
|  | S2 | TI ( ("traumatic brain injur*" OR "tbi" OR "neurotrauma*" OR "neuro-trauma*") ) OR AB ( ("traumatic brain injur*" OR "tbi" OR "neurotrauma*" OR "neuro-trauma*") ) | 20,613 | Keyword terms including acronyms,  The concussion search string from the following paper were adapted and used: Schneider et al. (in press). The Amsterdam 2022 Process: A Summary of the Methodology for the 6th International Consensus Conference on Concussion in Sport. British Journal of Sports Medicine. |
|  | S3 | TI ( ("head" N2 ("injur*" OR "impact*" OR "trauma*")) ) OR AB ( ("head" N2 ("injur*" OR "impact*" OR "trauma*")) ) | 11,948 |  |
|  | S4 | TI ( ("brain" N2 ("injur*" OR "impact*" OR "trauma*")) ) OR AB ( ("brain" N2 ("injur*" OR "impact*" OR "trauma*")) ) | 31,910 |  |
|  | S5 | TI ( ("concussion*" OR "concussed" OR "concussive" OR "mild traumatic brain injur*" OR "mtbi") ) OR AB ( ("concussion*" OR "concussed" OR "concussive" OR "mild traumatic brain injur*" OR "mtbi") ) | 8,623 |  |
|  | S6 | TI ( ("craniocerebral" N2 ("trauma*" OR "injur*")) ) OR AB ( ("craniocerebral" N2 ("trauma*" OR "injur*")) ) | 206 |  |
|  | S7 | TI ( ("acquired brain injur*" OR "abi" OR "non-traumatic brain injur*" OR "non traumatic brain injur*" OR "stroke" OR "brain damage" OR "cerebrovascular event*" OR "cerebrovascular accident*") ) OR AB ( ("acquired brain injur*" OR "abi" OR "non-traumatic brain injur*" OR "non traumatic brain injur*" OR "stroke" OR "brain damage" OR "cerebrovascular event*" OR "cerebrovascular accident*") ) | 119,862 |  |
|  | S8 | TI ( ("brain injur*" N2 ("hypoxic" OR "hypoxia" OR "anoxic" OR "ischemia" OR "ischemic")) ) OR AB ( ("brain injur*" N2 ("hypoxic" OR "hypoxia" OR "anoxic" OR "ischemia" OR "ischemic")) ) | 1,253 |  |
|  | S9 | S1 OR S2 OR S3 OR S4 OR S5 OR S6 OR S7 OR S8 | 188,397 | Combination line for brain injury concept combined with OR |
| MHSU | S10 | (MH "Mental Health") OR (MH "Research, Mental Health" OR MH "Behavioral Symptoms") OR (MH "Affective Symptoms+") OR (MH "Agitation") OR (MH "Communicative Disorders+") OR (MH "Compulsive Behavior+") OR (MH "Eating Disorders+") OR (MH "Social Behavior Disorders+") OR (MH "Stress+") OR (MH "Suicide+") OR (MH "Adjustment Disorders+") OR (MH "Mental Disorders, Chronic") OR (MH "Organic Mental Disorders+") OR (MH "Neurotic Disorders+") OR (MH "Personality Disorders+") OR (MH "Psychotic Disorders+") OR (MH "Psychological Trauma+") | 664,008 | Subject heading for mental health and mental disorders |
|  | S11 | (MH "Behavior, Addictive") OR (MH "Drug-Seeking Behavior") OR (MH "Substance Dependence+") OR (MH "Substance Use Disorders+") | 190,698 | Substance use and addiction related subject headings |
|  | S12 | TI ( ("mental*" N2 ("disorder*" OR "illness*" OR "ill-health")) ) OR AB ( ("mental*" N2 ("disorder*" OR "illness*" OR "ill-health")) ) | 45,844 | Proximity operators for key MH related terms |
|  | S13 | TI ( ("psychological*" N2 ("disorder*" OR "illness*" OR "distress" OR "dysfunction")) ) OR AB ( ("psychological*" N2 ("disorder*" OR "illness*" OR "distress" OR "dysfunction")) ) | 17,717 |  |
|  | S14 | TI "addiction*" OR AB "addiction*" | 25,150 | Keyword for addiction as a category term |
|  | S15 | TI ( ("substance*" N2 ("use*" OR "abuse*" OR "misuse*")) ) OR AB ( ("substance*" N2 ("use*" OR "abuse*" OR "misuse*")) ) | 47,557 | Proximity operators for key addiction related terms |
|  | S16 | TI ( ("drug*" N2 ("use*" OR "abuse*" OR "misuse*")) ) OR AB ( ("drug*" N2 ("use*" OR "abuse*" OR "misuse*")) ) | 59,464 |  |
|  | S17 | TI ( ("overdose*" N1 ("drug*" OR "substance*" OR "opiate*" OR "opioid*")) ) OR AB ( ("overdose*" N1 ("drug*" OR "substance*" OR "opiate*" OR "opioid*")) ) | 4,255 |  |
|  | S18 | TI ( ("dependence" N2 ("drug*" OR "substance*" OR "physical" OR "psychological")) ) OR AB ( ("dependence" N2 ("drug*" OR "substance*" OR "physical" OR "psychological")) ) | 3,866 |  |
|  | S19 | TI ( ("affective disorder*" OR "agoraphobia" OR "anxiety*" OR "anxiety disorder*" OR "acute stress" OR "depression*" OR "depressed" OR "depressive disorder*" OR "dysthymia" OR "generalized anxiety disorder" OR "gad" OR "mania" OR "mood disorder*" OR "mdd" OR "major depression" OR "moderate depression" OR "depressive symptom*" OR "obsessive compulsive disorder" OR "obsessive-compulsive disorder" OR "ocd" OR "psychosis" OR "phobias" OR "phobic disorder*" OR "panic disorder*" OR "panic attack*" OR "post-traumatic stress" OR "post-traumatic stress disorder" OR "ptsd" OR "social anxiety disorder") ) OR AB ( ("affective disorder*" OR "agoraphobia" OR "anxiety*" OR "anxiety disorder*" OR "acute stress" OR "depression*" OR "depressed" OR "depressive disorder*" OR "dysthymia" OR "generalized anxiety disorder" OR "gad" OR "mania" OR "mood disorder*" OR "mdd" OR "major depression" OR "moderate depression" OR "depressive symptom*" OR "obsessive compulsive disorder" OR "obsessive-compulsive disorder" OR "ocd" OR "psychosis" OR "phobias" OR "phobic disorder*" OR "panic disorder*" OR "panic attack*" OR "post-traumatic stress" OR "post-traumatic stress disorder" OR "ptsd" OR "social anxiety disorder") ) | 262,736 | Keyword terms including acronyms, MH disorders  ^1^Research (see Scholten et al. 2006 for SR) indicates that anxiety disorders (namely, generalized anxiety disorder, acute stress disorder, panic disorder, agoraphobia, specific phobia, social phobia, obsessive-compulsive disorder [OCD], and PTSD) and depressive disorders (dysthymia, bipolar disorder, and MDD) are the most commonly observed MH disorders following ABI. |
|  | S20 | TI ( ("addictive behaviour*" OR "polysubstance addiction*" OR "dual addiction" OR "substance use disorder*" OR "sud") ) OR AB ( ("addictive behaviour*" OR "polysubstance addiction*" OR "dual addiction" OR "substance use disorder*" OR "sud") ) | 12,638 | Keyword terms including acronyms, addiction |
|  | S21 | S10 OR S11 OR S12 OR S13 OR S14 OR S15 OR S16 OR S17 OR S18 OR S19 OR S20 | 965,433 | Combination line for MHSU concept combined with OR |
| Intervention | S22 | TI ( ("experimental" OR "evaluat*" OR "impact*" OR "assess*" OR "dif-dif" OR "PSM" OR "double difference" OR "difference-in-difference" OR "RDD" OR "difference in difference" OR "statistical matching*" OR "propensity score matching" OR "covariate matching" OR "coarsened-exact matching" OR "propensity weighted" OR "regression analysis" OR "multiple regression" OR "statistical regression" OR "regression discontinuity*" OR "experimental design" OR "cohort analysis" OR "quantitative method*" OR "qualitative method*" OR "program evaluation" OR "program development" OR "interrupted time series" OR "ITS" OR ("before" N5 "after") OR ("pre" N5 "post") OR (("pretest" OR "pre test") and ("posttest" OR "post test")) OR ((("fixed effect*" OR "random effect*") N3 ("model" OR "estimation")) OR "instrumental variable" OR "synthetic control") OR (("quantitative" OR "comparison group*" OR "counterfactual" OR "counter factual" OR "counter-factual" OR "experiment*") N3 ("design" OR "study" OR "analysis"))) ) OR AB ( ("experimental" OR "evaluat*" OR "impact*" OR "assess*" OR "dif-dif" OR "PSM" OR "double difference" OR "difference-in-difference" OR "RDD" OR "difference in difference" OR "statistical matching*" OR "propensity score matching" OR "covariate matching" OR "coarsened-exact matching" OR "propensity weighted" OR "regression analysis" OR "multiple regression" OR "statistical regression" OR "regression discontinuity*" OR "experimental design" OR "cohort analysis" OR "quantitative method*" OR "qualitative method*" OR "program evaluation" OR "program development" OR "interrupted time series" OR "ITS" OR ("before" N5 "after") OR ("pre" N5 "post") OR (("pretest" OR "pre test") and ("posttest" OR "post test")) OR ((("fixed effect*" OR "random effect*") N3 ("model" OR "estimation")) OR "instrumental variable" OR "synthetic control") OR (("quantitative" OR "comparison group*" OR "counterfactual" OR "counter factual" OR "counter-factual" OR "experiment*") N3 ("design" OR "study" OR "analysis"))) ) | 2,386,592 | Search terms describing impact evaluations adapted from Barretta et al.’s (2021) evidence map on energy efficiency impact evaluations |
|  | S23 | (((MH "Experimental Studies+") OR (MH "Multicenter Studies") OR (MH "Random Sample+") OR (MH "Placebos") OR (MH "Control (Research)+") OR (MH "Crossover Design") OR ((TI random* OR AB random*) OR (TI sham OR AB sham) OR (TI placebo* OR AB placebo*)) OR (((TI singl* OR AB singl*) OR (TI doubl* OR AB doubl*)) W1 ((TI blind* OR AB blind*) OR (TI dumm* OR AB dumm*) OR (TI mask* OR AB mask*))) OR (((TI tripl* OR AB tripl*) OR (TI trebl* OR AB trebl*)) W1 ((TI blind* OR AB blind*) OR (TI dumm* OR AB dumm*) OR (TI mask* OR AB mask*))) OR ((TI control* OR AB control*) N3 ((TI study OR AB study) OR (TI studies OR AB studies) OR (TI trial* OR AB trial*) OR (TI group* OR AB group*))) OR ((TI clinical OR AB clinical) N3 ((TI study OR AB study) OR (TI studies OR AB studies) OR (TI trial* OR AB trial*))) OR ((TI Nonrandom* OR AB Nonrandom*) OR (TI "non random*" OR AB "non random*") OR (TI "non-random*" OR AB "non-random*") OR (TI "quasi-random*" OR AB "quasi-random*") OR (TI quasirandom* OR AB quasirandom*)) OR ((TI phase OR AB phase) N3 ((TI study OR AB study) OR (TI studies OR AB studies) OR (TI trial* OR AB trial*))) OR (((TI crossover OR AB crossover) OR (TI "cross-over" OR AB "cross-over")) N3 ((TI study OR AB study) OR (TI studies OR AB studies) OR (TI trial* OR AB trial*))) OR (((TI multicent* OR AB multicent*) OR (TI "multi-cent*" OR AB "multi-cent*")) N3 ((TI study OR AB study) OR (TI studies OR AB studies) OR (TI trial* OR AB trial*))) OR (TI allocated OR AB allocated) OR (((TI "open label" OR AB "open label") OR (TI "open-label" OR AB "open-label")) N5 ((TI study OR AB study) OR (TI studies OR AB studies) OR (TI trial* OR AB trial*))) OR (((TI equivalence OR AB equivalence) OR (TI superiority OR AB superiority) OR (TI "non-inferiority" OR AB "non-inferiority") OR (TI noninferiority OR AB noninferiority)) N3 ((TI study OR AB study) OR (TI studies OR AB studies) OR (TI trial* OR AB trial*))) OR ((TI "pragmatic study" OR AB "pragmatic study") OR (TI "pragmatic studies" OR AB "pragmatic studies")) OR (((TI pragmatic OR AB pragmatic) OR (TI practical OR AB practical)) N3 (TI trial* OR AB trial*)) OR (((TI quasiexperimental OR AB quasiexperimental) OR (TI "quasi-experimental" OR AB "quasi-experimental")) N3 ((TI study OR AB study) OR (TI studies OR AB studies) OR (TI trial* OR AB trial*))) OR TI trial) | 1,224,209 | Search string for clinical trails (ALL) adapted from CADTH (2021).  All Clinical Trials - MEDLINE, Embase, PsycInfo. In: CADTH Search Filters Database. Ottawa: CADTH; 2023: https://searchfilters.cadth.ca/link/34. Accessed 2023-01-04. |
|  | S24 | S22 OR S23 | 2,908,413 | Combination line for intervention concept combined with OR |
|  | S25 | S9 AND S21 AND S24 | 14,213 | Combination line: ABI AND MHSU AND Intervention |
|  | S26 | Published Date: 20150101-20230431 | 7,320 | Limited to 2015 to current and limit to academic journals |
|  | S27 | (MH "Animals+") | 35,702 | Animal filter |
|  | S28 | (MH "Human") | 1,188,722 |  |
|  | S29 | S27 NOT S28 | 33,074 |  |
|  | S30 | S26 NOT S29 | 7,258 | Final result after exclusion of animals |

| **DECISION-MAP Search Equation – Web of Science**  **(Clarivate)** | | | | |  |  |
| --- | --- | --- | --- | --- | --- | --- |
| ABI | 1 | TS=(( ("traumatic brain injur*" OR "tbi" OR "neurotrauma*" OR "neuro-trauma*") ) ) | 80741 | Keyword terms including acronyms  The concussion search string from the following paper were adapted and used: Schneider et al. (in press). The Amsterdam 2022 Process: A Summary of the Methodology for the 6th International Consensus Conference on Concussion in Sport. British Journal of Sports Medicine. |  |  |
|  | 2 | TS=(( ("head" NEAR/2 ("injur*" OR "impact*" OR "trauma*")) ) ) | 56369 |  |  |  |
|  | 3 | TS=(( ("brain" NEAR/2 ("injur*" OR "impact*" OR "trauma*")) ) ) | 135280 |  |  |  |
|  | 4 | TS=(( ("concussion*" OR "concussed" OR "concussive" OR "mild traumatic brain injur*" OR "mtbi") ) ) | 22235 |  |  |  |
|  | 5 | TS=(( ("craniocerebral" NEAR/2 ("trauma*" OR "injur*")) ) ) | 1958 |  |  |  |
|  | 6 | TS=(( ("acquired brain injur*" OR "abi" OR "non-traumatic brain injur*" OR "non traumatic brain injur*" OR "stroke" OR "brain damage" OR "cerebrovascular event*" OR "cerebrovascular accident*") )) | 458546 |  |  |  |
|  | 7 | TS=(( ("brain injur*" NEAR/2 ("hypoxic" OR "hypoxia" OR "anoxic" OR "ischemia" OR "ischemic")) ) ) | 8339 |  |  |  |
|  | 8 | #7 OR #6 OR #5 OR #4 OR #3 OR #2 OR #1 | 612895 |  |  |  |
| MHSU | 9 | TS=(( ("mental*" NEAR/2 ("disorder*" OR "illness*" OR "ill-health")) ) ) | 127368 | Proximity operators and keyword terms for MHSU and related terms |  |  |
|  | 10 | TS=(( ("psychological*" NEAR/2 ("disorder*" OR "illness*" OR "distress" OR "dysfunction")) ) ) | 52872 |  |  |  |
|  | 11 | TS=("addiction*" OR AB "addiction*" OR KW "addiction*") | 88512 |  |  |  |
|  | 12 | TS=(( ("substance*" NEAR/2 ("use*" OR "abuse*" OR "misuse*")) ) ) | 127999 |  |  |  |
|  | 13 | TS=(( ("drug*" NEAR/2 ("use*" OR "abuse*" OR "misuse*")) ) ) | 239109 |  |  |  |
|  | 14 | TS=(( ("overdose*" NEAR/1 ("drug*" OR "substance*" OR "opiate*" OR "opioid*")) ) ) | 7115 |  |  |  |
|  | 15 | TS=(( ("dependence" NEAR/2 ("drug*" OR "substance*" OR "physical" OR "psychological")) ) ) | 16127 |  |  |  |
|  | 16 | TS=(( ("affective disorder*" OR "agoraphobia" OR "anxiety*" OR "anxiety disorder*" OR "acute stress" OR "depression*" OR "depressed" OR "depressive disorder*" OR "dysthymia" OR "generalized anxiety disorder" OR "gad" OR "mania" OR "mood disorder*" OR "mdd" OR "major depression" OR "moderate depression" OR "depressive symptom*" OR "obsessive compulsive disorder" OR "obsessive-compulsive disorder" OR "ocd" OR "psychosis" OR "phobias" OR "phobic disorder*" OR "panic disorder*" OR "panic attack*" OR "post-traumatic stress" OR "post-traumatic stress disorder" OR "ptsd" OR "social anxiety disorder") ) ) | 1054082 | Keyword terms including acronyms, MH disorders  ^1^Research (see Scholten et al. 2006 for SR) indicates that anxiety disorders (namely, generalized anxiety disorder, acute stress disorder, panic disorder, agoraphobia, specific phobia, social phobia, obsessive-compulsive disorder [OCD], and PTSD) and depressive disorders (dysthymia, bipolar disorder, and MDD) are the most commonly observed MH disorders following ABI. |  |  |
|  | 17 | TS=(( ("addictive behaviour*" OR "polysubstance addiction*" OR "dual addiction" OR "substance use disorder*" OR "sud") ) ) | 31327 | Keyword terms, addiction |  |  |
|  | 18 | #17 OR #16 OR #15 OR #14 OR #13 OR #12 OR #11 OR #10 OR #9 | 1489783 | Combination line for MHSU concept combined with OR |  |  |
| Intervention | 19 | TS=(( ("experimental" OR "evaluat*" OR "impact*" OR "assess*" OR "dif-dif" OR "PSM" OR "double difference" OR "difference-in-difference" OR "RDD" OR "difference in difference" OR "statistical matching*" OR "propensity score matching" OR "covariate matching" OR "coarsened-exact matching" OR "propensity weighted" OR "regression analysis" OR "multiple regression" OR "statistical regression" OR "regression discontinuity*" OR "experimental design" OR "cohort analysis" OR "quantitative method*" OR "qualitative method*" OR "program evaluation" OR "program development" OR "interrupted time series" OR "ITS" OR ("before" NEAR/5 "after") OR ("pre" NEAR/5 "post") OR (("pretest" OR "pre test") and ("posttest" OR "post test")) OR ((("fixed effect*" OR "random effect*") NEAR/3 ("model" OR "estimation")) OR "instrumental variable" OR "synthetic control") OR (("quantitative" OR "comparison group*" OR "counterfactual" OR "counter factual" OR "counter-factual" OR "experiment*") NEAR/3 ("design" OR "study" OR "analysis"))) ) ) | 22017690 | Search terms describing impact evaluations adapted from Barretta et al.’s (2021) evidence map on energy efficiency impact evaluations |  |  |
|  | 20 | TS=(("pragmatic study" OR "pragmatic studies")) | 777 |  |  |  |
|  | 21 | TS=(("pragmatic" OR "practical") NEAR/1 "trial*") | 3965 | Search string for clinical trails (ALL) adapted from CADTH (2021).  All Clinical Trials - MEDLINE, Embase, PsycInfo. In: CADTH Search Filters Database. Ottawa: CADTH; 2023: https://searchfilters.cadth.ca/link/34. Accessed 2023-01-04. |  |  |
|  | 22 | TS=(("random*" OR "sham" OR "placebo*")) | 2456472 |  |  |  |
|  | 23 | TS=((("singl*" OR "doubl*") NEAR ("blind*" OR "dumm*" OR "mask*"))) | 356868 |  |  |  |
|  | 24 | TS=((("tripl*" OR "trebl*") NEAR ("blind*" OR "dumm*" OR "mask*"))) | 2347 |  |  |  |
|  | 25 | TS=(("control*" NEAR/3 ("study" OR "studies" OR "trial*" OR "group*"))) | 1422647 |  |  |  |
|  | 26 | TS=(("clinical" NEAR/3 ("study" OR "studies" OR "trial*"))) | 815702 |  |  |  |
|  | 27 | TS=(("Nonrandom*" OR "non random*" OR "non-random*" OR "quasi-random*" OR "quasirandom*")) | 60250 |  |  |  |
|  | 28 | TS=(("phase" NEAR/3 ("study" OR "studies" OR "trial*"))) | 291653 |  |  |  |
|  | 29 | TS=((("crossover" OR "cross-over") NEAR/3 ("study" OR "studies" OR "trial*"))) | 50767 |  |  |  |
|  | 30 | TS=((("multicent*" OR "multi-cent*") NEAR/3 ("study" OR "studies" OR "trial*"))) | 181741 |  |  |  |
|  | 31 | TS=("allocated") | 112498 |  |  |  |
|  | 32 | TS=((("open label" OR "open-label") NEAR/5 ("study" OR "studies" OR "trial*"))) | 51899 |  |  |  |
|  | 33 | TS=((("equivalence" OR "superiority" OR "non-inferiority" OR "noninferiority") NEAR/3 ("study" OR "studies" OR "trial*"))) | 16073 |  |  |  |
|  | 34 | TS=((("quasiexperimental" OR "quasi-experimental") NEAR/3 ("study" OR "studies" OR "trial*"))) | 15625 |  |  |  |
|  | 35 | TS=("trial") | 1325040 |  |  |  |
|  | 36 | #35 OR #34 OR #33 OR #32 OR #31 OR #30 OR #29 OR #28 OR #27 OR #26 OR #25 OR #24 OR #23 OR #22 OR #21 OR #20 OR #19 | 24080251 | Combination line for intervention concept combined with OR |  |  |
|  | 45 | #36 AND #18 AND #8 | 24,077 | Combination line: ABI AND MHSU AND Intervention |  |  |
|  | 46 | #36 AND #18 AND #8 and 2020 or 2019 or 2018 or 2017 or 2016 or 2015 or 2021 or 2022 or 2023 (Publication Years) | 13,290 | Limited to 2015 to current |  |  |
|  | 47 | NOT Document Types: Proceeding Paper or Meeting Abstract or Editorial Material or Letter or Book Review or News Item or Poetry or Software Review or Art Exhibit Review or Bibliography or Fiction, Creative Prose or Meeting or Film Review or Meeting Summary or Tv Review Radio Review Video or Music Score or TV Review, Radio Review Video or Dance Performance Review or Music Performance Review or Hardware Review or Theater Review or Biographical-Item or Note. | 12,995 | Final result after excluding irrelevant publication types |  |  |

| **DECISION-MAP Search Equation – SCOPUS**  **(Elsevier)** | | | | |
| --- | --- | --- | --- | --- |
| ABI | 1 | TITLE-ABS-KEY ( ( "traumatic brain injur*" OR "tbi" OR "neurotrauma*" OR "neuro-trauma*" ) ) | 77,778 | Keyword terms including acronyms  The concussion search string from the following paper were adapted and used: Schneider et al. (in press). The Amsterdam 2022 Process: A Summary of the Methodology for the 6th International Consensus Conference on Concussion in Sport. British Journal of Sports Medicine. |
|  | 2 | TITLE-ABS-KEY ( ( ( ("head") W/2 ( "injur*" OR "impact*" OR "trauma*" ) ) ) ) | 864 |  |
|  | 3 | TITLE-ABS-KEY ( ( ( ( "brain") W/2 ( "injur*" OR "impact*" OR "trauma*" ) ) ) ) | 1,947 |  |
|  | 4 | TITLE-ABS-KEY ( ( ( "concussion*" OR "concussed" OR "concussive" OR "mild traumatic brain injur*" OR "mtbi" ) ) ) | 23,781 |  |
|  | 5 | TITLE-ABS-KEY ( ( ( ("craniocerebral") W/2 ( "trauma*" OR "injur*" ) ) ) ) | 58 |  |
|  | 6 | TITLE-ABS-KEY ( ( ( "acquired brain injur*" OR "abi" OR "non-traumatic brain injur*" OR "non traumatic brain injur*" OR "stroke" OR "brain damage" OR "cerebrovascular event*" OR "cerebrovascular accident*" ) ) ) | 647,480 |  |
|  | 7 | TITLE-ABS-KEY ( ( ( ("brain injur*") W/2 ( "hypoxic" OR "hypoxia" OR "anoxic" OR "ischemia" OR "ischemic" ) ) ) ) | 245 |  |
|  | 8 | ( TITLE-ABS-KEY ( ( ( ( "brain injur*" ) W/2 ( "hypoxic" OR "hypoxia" OR "anoxic" OR "ischemia" OR "ischemic" ) ) ) ) ) OR ( TITLE-ABS-KEY ( ( ( "acquired brain injur*" OR "abi" OR "non-traumatic brain injur*" OR "non traumatic brain injur*" OR "stroke" OR "brain damage" OR "cerebrovascular event*" OR "cerebrovascular accident*" ) ) ) ) OR ( TITLE-ABS-KEY ( ( ( ( "craniocerebral" ) W/2 ( "trauma*" OR "injur*" ) ) ) ) ) OR ( TITLE-ABS-KEY ( ( ( "concussion*" OR "concussed" OR "concussive" OR "mild traumatic brain injur*" OR "mtbi" ) ) ) ) OR ( TITLE-ABS-KEY ( ( ( ( "brain" ) W/2 ( "injur*" OR "impact*" OR "trauma*" ) ) ) ) ) OR ( TITLE-ABS-KEY ( ( ( ( "head" ) W/2 ( "injur*" OR "impact*" OR "trauma*" ) ) ) ) ) OR ( TITLE-ABS-KEY ( ( "traumatic brain injur*" OR "tbi" OR "neurotrauma*" OR "neuro-trauma*" ) ) ) | 728,077 | Combination line for brain injury concept combined with OR |
| MHSU | 9 | TITLE-ABS-KEY ( ( ( ("mental*") W/2 ( "disorder*" OR "illness*" OR "ill-health" ) ) ) ) | 702 | Proximity operators for key MH related terms |
|  | 10 | TITLE-ABS-KEY ( ( ( ("psychological*") W/2 ( "disorder*" OR "illness*" OR "distress" OR "dysfunction" ) ) ) ) | 406 |  |
|  | 11 | TITLE-ABS-KEY ( "addiction*" ) | 166,677 | Keyword for addiction as a category term |
|  | 12 | TITLE-ABS-KEY ( ( ( ("substance*") W/2 ( "use*" OR "abuse*" OR "misuse*" ) ) ) ) | 1,436 | Proximity operators for key addiction related terms |
|  | 13 | TITLE-ABS-KEY ( ( ( ("drug*") W/2 ( "use*" OR "abuse*" OR "misuse*" ) ) ) ) | 11,720 |  |
|  | 14 | TITLE-ABS-KEY ( ( ( ("overdose*") W/1 ( "drug*" OR "substance*" OR "opiate*" OR "opioid*" ) ) ) ) | 105 |  |
|  | 16 | TITLE-ABS-KEY ( ( ( ("dependence") W/2 ( "drug*" OR "substance*" OR "physical" OR "psychological" ) ) ) ) | 3,198 |  |
|  | 17 | TITLE-ABS-KEY ( ( ( "affective disorder*" OR "agoraphobia" OR "anxiety*" OR "anxiety disorder*" OR "acute stress" OR "depression*" OR "depressed" OR "depressive disorder*" OR "dysthymia" OR "generalized anxiety disorder" OR "gad" OR "mania" OR "mood disorder*" OR "mdd" OR "major depression" OR "moderate depression" OR "depressive symptom*" OR "obsessive compulsive disorder" OR "obsessive-compulsive disorder" OR "ocd" OR "psychosis" OR "phobias" OR "phobic disorder*" OR "panic disorder*" OR "panic attack*" OR "post-traumatic stress" OR "post-traumatic stress disorder" OR "ptsd" OR "social anxiety disorder" ) ) ) | 1,442,707 | Keyword terms including acronyms, MH disorders  ^1^Research (see Scholten et al. 2006 for SR) indicates that anxiety disorders (namely, generalized anxiety disorder, acute stress disorder, panic disorder, agoraphobia, specific phobia, social phobia, obsessive-compulsive disorder [OCD], and PTSD) and depressive disorders (dysthymia, bipolar disorder, and MDD) are the most commonly observed MH disorders following ABI. |
|  | 18 | TITLE-ABS-KEY ( ( ( "addictive behaviour*" OR "polysubstance addiction*" OR "dual addiction" OR "substance use disorder*" OR "sud" ) ) ) | 35,682 | Keyword terms including acronyms, addiction |
|  | 19 | (TITLE-ABS-KEY ( ( ( ( ( "dependence*" ) W/2 ( "drug*" OR "substance*" OR "physical" OR "psychological" ) ) ) ) ) ) OR ( TITLE-ABS-KEY ( ( ( "addictive behaviour*" OR "polysubstance addiction*" OR "dual addiction" OR "substance use disorder*" OR "sud" ) ) ) ) OR ( TITLE-ABS-KEY ( ( ( "affective disorder*" OR "agoraphobia" OR "anxiety*" OR "anxiety disorder*" OR "acute stress" OR "depression*" OR "depressed" OR "depressive disorder*" OR "dysthymia" OR "generalized anxiety disorder" OR "gad" OR "mania" OR "mood disorder*" OR "mdd" OR "major depression" OR "moderate depression" OR "depressive symptom*" OR "obsessive compulsive disorder" OR "obsessive-compulsive disorder" OR "ocd" OR "psychosis" OR "phobias" OR "phobic disorder*" OR "panic disorder*" OR "panic attack*" OR "post-traumatic stress" OR "post-traumatic stress disorder" OR "ptsd" OR "social anxiety disorder" ) ) ) ) OR ( TITLE-ABS-KEY ( ( ( ( "dependence" ) W/2 ( "drug*" OR "substance*" OR "physical" OR "psychological" ) ) ) ) ) OR ( TITLE-ABS-KEY ( ( ( ( "overdose*" ) W/1 ( "drug*" OR "substance*" OR "opiate*" OR "opioid*" ) ) ) ) ) OR ( TITLE-ABS-KEY ( ( ( ( "drug*" ) W/2 ( "use*" OR "abuse*" OR "misuse*" ) ) ) ) ) OR ( TITLE-ABS-KEY ( ( ( ( "substance*" ) W/2 ( "use*" OR "abuse*" OR "misuse*" ) ) ) ) ) OR ( TITLE-ABS-KEY ( "addiction*" ) ) OR ( TITLE-ABS-KEY ( ( ( ( "psychological*" ) W/2 ( "disorder*" OR "illness*" OR "distress" OR "dysfunction" ) ) ) ) ) OR ( TITLE-ABS-KEY ( ( ( ( "mental*" ) W/2 ( "disorder*" OR "illness*" OR "ill-health" ) ) ) ) ) | 1,615,550 | Combination line for MHSU concept combined with OR |
| Intervention | 20 | TITLE-ABS-KEY ( ( ( "experimental" OR "evaluat*" OR "impact*" OR "assess*" OR "dif-dif" OR "PSM" OR "double difference" OR "difference-in-difference" OR "RDD" OR "difference in difference" OR "statistical matching*" OR "propensity score matching" OR "covariate matching" OR "coarsened-exact matching" OR "propensity weighted" OR "regression analysis" OR "multiple regression" OR "statistical regression" OR "regression discontinuity*" OR "experimental design" OR "cohort analysis" OR "quantitative method*" OR "qualitative method*" OR "program evaluation" OR "program development" OR "interrupted time series" OR "ITS" OR ( ("before") W/5 "after" ) OR ( ("pre") W/5 "post" ) OR ( ( "pretest" OR "pre test" ) AND ( "posttest" OR "post test" ) ) OR ( ( ( "fixed effect*" OR ("random effect*") ) W/3 ( "model" OR "estimation" ) ) OR "instrumental variable" OR "synthetic control" ) OR ( ( "quantitative" OR "comparison group*" OR "counterfactual" OR "counter factual" OR "counter-factual" OR ("experiment*" )) W/3 ( "design" OR "study" OR "analysis" ) ) ) ) ) | 30,710,951 | Search terms describing impact evaluations adapted from Barretta et al.’s (2021) evidence map on energy efficiency impact evaluations |
|  | 21 | TITLE-ABS-KEY ( random* OR sham OR placebo* ) OR TITLE-ABS-KEY ( ( singl* OR doubl* ) W/1 ( blind* OR dumm* OR mask* ) ) OR TITLE-ABS-KEY ( ( tripl* OR trebl* ) W/1 ( blind* OR dumm* OR mask* ) ) OR TITLE-ABS-KEY ( ( control* ) W/3 ( study OR studies OR trial* OR group* ) ) OR TITLE-ABS-KEY ( ( clinical ) W/3 ( study OR studies OR trial* ) ) OR TITLE-ABS-KEY ( nonrandom* OR "non random*" OR non-random* OR quasi-random* OR quasirandom* ) OR TITLE-ABS-KEY ( ( phase ) W/3 ( study OR studies OR trial* ) ) OR TITLE-ABS-KEY ( ( crossover OR cross-over ) W/3 ( study OR studies OR trial* ) ) OR TITLE-ABS-KEY ( ( multicent* OR multi-cent* ) W/3 ( study OR studies OR trial* ) ) OR TITLE-ABS ( allocated ) OR TITLE-ABS-KEY ( ( "open label" OR open-label ) W/5 ( study OR studies OR trial* ) ) OR TITLE-ABS-KEY ( ( equivalence OR superiority OR non-inferiority OR noninferiority ) W/3 ( study OR studies OR trial* ) ) OR TITLE-ABS-KEY ( "pragmatic study" OR "pragmatic studies" ) OR TITLE-ABS-KEY ( ( pragmatic OR practical ) W/3 ( trial* ) ) OR TITLE-ABS-KEY ( ( quasiexperimental OR quasi-experimental ) W/3 ( study OR studies OR trial* ) ) OR TITLE ( trial ) OR KEY ( trial ) |  | Search string for clinical trails (ALL) adapted from CADTH (2021).  All Clinical Trials - Scopus. In: CADTH Search Filters Database. Ottawa: CADTH; 2023: https://searchfilters.cadth.ca/link/106. Accessed 2023-03-10. |
|  | 22 | ( TITLE-ABS-KEY ( random* OR sham OR placebo* ) OR TITLE-ABS-KEY ( ( singl* OR doubl* ) W/1 ( blind* OR dumm* OR mask* ) ) OR TITLE-ABS-KEY ( ( tripl* OR trebl* ) W/1 ( blind* OR dumm* OR mask* ) ) OR TITLE-ABS-KEY ( ( control* ) W/3 ( study OR studies OR trial* OR group* ) ) OR TITLE-ABS-KEY ( ( clinical ) W/3 ( study OR studies OR trial* ) ) OR TITLE-ABS-KEY ( nonrandom* OR "non random*" OR non-random* OR quasi-random* OR quasirandom* ) OR TITLE-ABS-KEY ( ( phase ) W/3 ( study OR studies OR trial* ) ) OR TITLE-ABS-KEY ( ( crossover OR cross-over ) W/3 ( study OR studies OR trial* ) ) OR TITLE-ABS-KEY ( ( multicent* OR multi-cent* ) W/3 ( study OR studies OR trial* ) ) OR TITLE-ABS ( allocated ) OR TITLE-ABS-KEY ( ( "open label" OR open-label ) W/5 ( study OR studies OR trial* ) ) OR TITLE-ABS-KEY ( ( equivalence OR superiority OR non-inferiority OR noninferiority ) W/3 ( study OR studies OR trial* ) ) OR TITLE-ABS-KEY ( "pragmatic study" OR "pragmatic studies" ) OR TITLE-ABS-KEY ( ( pragmatic OR practical ) W/3 ( trial* ) ) OR TITLE-ABS-KEY ( ( quasiexperimental OR quasi-experimental ) W/3 ( study OR studies OR trial* ) ) OR TITLE ( trial ) OR KEY ( trial ) ) OR ( TITLE-ABS-KEY ( ( ( "experimental" OR "evaluat*" OR "impact*" OR "assess*" OR "dif-dif" OR "PSM" OR "double difference" OR "difference-in-difference" OR "RDD" OR "difference in difference" OR "statistical matching*" OR "propensity score matching" OR "covariate matching" OR "coarsened-exact matching" OR "propensity weighted" OR "regression analysis" OR "multiple regression" OR "statistical regression" OR "regression discontinuity*" OR "experimental design" OR "cohort analysis" OR "quantitative method*" OR "qualitative method*" OR "program evaluation" OR "program development" OR "interrupted time series" OR "ITS" OR ( ( "before" ) W/5 "after" ) OR ( ( "pre" ) W/5 "post" ) OR ( ( "pretest" OR "pre test" ) AND ( "posttest" OR "post test" ) ) OR ( ( ( "fixed effect*" OR ( "random effect*" ) ) W/3 ( "model" OR "estimation" ) ) OR "instrumental variable" OR "synthetic control" ) OR ( ( "quantitative" OR "comparison group*" OR "counterfactual" OR "counter factual" OR "counter-factual" OR ( "experiment*" ) ) W/3 ( "design" OR "study" OR "analysis" ) ) ) ) ) ) | 37,214,522 | Combination line for intervention concept combined with OR |
|  | 23 | ( ( TITLE-ABS-KEY ( random* OR sham OR placebo* ) OR TITLE-ABS-KEY ( ( singl* OR doubl* ) W/1 ( blind* OR dumm* OR mask* ) ) OR TITLE-ABS-KEY ( ( tripl* OR trebl* ) W/1 ( blind* OR dumm* OR mask* ) ) OR TITLE-ABS-KEY ( ( control* ) W/3 ( study OR studies OR trial* OR group* ) ) OR TITLE-ABS-KEY ( ( clinical ) W/3 ( study OR studies OR trial* ) ) OR TITLE-ABS-KEY ( nonrandom* OR "non random*" OR non-random* OR quasi-random* OR quasirandom* ) OR TITLE-ABS-KEY ( ( phase ) W/3 ( study OR studies OR trial* ) ) OR TITLE-ABS-KEY ( ( crossover OR cross-over ) W/3 ( study OR studies OR trial* ) ) OR TITLE-ABS-KEY ( ( multicent* OR multi-cent* ) W/3 ( study OR studies OR trial* ) ) OR TITLE-ABS ( allocated ) OR TITLE-ABS-KEY ( ( "open label" OR open-label ) W/5 ( study OR studies OR trial* ) ) OR TITLE-ABS-KEY ( ( equivalence OR superiority OR non-inferiority OR noninferiority ) W/3 ( study OR studies OR trial* ) ) OR TITLE-ABS-KEY ( "pragmatic study" OR "pragmatic studies" ) OR TITLE-ABS-KEY ( ( pragmatic OR practical ) W/3 ( trial* ) ) OR TITLE-ABS-KEY ( ( quasiexperimental OR quasi-experimental ) W/3 ( study OR studies OR trial* ) ) OR TITLE ( trial ) OR KEY ( trial ) ) OR ( TITLE-ABS-KEY ( ( ( "experimental" OR "evaluat*" OR "impact*" OR "assess*" OR "dif-dif" OR "PSM" OR "double difference" OR "difference-in-difference" OR "RDD" OR "difference in difference" OR "statistical matching*" OR "propensity score matching" OR "covariate matching" OR "coarsened-exact matching" OR "propensity weighted" OR "regression analysis" OR "multiple regression" OR "statistical regression" OR "regression discontinuity*" OR "experimental design" OR "cohort analysis" OR "quantitative method*" OR "qualitative method*" OR "program evaluation" OR "program development" OR "interrupted time series" OR "ITS" OR ( ( "before" ) W/5 "after" ) OR ( ( "pre" ) W/5 "post" ) OR ( ( "pretest" OR "pre test" ) AND ( "posttest" OR "post test" ) ) OR ( ( ( "fixed effect*" OR ( "random effect*" ) ) W/3 ( "model" OR "estimation" ) ) OR "instrumental variable" OR "synthetic control" ) OR ( ( "quantitative" OR "comparison group*" OR "counterfactual" OR "counter factual" OR "counter-factual" OR ( "experiment*" ) ) W/3 ( "design" OR "study" OR "analysis" ) ) ) ) ) ) ) AND ( ( TITLE-ABS-KEY ( ( ( ( ( "dependence*" ) W/2 ( "drug*" OR "substance*" OR "physical" OR "psychological" ) ) ) ) ) ) OR ( TITLE-ABS-KEY ( ( ( "addictive behaviour*" OR "polysubstance addiction*" OR "dual addiction" OR "substance use disorder*" OR "sud" ) ) ) ) OR ( TITLE-ABS-KEY ( ( ( "affective disorder*" OR "agoraphobia" OR "anxiety*" OR "anxiety disorder*" OR "acute stress" OR "depression*" OR "depressed" OR "depressive disorder*" OR "dysthymia" OR "generalized anxiety disorder" OR "gad" OR "mania" OR "mood disorder*" OR "mdd" OR "major depression" OR "moderate depression" OR "depressive symptom*" OR "obsessive compulsive disorder" OR "obsessive-compulsive disorder" OR "ocd" OR "psychosis" OR "phobias" OR "phobic disorder*" OR "panic disorder*" OR "panic attack*" OR "post-traumatic stress" OR "post-traumatic stress disorder" OR "ptsd" OR "social anxiety disorder" ) ) ) ) OR ( TITLE-ABS-KEY ( ( ( ( "dependence" ) W/2 ( "drug*" OR "substance*" OR "physical" OR "psychological" ) ) ) ) ) OR ( TITLE-ABS-KEY ( ( ( ( "overdose*" ) W/1 ( "drug*" OR "substance*" OR "opiate*" OR "opioid*" ) ) ) ) ) OR ( TITLE-ABS-KEY ( ( ( ( "drug*" ) W/2 ( "use*" OR "abuse*" OR "misuse*" ) ) ) ) ) OR ( TITLE-ABS-KEY ( ( ( ( "substance*" ) W/2 ( "use*" OR "abuse*" OR "misuse*" ) ) ) ) ) OR ( TITLE-ABS-KEY ( "addiction*" ) ) OR ( TITLE-ABS-KEY ( ( ( ( "psychological*" ) W/2 ( "disorder*" OR "illness*" OR "distress" OR "dysfunction" ) ) ) ) ) OR ( TITLE-ABS-KEY ( ( ( ( "mental*" ) W/2 ( "disorder*" OR "illness*" OR "ill-health" ) ) ) ) ) ) AND ( ( TITLE-ABS-KEY ( ( ( ( "brain injur*" ) W/2 ( "hypoxic" OR "hypoxia" OR "anoxic" OR "ischemia" OR "ischemic" ) ) ) ) ) OR ( TITLE-ABS-KEY ( ( ( "acquired brain injur*" OR "abi" OR "non-traumatic brain injur*" OR "non traumatic brain injur*" OR "stroke" OR "brain damage" OR "cerebrovascular event*" OR "cerebrovascular accident*" ) ) ) ) OR ( TITLE-ABS-KEY ( ( ( ( "craniocerebral" ) W/2 ( "trauma*" OR "injur*" ) ) ) ) ) OR ( TITLE-ABS-KEY ( ( ( "concussion*" OR "concussed" OR "concussive" OR "mild traumatic brain injur*" OR "mtbi" ) ) ) ) OR ( TITLE-ABS-KEY ( ( ( ( "brain" ) W/2 ( "injur*" OR "impact*" OR "trauma*" ) ) ) ) ) OR ( TITLE-ABS-KEY ( ( ( ( "head" ) W/2 ( "injur*" OR "impact*" OR "trauma*" ) ) ) ) ) OR ( TITLE-ABS-KEY ( ( "traumatic brain injur*" OR "tbi" OR "neurotrauma*" OR "neuro-trauma*" ) ) ) ) | 49,143 | Combination line: ABI AND MHSU AND Intervention |
|  | 24 | ( ( TITLE-ABS-KEY ( random* OR sham OR placebo* ) OR TITLE-ABS-KEY ( ( singl* OR doubl* ) W/1 ( blind* OR dumm* OR mask* ) ) OR TITLE-ABS-KEY ( ( tripl* OR trebl* ) W/1 ( blind* OR dumm* OR mask* ) ) OR TITLE-ABS-KEY ( ( control* ) W/3 ( study OR studies OR trial* OR group* ) ) OR TITLE-ABS-KEY ( ( clinical ) W/3 ( study OR studies OR trial* ) ) OR TITLE-ABS-KEY ( nonrandom* OR "non random*" OR non-random* OR quasi-random* OR quasirandom* ) OR TITLE-ABS-KEY ( ( phase ) W/3 ( study OR studies OR trial* ) ) OR TITLE-ABS-KEY ( ( crossover OR cross-over ) W/3 ( study OR studies OR trial* ) ) OR TITLE-ABS-KEY ( ( multicent* OR multi-cent* ) W/3 ( study OR studies OR trial* ) ) OR TITLE-ABS ( allocated ) OR TITLE-ABS-KEY ( ( "open label" OR open-label ) W/5 ( study OR studies OR trial* ) ) OR TITLE-ABS-KEY ( ( equivalence OR superiority OR non-inferiority OR noninferiority ) W/3 ( study OR studies OR trial* ) ) OR TITLE-ABS-KEY ( "pragmatic study" OR "pragmatic studies" ) OR TITLE-ABS-KEY ( ( pragmatic OR practical ) W/3 ( trial* ) ) OR TITLE-ABS-KEY ( ( quasiexperimental OR quasi-experimental ) W/3 ( study OR studies OR trial* ) ) OR TITLE ( trial ) OR KEY ( trial ) ) OR ( TITLE-ABS-KEY ( ( ( "experimental" OR "evaluat*" OR "impact*" OR "assess*" OR "dif-dif" OR "PSM" OR "double difference" OR "difference-in-difference" OR "RDD" OR "difference in difference" OR "statistical matching*" OR "propensity score matching" OR "covariate matching" OR "coarsened-exact matching" OR "propensity weighted" OR "regression analysis" OR "multiple regression" OR "statistical regression" OR "regression discontinuity*" OR "experimental design" OR "cohort analysis" OR "quantitative method*" OR "qualitative method*" OR "program evaluation" OR "program development" OR "interrupted time series" OR "ITS" OR ( ( "before" ) W/5 ("after" )) OR ( ( "pre" ) W/5 ("post") ) OR ( ( "pretest" OR "pre test" ) AND ( "posttest" OR "post test" ) ) OR ( ( ( "fixed effect*" OR ( "random effect*" ) ) W/3 ( "model" OR "estimation" ) ) OR "instrumental variable" OR "synthetic control" ) OR ( ( "quantitative" OR "comparison group*" OR "counterfactual" OR "counter factual" OR "counter-factual" OR ( "experiment*" ) ) W/3 ( "design" OR "study" OR "analysis" ) ) ) ) ) ) ) AND ( ( TITLE-ABS-KEY ( ( ( ( ( "dependence*" ) W/2 ( "drug*" OR "substance*" OR "physical" OR "psychological" ) ) ) ) ) ) OR ( TITLE-ABS-KEY ( ( ( "addictive behaviour*" OR "polysubstance addiction*" OR "dual addiction" OR "substance use disorder*" OR "sud" ) ) ) ) OR ( TITLE-ABS-KEY ( ( ( "affective disorder*" OR "agoraphobia" OR "anxiety*" OR "anxiety disorder*" OR "acute stress" OR "depression*" OR "depressed" OR "depressive disorder*" OR "dysthymia" OR "generalized anxiety disorder" OR "gad" OR "mania" OR "mood disorder*" OR "mdd" OR "major depression" OR "moderate depression" OR "depressive symptom*" OR "obsessive compulsive disorder" OR "obsessive-compulsive disorder" OR "ocd" OR "psychosis" OR "phobias" OR "phobic disorder*" OR "panic disorder*" OR "panic attack*" OR "post-traumatic stress" OR "post-traumatic stress disorder" OR "ptsd" OR "social anxiety disorder" ) ) ) ) OR ( TITLE-ABS-KEY ( ( ( ( "dependence" ) W/2 ( "drug*" OR "substance*" OR "physical" OR "psychological" ) ) ) ) ) OR ( TITLE-ABS-KEY ( ( ( ( "overdose*" ) W/1 ( "drug*" OR "substance*" OR "opiate*" OR "opioid*" ) ) ) ) ) OR ( TITLE-ABS-KEY ( ( ( ( "drug*" ) W/2 ( "use*" OR "abuse*" OR "misuse*" ) ) ) ) ) OR ( TITLE-ABS-KEY ( ( ( ( "substance*" ) W/2 ( "use*" OR "abuse*" OR "misuse*" ) ) ) ) ) OR ( TITLE-ABS-KEY ( "addiction*" ) ) OR ( TITLE-ABS-KEY ( ( ( ( "psychological*" ) W/2 ( "disorder*" OR "illness*" OR "distress" OR "dysfunction" ) ) ) ) ) OR ( TITLE-ABS-KEY ( ( ( ( "mental*" ) W/2 ( "disorder*" OR "illness*" OR "ill-health" ) ) ) ) ) ) AND ( ( TITLE-ABS-KEY ( ( ( ( "brain injur*" ) W/2 ( "hypoxic" OR "hypoxia" OR "anoxic" OR "ischemia" OR "ischemic" ) ) ) ) ) OR ( TITLE-ABS-KEY ( ( ( "acquired brain injur*" OR "abi" OR "non-traumatic brain injur*" OR "non traumatic brain injur*" OR "stroke" OR "brain damage" OR "cerebrovascular event*" OR "cerebrovascular accident*" ) ) ) ) OR ( TITLE-ABS-KEY ( ( ( ( "craniocerebral" ) W/2 ( "trauma*" OR "injur*" ) ) ) ) ) OR ( TITLE-ABS-KEY ( ( ( "concussion*" OR "concussed" OR "concussive" OR "mild traumatic brain injur*" OR "mtbi" ) ) ) ) OR ( TITLE-ABS-KEY ( ( ( ( "brain" ) W/2 ( "injur*" OR "impact*" OR "trauma*" ) ) ) ) ) OR ( TITLE-ABS-KEY ( ( ( ( "head" ) W/2 ( "injur*" OR "impact*" OR "trauma*" ) ) ) ) ) OR ( TITLE-ABS-KEY ( ( "traumatic brain injur*" OR "tbi" OR "neurotrauma*" OR "neuro-trauma*" ) ) ) ) AND ( LIMIT-TO ( PUBYEAR , 2023 ) OR LIMIT-TO ( PUBYEAR , 2022 ) OR LIMIT-TO ( PUBYEAR , 2021 ) OR LIMIT-TO ( PUBYEAR , 2020 ) OR LIMIT-TO ( PUBYEAR , 2019 ) OR LIMIT-TO ( PUBYEAR , 2018 ) OR LIMIT-TO ( PUBYEAR , 2017 ) OR LIMIT-TO ( PUBYEAR , 2016 ) OR LIMIT-TO ( PUBYEAR , 2015 ) ) AND ( EXCLUDE ( DOCTYPE , "ch" ) OR EXCLUDE ( DOCTYPE , "cp" ) OR EXCLUDE ( DOCTYPE , "no" ) OR EXCLUDE ( DOCTYPE , "sh" ) OR EXCLUDE ( DOCTYPE , "bk" ) OR EXCLUDE ( DOCTYPE , "cr" ) OR EXCLUDE ( DOCTYPE , "tb" ) OR EXCLUDE ( DOCTYPE , "dp" ) ) | 22,587 | Publication date limited to 2015 – current; irrelevant publication types removed (i.e., note, book chapter, conference paper, short survey, book, conference review, retracted, and data paper) |

| *2.2* Supplementary Table 2. *Study Characteristics for Inclusion and Exclusion.* | | |
| --- | --- | --- |
| DECISION–MAP: Study Eligibility Criteria | | |
|  | | |
| Study characteristic | Inclusion criteria | Exclusion criteria |
| Population | Adults (≥18 years of age) receiving treatment for one or more mental health and/or substance-use/addiction concern(s)*, who have a self-reported or confirmed history of ABI of any type from the following categories:   - Mild, moderate, or severe TBI (including concussion) - Cerebrovascular incident, stroke - Hypoxic and/or anoxic brain injury | - <18 years of age - Neoplasm or post-surgical brain injury - Infection (e.g., meningitis, encephalitis) or other inflammation (e.g., vasculitis, cerebral oedema) type brain injury - ABI populations not receiving MHSU intervention - Caregivers of those with ABI |
| Intervention | Any intervention specifically aimed to ameliorate mental health *and/or* addiction problems in ABI populations | - Any intervention not relevant to MHSU, or where MHSU is not the primary focus - Physical rehabilitation where MHSU is not the primary focus - Interventions for caregivers only |
| Comparator | Any comparator (e.g., control group, waiting list, active control, usual care, etc.) or no comparator | Not applicable |
| Outcomes | Any outcome that contributes to understanding MHSU interventions for persons with ABI is considered relevant | Any outcome not relevant to MHSU, or where MHSU is not the primary focus |
| Timing | Any | Not applicable |
| Setting | Any (e.g., in-patient, out-patient, community-based, acute treatment facilitates, etc.) | Not applicable |
| Study designs | Quantitative, qualitative, or mixed-method impact evaluations^†^, including:   - Randomized controlled trials (and variants), quasi-experimental designs (e.g., difference-in-differences, regression discontinuity, propensity score matching, etc.), and non-experimental impact evaluation designs (e.g., difference of means, before-after comparison, benchmarking, etc.)   Systematic reviews, meta-analyses, registered study protocols and clinical practice guidelines** | - Not an impact evaluation study (e.g., editorial, letter to the editor, conference paper, etc.) - Non-systematic reviews |
| Language | English | Non-English language publications |
| Country | Any | Not applicable |
| Publication date | 2014 onwards | Articles published 2013 and prior |
| Publication type | Full publication in a peer-reviewed journal or registered study protocol | Government reports, letters, editorials, reviews, dissertations, meeting abstracts, etc. (grey literature) |
| *Confirmed diagnosis of MHSU disorder is not required  ^†^According to the Impact and Innovation Unit of Impact Canada (2019) guidelines for impact measurement  ***“Clinical practice guidelines are systematically developed statements to assist practitioner and patient decisions about appropriate health care for specific clinical circumstances.”* (Institute of Medicine, 1990) | | |

## 2.3 Supplementary Table 3. *List of Elements for Data Extraction*

1. Title

2. Authors

3. Publication year

4. Journal

5. DOI

6. Reference

7. Access

8. Geographical coverage of the study, where applicable

9. Study status (completed, on-going)

10. A: Type of research (Primary, Secondary)

11. B: Type of research (Quantitative, Qualitative, Mixed-method)

12. Study type (Impact evaluation, Published study protocol, Systematic review, Meta-analysis, Systematic review with meta-analysis, Clinical trial registry, Clinical practice guideline)

13. Study design

14. Sample: Size (N)

15. Sample: Sex

16. Sample: Age (Mean)

17. Sample: Age (Min.)

18. Sample: Age (Max.)

19. A: Sample: LGBTQ2IA+ reported (Y/N)

20. B: Sample: LGBTQ2IA+ (N)

21. A: Sample: Race/ethnicity reported (Y/N)

22. B: Sample: BIPOC participants (N)

23. Type of ABI

24. Target MHSU problem(s) (DSM category)

25. Was the intervention multi-component?

26. Intervention Category(s)

27. Outcome Category(s)

*Note.* Acquired brain injury (ABI), mental health/substance use (MHSU).

**Supplementary Table 4.**

*Summary of Included Records*

| **First Author (Year)** | **Publication Type** | **Country** | **Study Design** | **Type of ABI** | **Intervention Category(s)** | **Outcome Category(s)** |
| --- | --- | --- | --- | --- | --- | --- |
| Aaronson (2022) | Clinical trial registry | United States of America | RCTs/experimental | mTBI or concussion | Neuromodulation and neurofeedback | Suicide and self-harm |
| Aboulafia-brakha (2016) | Impact evaluation | Switzerland | Lower-tier quasi-experimental designs | ABI (non-specific) | Psychotherapy (out-patient) | Aggression |
| Acabchuk (2021) | Systematic review with meta-analysis | Multiple Countries | Systematic review with meta-analysis | mTBI or concussion | Mindfulness and meditation | Depression; anxiety; quality of life |
| Ackland (2019) | Systematic review | Multiple Countries | Systematic review | mTBI or concussion | Psychotherapy (out-patient) | Trauma and stress related |
| Agnoli (2024) | Impact evaluation | United States of America | RCTs/experimental | mTBI or concussion | Tele-health and technology-based | Depression; trauma and stress related |
| Ahl (2017) | Impact evaluation | Sweden | Upper-tier quasi-experimental designs | Severe TBI | Non-psychotropic medications | Depression |
| Ahrens (2022) | Systematic review with meta-analysis | Multiple Countries | Systematic review with meta-analysis | Stroke | Psychotherapy (out-patient) | Depression; anxiety |
| Ahrens (2023) | Systematic review | Canada | Systematic review with meta-analysis | Stroke | Psychotherapy (out-patient) | Depression; anxiety |
| Ai (2023) | Impact evaluation | China | Lower-tier quasi-experimental designs | Stroke | Herbal medicine | Depression; anxiety |
| Aidar (2018) | Impact evaluation | Brazil | RCTs/experimental | Stroke | Exercise and movement-based | Physical activity; depression; anxiety |
| Albrecht (2020) | Impact evaluation | United States of America | Non-experimental designs | TBI (non-specific) | Antidepressants | Addiction-related; sleep and eating; anxiety |
| Allen (2014) | Impact evaluation | Canada | RCTs/experimental | Stroke | Home-based care | Depression; anxiety |
| Allida (2019) | Systematic review with meta-analysis | Multiple Countries | Systematic review with meta-analysis | Stroke | Antidepressants | Self-awareness and self-efficacy |
| Allida (2020) | Systematic review with meta-analysis | United States of America | Systematic review with meta-analysis | Stroke | Antidepressants; neuromodulation and neurofeedback; psychotherapy (out-patient) | Depression |
| Allida (2020) | Systematic review | Multiple Countries | Systematic review | Stroke | Antidepressants; psychotherapy (out-patient); neuromodulation and neurofeedback | Depression |
| Allida (2023) | Systematic review | United States of America | Systematic review with meta-analysis | Stroke | Antidepressants; neuromodulation and neurofeedback; psychotherapy (out-patient) | Depression |
| Almeida (2021) | Impact evaluation | Australia | Upper-tier quasi-experimental designs | Stroke | Antidepressants | Depression |
| Alwledat (2023) | Impact evaluation | Jordan | Upper-tier quasi-experimental designs | Stroke | Music and art | Depression; anxiety |
| An (2017) | Impact evaluation | South Korea | RCTs/experimental | Stroke | Neuromodulation and neurofeedback | Depression; quality of life |
| Ansari (2014) | Impact evaluation | India | RCTs/experimental | TBI (non-specific) | Non-psychotropic medications | Depression |
| Arcadi (2021) | Impact evaluation | Italy | Non-experimental designs | Stroke | Non-psychotropic medications | Depression |
| Ashman (2014) | Impact evaluation | United States of America | RCTs/experimental | TBI (non-specific) | Psychotherapy (out-patient) | Depression; anxiety |
| Ashworth (2015) | Impact evaluation | United Kingdom | Non-experimental designs | ABI (non-specific) | Psychotherapy (out-patient) | Anxiety |
| Aslan (2022) | Impact evaluation | Turkey | RCTs/experimental | Stroke | Acupuncture and massage; herbal medicine | Depression; sleep and eating |
| Baek (2014) | Impact evaluation | Korea | RCTs/experimental | Stroke | Exercise and movement-based | Depression |
| Baig (2019) | Impact evaluation | United States of America | RCTs/experimental | mTBI or concussion | Integrated care and case management | Healthcare utilization |
| Baig (2020) | Impact evaluation | United States of America | RCTs/experimental | mTBI or concussion | Antipsychotics and anticonvulsants | Trauma and stress related |
| Baker (2018) | Systematic review | Multiple Countries | Systematic review | Stroke | Psychotherapy (out-patient) | Depression |
| Baldo (2019) | Clinical trial registry | United States of America | RCTs/experimental | Stroke | Mindfulness and meditation | Anxiety; cognition |
| Balea (2021) | Impact evaluation | Romania | RCTs/experimental | Stroke | Non-psychotropic medications | Depression |
| Balram (2024) | Systematic review | Australia | Systematic review with meta-analysis | Stroke | Cognitive training/rehabilitation; psychotherapy (out-patient); tele-health and technology-based | Depression; anxiety |
| Bang-jiang (2018) | Systematic review with meta-analysis | Multiple Countries | Systematic review with meta-analysis | Stroke | Acupuncture and massage | Depression |
| Bao (2024) | Impact evaluation | China | RCTs/experimental | Stroke | Long-term in-patient services | Quality of life |
| Barua (2024) | Meta analysis | Canada | Systematic review with meta-analysis | TBI (non-specific) | Psychotherapy (out-patient) | Depression; anxiety |
| Batki (2016) | Clinical trial registry | United States of America | RCTs/experimental | mTBI or concussion | Non-psychotropic medications | Addiction-related |
| Bay (2019) | Impact evaluation | United States of America | Lower-tier quasi-experimental designs | TBI (non-specific) | Psychotherapy (out-patient) | Depression |
| Baylan (2016) | Systematic review | Multiple Countries | Systematic review | Stroke | Music and art | Cognition; depression |
| Bedard (2014) | Impact evaluation | Canada | RCTs/experimental | TBI (non-specific) | Mindfulness and meditation | Depression |
| Beedham (2020) | Systematic review with meta-analysis | Multiple Countries | Systematic review with meta-analysis | TBI (non-specific) | Antidepressants; psychostimulants; neuromodulation and neurofeedback | Depression |
| Belanger (2022) | Impact evaluation | United States of America | RCTs/experimental | mTBI or concussion | Tele-health and technology-based | Depression; trauma and stress related; self-awareness and self-efficacy |
| Bell (2017) | Impact evaluation | United States of America | RCTs/experimental | mTBI or concussion | Tele-health and technology-based; cognitive training/rehabilitation | Somatic symptoms; depression |
| Bellon (2015) | Impact evaluation | United States of America | Lower-tier quasi-experimental designs | TBI (non-specific) | Exercise and movement-based | Depression; trauma and stress related |
| Beresford (2022) | Impact evaluation | United States of America | RCTs/experimental | mTBI or concussion | Antipsychotics and anticonvulsants | Aggression |
| Bermudo-gallaguet (2022) | Published study protocol | Spain | RCTs/experimental | Stroke | Mindfulness and meditation; exercise and movement-based | Depression; anxiety; trauma and stress related |
| Berthier (2022) | Impact evaluation | Spain | Upper-tier quasi-experimental designs | Stroke | Psychotherapy (in-patient) | Depression |
| Berthier (2023) | Impact evaluation | Spain | RCTs/experimental | Stroke | Psychostimulants; cognitive training/rehabilitation | Depression |
| Berthier (2023) | Impact evaluation | Spain | RCTs/experimental | Stroke | Psychostimulants; social support programs | Depression |
| Bhardwaj (2018) | Impact evaluation | India | RCTs/experimental | Stroke | Neuromodulation and neurofeedback; psychotherapy (in-patient) | Depression; anxiety |
| Bidzan (2020) | Impact evaluation | Poland | Non-experimental designs | ABI (non-specific) | Antipsychotics and anticonvulsants | Aggression |
| Bilodeau (2021) | Impact evaluation | Canada | Non-experimental designs | TBI (non-specific) | Non-psychotropic medications | Aggression |
| Blazquez-gonzalez (2024) | Systematic review with meta-analysis | Spain | Systematic review with meta-analysis | Stroke | Tele-health and technology-based | Depression |
| Blazquez-gonzalez (2024) | Impact evaluation | Spain | RCTs/experimental | Stroke | Tele-health and technology-based | Anxiety; depression |
| Boglo (2023) | Impact evaluation | United Kingdom | Non-experimental designs | TBI (non-specific) | Psychotherapy (in-patient) | Anxiety |
| Borg (2020) | Impact evaluation | Australia | RCTs/experimental | ABI (non-specific) | Integrated care and case management | Depression; anxiety |
| Borgen (2023) | Impact evaluation | Norway | RCTs/experimental | TBI (non-specific) | Home-based care | Quality of life |
| Bragstad (2020) | Impact evaluation | Norway | RCTs/experimental | Stroke | Psychotherapy (out-patient) | Quality of life |
| Brenner (2018) | Impact evaluation | United States of America | RCTs/experimental | Moderate TBI; severe TBI | Psychotherapy (out-patient) | Depression |
| Brody (2020) | Clinical trial registry | United States of America | RCTs/experimental | mTBI or concussion | Psychotherapy (out-patient); tele-health and technology-based | Sleep and eating |
| Brody (2022) | Clinical trial registry | United States of America | RCTs/experimental | mTBI or concussion | Neuromodulation and neurofeedback | Depression |
| Brown (2019) | Impact evaluation | United States of America | RCTs/experimental | TBI (non-specific) | Neuromodulation and neurofeedback | Depression |
| Bucur (2018) | Systematic review | Multiple Countries | Systematic review | Stroke | Neuromodulation and neurofeedback | Depression |
| Byrne (2016) | Systematic review with meta-analysis | Multiple Countries | Systematic review with meta-analysis | ABI (non-specific) | Psychotherapy (out-patient) | Aggression |
| Cai (2021) | Systematic review with meta-analysis | Multiple Countries | Systematic review with meta-analysis | Stroke | Herbal medicine | Depression |
| Cai (2022) | Impact evaluation | China | RCTs/experimental | Stroke | Acupuncture and massage | Depression |
| Cao (2020) | Impact evaluation | China | RCTs/experimental | Stroke | Antidepressants | Depression |
| Carrick (2015) | Impact evaluation | United States of America | Lower-tier quasi-experimental designs | TBI (non-specific) | Tele-health and technology-based | Trauma and stress related |
| Carvalho (2020) | Clinical trial registry | Brazil | RCTs/experimental | TBI (non-specific) | Neuromodulation and neurofeedback | Anxiety; depression |
| Chalmers (2019) | Impact evaluation | New Zealand | Upper-tier quasi-experimental designs | Stroke | Psychotherapy (out-patient) | Depression; anxiety; quality of life |
| Chan (2021) | Published study protocol | China | RCTs/experimental | Stroke | Music and art | Depression; quality of life |
| Chan (2021) | Impact evaluation | China | RCTs/experimental | Stroke | Music and art | Depression |
| Chan (2022) | Systematic review | Multiple Countries | Systematic review | TBI (non-specific) | Integrated care and case management | Depression; trauma and stress related |
| Chan (2023) | Systematic review with meta-analysis | China | Systematic review with meta-analysis | Stroke | Music and art | Depression |
| Chang (2020) | Impact evaluation | Taiwan | RCTs/experimental | Stroke | Neuromodulation and neurofeedback | Somatic symptoms |
| Chang (2023) | Meta analysis | China | Systematic review with meta-analysis | TBI (non-specific) | Neuromodulation and neurofeedback | Depression |
| Chen (2020) | Systematic review with meta-analysis | Multiple Countries | Systematic review with meta-analysis | mTBI or concussion | Psychotherapy (out-patient) | Cognition |
| Chen (2020) | Impact evaluation | China | RCTs/experimental | Stroke | Acute in-patient services | Depression |
| Chen (2020) | Systematic review | Multiple Countries | Systematic review | Stroke | Integrated care and case management | Depression; anxiety |
| Chen (2022) | Published study protocol | China | RCTs/experimental | Stroke | Acupuncture and massage | Depression |
| Chen (2022) | Published study protocol | China | RCTs/experimental | Stroke | Acute in-patient services | Depression |
| Chen (2023) | Impact evaluation | Taiwan | RCTs/experimental | TBI (non-specific) | Acupuncture and massage | Sleep and eating |
| Chen (2024) | Systematic review with meta-analysis | China | Systematic review with meta-analysis | Stroke | Exercise and movement-based | Depression |
| Chen (2024) | Systematic review with meta-analysis | China | Systematic review with meta-analysis | Stroke | Antidepressants | Depression |
| Cheng (2018) | Clinical trial registry | China | RCTs/experimental | Stroke | Patient-caregiver interventions | Depression |
| Cheng (2018) | Impact evaluation | China | RCTs/experimental | Stroke | Cognitive training/rehabilitation | Cognition; anxiety; depression |
| Cheng (2021) | Systematic review with meta-analysis | Multiple Countries | Systematic review with meta-analysis | TBI (non-specific) | Acute in-patient services; psychotherapy (in-patient) | Depression |
| Cheng (2021) | Impact evaluation | China | RCTs/experimental | Stroke | Mindfulness and meditation | Depression |
| Chinchai (2023) | Impact evaluation | Thailand | Upper-tier quasi-experimental designs | Stroke | Music and art | Somatic symptoms; depression |
| Chippala (2020) | Impact evaluation | India | RCTs/experimental | Stroke | Acute in-patient services; exercise and movement-based | Depression; anxiety |
| Chiu (2022) | Clinical trial registry | Taiwan | RCTs/experimental | TBI (non-specific) | Psychotherapy (out-patient) | Sleep and eating |
| Choi (2022) | Impact evaluation | South Korea | RCTs/experimental | Stroke | Psychotherapy (in-patient) | Depression; anxiety |
| Choi (2024) | Impact evaluation | Korea | Lower-tier quasi-experimental designs | Stroke | Acute in-patient services | Depression; sleep and eating |
| Choo (2022) | Systematic review with meta-analysis | Multiple Countries | Systematic review with meta-analysis | Stroke | Patient-caregiver interventions | Anxiety; depression |
| Chou (2024) | Impact evaluation | Taiwan | RCTs/experimental | Stroke | Music and art | Depression |
| Chow ow (2023) | Impact evaluation | China | RCTs/experimental | Stroke | Psychotherapy (out-patient) | Depression |
| Chu (2023) | Systematic review | United Kingdom | Systematic review with meta-analysis | Stroke | Herbal medicine; antidepressants | Sleep and eating |
| Chun (2018) | Published study protocol | United Kingdom | RCTs/experimental | Stroke | Tele-health and technology-based | Anxiety |
| Chun (2018) | Systematic review | Multiple Countries | Systematic review | Stroke | Psychotherapy (out-patient); antidepressants; anxiolytics | Anxiety |
| Chun (2020) | Published study protocol | United Kingdom | RCTs/experimental | Stroke | Psychotherapy (out-patient); mindfulness and meditation | Anxiety |
| Clay (2019) | Systematic review | Multiple Countries | Systematic review | TBI (non-specific) | Antidepressants; non-psychotropic medications | Depression |
| Cui (2018) | Systematic review with meta-analysis | Multiple Countries | Systematic review with meta-analysis | Stroke | Antidepressants | Depression |
| Cui (2023) | Impact evaluation | China | RCTs/experimental | Stroke | Antidepressants; acupuncture and massage | Depression |
| Cullen (2018) | Published study protocol | Scotland | RCTs/experimental | ABI (non-specific) | Psychotherapy (in-patient) | Depression; quality of life |
| D'anci (2019) | Systematic review | Multiple Countries | Systematic review | Stroke | Exercise and movement-based | Physical activity; quality of life |
| Dasilvajunior (2019) | Impact evaluation | Brazil | Non-experimental designs | Stroke | Neuromodulation and neurofeedback | Depression |
| Dayuan (2022) | Systematic review with meta-analysis | Multiple Countries | Systematic review with meta-analysis | Stroke | Music and art | Depression |
| Deb (2018) | Published study protocol | United Kingdom | RCTs/experimental | TBI (non-specific) | Antipsychotics and anticonvulsants | Aggression |
| Degraba (2021) | Impact evaluation | United States of America | Lower-tier quasi-experimental designs | TBI (non-specific) | Integrated care and case management | Trauma and stress related; anxiety |
| Dehvan (2018) | Clinical trial registry | Iran | RCTs/experimental | Stroke | Acupuncture and massage | Anxiety |
| Deng (2017) | Systematic review with meta-analysis | Multiple Countries | Systematic review with meta-analysis | Stroke | Antidepressants | Depression |
| Deng (2018) | Systematic review with meta-analysis | Multiple Countries | Systematic review with meta-analysis | Stroke | Antidepressants | Depression |
| Dewi (2020) | Impact evaluation | Indonesia | Upper-tier quasi-experimental designs | Stroke | Psychotherapy (in-patient) | Quality of life |
| Dickey (2021) | Clinical trial registry | Canada | RCTs/experimental | mTBI or concussion | Mindfulness and meditation; tele-health and technology-based | Depression; anxiety |
| Dindo (2019) | Clinical trial registry | United States of America | RCTs/experimental | TBI (non-specific) | Psychotherapy (out-patient) | Anxiety; depression; community engagement |
| Dindo (2020) | Impact evaluation | United States of America | RCTs/experimental | TBI (non-specific) | Psychotherapy (out-patient) | Trauma and stress related |
| Ding (2021) | Published study protocol | China | RCTs/experimental | Stroke | Herbal medicine | Depression |
| Ding (2024) | Impact evaluation | China | Non-experimental designs | Stroke | Tele-health and technology-based | Anxiety; depression |
| Ding (2024) | Impact evaluation | China | RCTs/experimental | Stroke | Long-term in-patient services | Quality of life; depression |
| Dolbow (2020) | Systematic review | Multiple Countries | Systematic review | TBI (non-specific) | Exercise and movement-based | Depression |
| Donnelly (2024) | Impact evaluation | United States of America | RCTs/experimental | TBI (non-specific) | Patient-caregiver interventions; mindfulness and meditation | Quality of life |
| Doshi (2019) | Clinical trial registry | Singapore | RCTs/experimental | Stroke | Mindfulness and meditation; home-based care | Depression; anxiety |
| Duan (2023) | Impact evaluation | China | RCTs/experimental | Stroke | Neuromodulation and neurofeedback; mindfulness and meditation | Depression |
| Eagle (2021) | Impact evaluation | United States of America | Upper-tier quasi-experimental designs | TBI (non-specific) | Psychotherapy (out-patient); exercise and movement-based | Depression |
| Elbogen (2019) | Impact evaluation | United States of America | RCTs/experimental | TBI (non-specific) | Patient-caregiver interventions ; tele-health and technology-based; cognitive training/rehabilitation | Trauma and stress related; cognition |
| Elbogen (2021) | Impact evaluation | United States of America | Non-experimental designs | TBI (non-specific) | Neuromodulation and neurofeedback | Somatic symptoms; trauma and stress related |
| Ellis-hill (2019) | Impact evaluation | United Kingdom | RCTs/experimental | Stroke | Music and art | Depression; self-awareness and self-efficacy |
| Eng (2014) | Systematic review with meta-analysis | Canada | Systematic review with meta-analysis | Stroke | Exercise and movement-based | Depression |
| Erickson (2024) | Impact evaluation | Usa | RCTs/experimental | TBI (non-specific) | Psychotherapy (out-patient) | Depression |
| Evans (2018) | Clinical trial registry | United States of America | RCTs/experimental | Stroke | Psychotherapy (out-patient) | Depression; anxiety; community engagement |
| Evans-hudnall (2014) | Impact evaluation | United States of America | RCTs/experimental | Stroke | Social support programs | Addiction-related; anxiety |
| Fakes (2024) | Impact evaluation | Australia | RCTs/experimental | Stroke | Tele-health and technology-based | Depression; quality of life |
| Fang (2017) | Impact evaluation | China | RCTs/experimental | Stroke | Psychotherapy (in-patient) | Depression; anxiety |
| Fann (2015) | Impact evaluation | United States of America | RCTs/experimental | TBI (non-specific) | Tele-health and technology-based; psychotherapy (in-patient) | Depression |
| Fann (2017) | Impact evaluation | United States of America | RCTs/experimental | TBI (non-specific) | Antidepressants | Depression |
| Feng (2018) | Meta analysis | Multiple Countries | Meta analysis | Stroke | Antidepressants | Depression |
| Feng (2022) | Systematic review with meta-analysis | Multiple Countries | Systematic review with meta-analysis | Stroke | Antidepressants | Depression |
| Feng (2024) | Impact evaluation | China | RCTs/experimental | Stroke | Non-psychotropic medications | Depression |
| Fleming (2024) | Impact evaluation | United Kingdom | RCTs/experimental | Stroke | Tele-health and technology-based | Sleep and eating |
| Galimberti (2024) | Systematic review with meta-analysis | Austria | Systematic review with meta-analysis | TBI (non-specific) | Neuromodulation and neurofeedback | Depression; anxiety; cognition |
| Gao (2017) | Impact evaluation | China | RCTs/experimental | Stroke | Psychotherapy (out-patient); antidepressants | Depression |
| Gao (2019) | Systematic review with meta-analysis | Multiple Countries | Systematic review with meta-analysis | TBI (non-specific) | Antidepressants | Depression |
| Gao (2021) | Clinical trial registry | China | RCTs/experimental | Stroke | Integrated care and case management | Anxiety |
| Gao (2022) | Impact evaluation | China | RCTs/experimental | Stroke | Acute in-patient services | Depression |
| Gao (2023) | Impact evaluation | China | RCTs/experimental | Stroke | Acute in-patient services | Quality of life |
| Gertler (2015) | Systematic review | Multiple Countries | Systematic review | TBI (non-specific) | Psychotherapy (out-patient) | Depression |
| Gezginyazici (2024) | Impact evaluation | Turkey | RCTs/experimental | Stroke | Music and art | Depression |
| Ghaffari (2022) | Impact evaluation | Iran | RCTs/experimental | Stroke | Exercise and movement-based | Sleep and eating; depression; activities of daily living/life skills |
| Gladwyn-khan (2023) | Impact evaluation | United Kingdom | RCTs/experimental | Stroke | Psychotherapy (out-patient) | Quality of life |
| Glinac (2024) | Impact evaluation | Bosnia and Herzegovina | RCTs/experimental | Stroke | Exercise and movement-based; integrated care and case management | Depression |
| Glintborg (2016) | Impact evaluation | Denmark | Non-experimental designs | ABI (non-specific) | Integrated care and case management | Quality of life; depression; community engagement |
| Golding (2016) | Impact evaluation | United Kingdom | RCTs/experimental | Stroke | Mindfulness and meditation | Anxiety |
| Golding (2017) | Impact evaluation | United Kingdom | RCTs/experimental | Stroke | Mindfulness and meditation | Anxiety; depression |
| Golding (2018) | Impact evaluation | United Kingdom | RCTs/experimental | Stroke | Mindfulness and meditation | Depression |
| González (2021) | Clinical trial registry | Spain | RCTs/experimental | Stroke | Tele-health and technology-based | Anxiety; depression |
| Grabowska-fudala (2018) | Impact evaluation | Poland | Upper-tier quasi-experimental designs | Stroke | Acute in-patient services | Depression |
| Graven (2016) | Impact evaluation | United States of America | RCTs/experimental | Stroke | Home-based care | Depression |
| Gregory (2019) | Clinical trial registry | United States of America | RCTs/experimental | Stroke | Exercise and movement-based | Depression; physical activity |
| Griffin-musick (2021) | Impact evaluation | United States of America | Lower-tier quasi-experimental designs | Stroke | Cognitive training/rehabilitation | Depression |
| Gros (2017) | Impact evaluation | United States of America | RCTs/experimental | TBI (non-specific) | Psychotherapy (in-patient) | Trauma and stress related; addiction-related |
| Gu (2017) | Impact evaluation | Republic of Korea | RCTs/experimental | Stroke | Neuromodulation and neurofeedback | Depression |
| Gu (2018) | Impact evaluation | China | RCTs/experimental | Stroke | Acute in-patient services | Quality of life; anxiety; depression |
| Gu (2020) | Meta analysis | Multiple Countries | Meta analysis | Stroke | Antidepressants | Depression |
| Guillaumier (2019) | Published study protocol | Australia | RCTs/experimental | Stroke | Tele-health and technology-based | Quality of life |
| Guo (2022) | Systematic review with meta-analysis | Multiple Countries | Systematic review with meta-analysis | Stroke | Acupuncture and massage | Depression |
| Guo (2023) | Impact evaluation | China | RCTs/experimental | Stroke | Acute in-patient services | Depression |
| Hadidi (2015) | Impact evaluation | United States of America | RCTs/experimental | Stroke | Psychotherapy (out-patient) | Depression |
| Haire (2021) | Impact evaluation | Canada | RCTs/experimental | Stroke | Music and art | Depression; cognition |
| Hammond (2014) | Impact evaluation | United States of America | RCTs/experimental | TBI (non-specific) | Non-psychotropic medications | Aggression |
| Hammond (2015) | Impact evaluation | United States of America | RCTs/experimental | TBI (non-specific) | Non-psychotropic medications | Aggression |
| Hammond (2018) | Impact evaluation | United States of America | Non-experimental designs | TBI (non-specific) | Non-psychotropic medications | Somatic symptoms; self-awareness and self-efficacy |
| Han (2018) | Impact evaluation | United States of America | RCTs/experimental | TBI (non-specific) | Cognitive training/rehabilitation | Depression |
| Han (2023) | Systematic review with meta-analysis | Multiple Countries | Systematic review with meta-analysis | Stroke | Mindfulness and meditation | Depression |
| Han (2024) | Impact evaluation | China | Lower-tier quasi-experimental designs | Stroke | Neuromodulation and neurofeedback; acupuncture and massage | Depression |
| Handayani (2020) | Impact evaluation | Indonesia | Upper-tier quasi-experimental designs | Stroke | Social support programs | Depression |
| Hang (2021) | Meta analysis | Multiple Countries | Meta analysis | Stroke | Acupuncture and massage | Depression |
| Hao (2023) | Systematic review | Multiple Countries | Systematic review | Stroke | Neuromodulation and neurofeedback | Depression |
| Harch (2017) | Impact evaluation | United States of America | RCTs/experimental | mTBI or concussion | Acute in-patient services | Trauma and stress related; depression |
| Hart (2019) | Published study protocol | United States of America | RCTs/experimental | TBI (non-specific) | Tele-health and technology-based | Depression |
| Hart (2020) | Impact evaluation | United States of America | RCTs/experimental | TBI (non-specific) | Tele-health and technology-based | Depression; community engagement |
| Henderson (2017) | Impact evaluation | United States of America | RCTs/experimental | TBI (non-specific) | Neuromodulation and neurofeedback | Depression |
| Herrold (2019) | Clinical trial registry | United States of America | RCTs/experimental | mTBI or concussion | Neuromodulation and neurofeedback | Quality of life |
| Hicks (2020) | Systematic review | Multiple Countries | Systematic review | TBI (non-specific) | Non-psychotropic medications | Somatic symptoms; self-awareness and self-efficacy |
| Hicks (2021) | Systematic review | Multiple Countries | Systematic review | TBI (non-specific) | Antidepressants | Anxiety |
| Hill (2019) | Published study protocol | United Kingdom | RCTs/experimental | Stroke | Psychotherapy (out-patient); home-based care | Depression; anxiety; activities of daily living/life skills |
| Hoffmann (2015) | Published study protocol | Australia | RCTs/experimental | Stroke | Psychotherapy (in-patient) | Depression; anxiety |
| Holleman (2018) | Impact evaluation | Netherlands | Upper-tier quasi-experimental designs | ABI (non-specific) | Psychotherapy (out-patient) | Quality of life; depression; anxiety |
| Hong (2021) | Impact evaluation | China | RCTs/experimental | ABI (non-specific) | Acute in-patient services | Depression; quality of life |
| Hordacre (2021) | Impact evaluation | Australia | RCTs/experimental | Stroke | Neuromodulation and neurofeedback | Depression |
| Hoy (2019) | Impact evaluation | Australia | RCTs/experimental | TBI (non-specific) | Neuromodulation and neurofeedback | Depression |
| Hsin-tung (2022) | Clinical trial registry | Taiwan | RCTs/experimental | Stroke | Acupuncture and massage | Depression |
| Hu (2015) | Impact evaluation | China | RCTs/experimental | Stroke | Non-psychotropic medications | Depression |
| Huang (2017) | Meta analysis | Multiple Countries | Meta analysis | Stroke | Home-based care | Depression |
| Huang (2021) | Published study protocol | China | RCTs/experimental | Stroke | Acupuncture and massage; herbal medicine | Sleep and eating; anxiety |
| Hung (2019) | Systematic review with meta-analysis | Multiple Countries | Systematic review with meta-analysis | Stroke | Acupuncture and massage | Cognition; depression |
| Hwang (2021) | Systematic review | Multiple Countries | Systematic review | Stroke | Tele-health and technology-based | Self-awareness and self-efficacy |
| Jackson (2019) | Impact evaluation | United States of America | Non-experimental designs | TBI (non-specific) | Psychotherapy (out-patient) | Trauma and stress related |
| Jak (2015) | Published study protocol | United States of America | RCTs/experimental | TBI (non-specific) | Psychotherapy (out-patient) | Trauma and stress related |
| Jak (2019) | Impact evaluation | United States of America | RCTs/experimental | mTBI or concussion | Psychotherapy (out-patient) | Trauma and stress related; cognition |
| Janak (2017) | Impact evaluation | United States of America | RCTs/experimental | mTBI or concussion | Integrated care and case management; psychotherapy (out-patient) | Somatic symptoms; trauma and stress related |
| Jani (2018) | Impact evaluation | United Kingdom | RCTs/experimental | Stroke | Mindfulness and meditation | Depression |
| Jennings (2023) | Systematic review | Australia | Systematic review with meta-analysis | mTBI or concussion | Integrated care and case management | Somatic symptoms |
| Jiali (2018) | Clinical trial registry | China | RCTs/experimental | Stroke | Neuromodulation and neurofeedback | Depression |
| Jiancheng (2021) | Clinical trial registry | China | RCTs/experimental | Stroke | Neuromodulation and neurofeedback | Depression |
| Jiang (2022) | Impact evaluation | China | RCTs/experimental | Stroke | Psychotherapy (in-patient) | Trauma and stress related |
| Jiang (2023) | Impact evaluation | China | RCTs/experimental | Stroke | Neuromodulation and neurofeedback | Trauma and stress related |
| Jiang (2023) | Systematic review with meta-analysis | China | Systematic review with meta-analysis | Stroke | Acupuncture and massage | Depression |
| Jie (2023) | Impact evaluation | China | RCTs/experimental | Stroke | Cognitive training/rehabilitation; acute in-patient services | Cognition; depression |
| Jin (2018) | Meta analysis | Multiple Countries | Meta analysis | Stroke | Herbal medicine | Depression |
| Jin (2024) | Impact evaluation | China | RCTs/experimental | Stroke | Antidepressants | Depression |
| Johansson (2014) | Impact evaluation | Sweden | RCTs/experimental | TBI (non-specific) | Psychostimulants | Cognition |
| Johansson (2017) | Impact evaluation | Sweden | Upper-tier quasi-experimental designs | mTBI or concussion | Psychostimulants | Sleep and eating; depression; anxiety |
| Jorge (2016) | Impact evaluation | United States of America | RCTs/experimental | TBI (non-specific) | Antidepressants | Depression |
| Junttola (2024) | Impact evaluation | Finland | Non-experimental designs | Stroke | Acute in-patient services | Quality of life |
| Jurick (2020) | Impact evaluation | United States of America | RCTs/experimental | mTBI or concussion | Psychotherapy (in-patient) | Cognition; trauma and stress related |
| Kalbouneh (2022) | Systematic review with meta-analysis | Multiple Countries | Systematic review with meta-analysis | Stroke | Antidepressants | Depression; anxiety |
| Kang (2022) | Impact evaluation | China | RCTs/experimental | Stroke | Tele-health and technology-based | Depression |
| KeeSystematic review with meta-analysis (2022) | Clinical trial registry | United States of America | RCTs/experimental | Stroke | Tele-health and technology-based | Cognition |
| Kennedy (2022) | Impact evaluation | United States of America | Non-experimental designs | mTBI or concussion | Cognitive training/rehabilitation | Depression; somatic symptoms |
| Kerr (2018) | Impact evaluation | Australia | RCTs/experimental | Stroke | Psychotherapy (out-patient) | Depression |
| Kim (2017) | Impact evaluation | South Korea | RCTs/experimental | Stroke | Neuromodulation and neurofeedback | Depression; quality of life; somatic symptoms |
| Kim (2017) | Impact evaluation | Republic of Korea | RCTs/experimental | Stroke | Antidepressants | Depression |
| Kim (2020) | Impact evaluation | South Korea | Upper-tier quasi-experimental designs | Stroke | Tele-health and technology-based | Depression; quality of life |
| Kim (2022) | Impact evaluation | South Korea | RCTs/experimental | Stroke | Tele-health and technology-based | Sleep and eating |
| King (2023) | Impact evaluation | United States of America | Non-experimental designs | mTBI or concussion | Cognitive training/rehabilitation | Depression; trauma and stress related |
| Kiper (2022) | Impact evaluation | Poland | RCTs/experimental | Stroke | Tele-health and technology-based | Depression |
| Kirkevold (2018) | Published study protocol | Norway | RCTs/experimental | Stroke | Psychotherapy (out-patient) | Quality of life |
| Kirkness (2017) | Impact evaluation | United States of America | RCTs/experimental | Stroke | Psychotherapy (out-patient) | Depression |
| Kitzmuller (2019) | Impact evaluation | Norway | Non-experimental designs | Stroke | Psychotherapy (out-patient) | Quality of life |
| Klyce (2018) | Impact evaluation | United States of America | RCTs/experimental | TBI (non-specific) | Withdrawal management centres | Activities of daily living/life skills |
| Knapp (2017) | Systematic review | Multiple Countries | Systematic review | Stroke | Anxiolytics; mindfulness and meditation; antidepressants | Anxiety |
| Komber (2024) | Systematic review with meta-analysis | United Kingdom | Systematic review with meta-analysis | Stroke | Exercise and movement-based; psychotherapy (out-patient); neuromodulation and neurofeedback | Sleep and eating |
| Kongkasuwan (2016) | Impact evaluation | Thailand | RCTs/experimental | Stroke | Music and art; mindfulness and meditation | Cognition; anxiety; depression |
| Konigsberg (2021) | Impact evaluation | Germany | RCTs/experimental | Stroke | Acute in-patient services | Depression |
| Kootker (2016) | Impact evaluation | Netherlands | RCTs/experimental | Stroke | Psychotherapy (out-patient) | Depression |
| Kotov (2020) | Published study protocol | Russia | RCTs/experimental | Stroke | Exercise and movement-based | Anxiety |
| Kreitzer (2019) | Meta analysis | Multiple Countries | Meta analysis | TBI (non-specific) | Antidepressants | Depression |
| Krese (2020) | Impact evaluation | United States of America | RCTs/experimental | TBI (non-specific) | Exercise and movement-based | Sleep and eating; anxiety |
| Kreutzer (2018) | Impact evaluation | United States of America | RCTs/experimental | TBI (non-specific) | Social support programs | Self-awareness and self-efficacy |
| Kristofersson (2016) | Impact evaluation | United States of America | Non-experimental designs | TBI (non-specific) | Mindfulness and meditation | Anxiety |
| Kucukakgun (2022) | Clinical trial registry | Turkey | RCTs/experimental | Stroke | Social support programs | Cognition; sleep and eating; depression |
| Kuppelin (2024) | Impact evaluation | France | Lower-tier quasi-experimental designs | ABI (non-specific) | Psychotherapy (out-patient) | Quality of life; depression |
| Kusec (2020) | Published study protocol | United Kingdom | RCTs/experimental | ABI (non-specific) | Social support programs | Depression |
| Kwon (2019) | Systematic review with meta-analysis | Multiple Countries | Systematic review with meta-analysis | mTBI or concussion | Neuromodulation and neurofeedback | Depression |
| Kwon (2019) | Systematic review with meta-analysis | Multiple Countries | Systematic review with meta-analysis | Stroke | Herbal medicine | Depression |
| Kwon (2021) | Impact evaluation | South Korea | RCTs/experimental | Stroke | Antidepressants | Depression |
| Künzi (2018) | Impact evaluation | Switzerland | RCTs/experimental | ABI (non-specific) | Psychotherapy (in-patient) | Anxiety |
| Lai (2022) | Impact evaluation | Taiwan | RCTs/experimental | Stroke | Exercise and movement-based | Depression |
| Lamching (2023) | Systematic review with meta-analysis | China | Systematic review with meta-analysis | Stroke | Acupuncture and massage | Depression |
| Lan (2024) | Impact evaluation | China | RCTs/experimental | Stroke | Acupuncture and massage | Depression |
| Lanctot (2020) | Clinical practice guideline | Canada | Clinical practice guideline | Stroke | Integrated care and case management | Cognition; sleep and eating; depression |
| Large (2020) | Impact evaluation | United Kingdom | Lower-tier quasi-experimental designs | Stroke | Psychotherapy (in-patient) | Healthcare utilization |
| Lavu (2022) | Systematic review | Multiple Countries | Systematic review | Stroke | Antidepressants | Depression |
| Lawrence (2017) | Impact evaluation | United States of America | Lower-tier quasi-experimental designs | TBI (non-specific) | Social support programs | Activities of daily living/life skills; self-awareness and self-efficacy |
| Le (2024) | Systematic review | Japan | Systematic review with meta-analysis | Stroke | Psychotherapy (in-patient) | Quality of life |
| Ledanseur (2019) | Impact evaluation | United States of America | RCTs/experimental | Stroke | Music and art | Anxiety; depression |
| Lee (2014) | Impact evaluation | United States of America | RCTs/experimental | TBI (non-specific) | Exercise and movement-based; cognitive training/rehabilitation | Depression; quality of life |
| Lee (2016) | Impact evaluation | South Korea | Lower-tier quasi-experimental designs | Stroke | Psychotherapy (out-patient) | Addiction-related |
| Lee (2018) | Impact evaluation | South Korea | RCTs/experimental | TBI (non-specific) | Neuromodulation and neurofeedback | Depression |
| Lee (2019) | Systematic review | Multiple Countries | Systematic review | Stroke | Social support programs | Depression; quality of life |
| Lee (2019) | Impact evaluation | South Korea | RCTs/experimental | Stroke | Exercise and movement-based; tele-health and technology-based | Depression |
| Lee (2021) | Systematic review with meta-analysis | Multiple Countries | Systematic review with meta-analysis | Stroke | Exercise and movement-based; neuromodulation and neurofeedback; withdrawal management centres | Depression |
| Lee (2023) | Impact evaluation | South Korea | RCTs/experimental | Stroke | Exercise and movement-based; acupuncture and massage | Somatic symptoms; depression |
| Lenzo (2019) | Impact evaluation | Italy | RCTs/experimental | Stroke | Long-term in-patient services | Anxiety; depression |
| Levitt (2019) | Clinical trial registry | United States of America | RCTs/experimental | Stroke | Antidepressants | Depression |
| Levy (2021) | Impact evaluation | Canada | RCTs/experimental | Moderate TBI; severe TBI | Peer-to-peer programs | Community engagement |
| Li (2015) | Impact evaluation | China | RCTs/experimental | Stroke | Tele-health and technology-based | Depression |
| Li (2017) | Impact evaluation | China | RCTs/experimental | Stroke | Acupuncture and massage | Depression |
| Li (2018) | Impact evaluation | China | RCTs/experimental | Stroke | Herbal medicine | Depression |
| Li (2018) | Systematic review with meta-analysis | Multiple Countries | Systematic review with meta-analysis | Stroke | Acupuncture and massage | Depression |
| Li (2018) | Clinical trial registry | China | RCTs/experimental | Stroke | Tele-health and technology-based | Depression; cognition |
| Li (2019) | Impact evaluation | China | RCTs/experimental | Stroke | Acute in-patient services | Anxiety; depression |
| Li (2020) | Systematic review with meta-analysis | Multiple Countries | Systematic review with meta-analysis | Stroke | Antidepressants | Depression |
| Li (2020) | Meta analysis | Multiple Countries | Meta analysis | Stroke | Antidepressants | Depression |
| Li (2020) | Impact evaluation | China | RCTs/experimental | Stroke | Acute in-patient services | Quality of life |
| Li (2021) | Impact evaluation | China | RCTs/experimental | Stroke | Acute in-patient services | Depression |
| Li (2022) | Published study protocol | China | RCTs/experimental | Stroke | Acupuncture and massage | Anxiety |
| Li (2022) | Meta analysis | Multiple Countries | Meta analysis | Stroke | Exercise and movement-based | Depression |
| Li (2022) | Impact evaluation | Ireland | RCTs/experimental | Stroke | Psychotherapy (out-patient) | Depression; anxiety; cognition |
| Li (2022) | Systematic review with meta-analysis | Multiple Countries | Systematic review with meta-analysis | Stroke | Neuromodulation and neurofeedback | Depression |
| Li (2022) | Impact evaluation | China | RCTs/experimental | Stroke | Neuromodulation and neurofeedback | Depression |
| Li (2022) | Impact evaluation | China | RCTs/experimental | Stroke | Neuromodulation and neurofeedback | Depression |
| Li (2023) | Meta analysis | China | Systematic review with meta-analysis | Stroke | Acupuncture and massage; antidepressants | Depression |
| Li (2024) | Impact evaluation | China | RCTs/experimental | Stroke | Psychotherapy (in-patient) | Trauma and stress related |
| Li (2024) | Meta analysis | China | Systematic review with meta-analysis | Stroke | Psychotherapy (out-patient) | Depression |
| Li (2024) | Published study protocol | China | RCTs/experimental | Stroke | Acupuncture and massage | Depression |
| Li (2024) | Impact evaluation | China | RCTs/experimental | Stroke | Neuromodulation and neurofeedback | Depression; cognition |
| Li (2024) | Impact evaluation | China | Upper-tier quasi-experimental designs | Stroke | Acupuncture and massage | Depression |
| Li (2024) | Impact evaluation | China | RCTs/experimental | Stroke | Acute in-patient services | Depression; anxiety |
| Liang (2019) | Impact evaluation | China | RCTs/experimental | Stroke | Herbal medicine | Depression |
| Liang 202 | Systematic review with meta-analysis | Multiple Countries | Systematic review with meta-analysis | Stroke | Acute in-patient services | Depression |
| Liang (2022) | Systematic review with meta-analysis | Multiple Countries | Systematic review with meta-analysis | Stroke | Neuromodulation and neurofeedback; antidepressants | Depression |
| Lin (2017) | Impact evaluation | China | RCTs/experimental | Stroke | Acupuncture and massage; music and art | Depression |
| Lin (2018) | Impact evaluation | China | RCTs/experimental | Stroke | Acupuncture and massage; exercise and movement-based | Depression |
| Lin (2019) | Impact evaluation | Taiwan | RCTs/experimental | Stroke | Social support programs | Depression |
| Lin (2022) | Impact evaluation | China | RCTs/experimental | Stroke | Acute in-patient services | Self-awareness and self-efficacy |
| Linder (2015) | Impact evaluation | United States of America | RCTs/experimental | Stroke | Home-based care; exercise and movement-based | Depression; activities of daily living/life skills |
| Linton (2020) | Impact evaluation | United States of America | Upper-tier quasi-experimental designs | TBI (non-specific) | Integrated care and case management | Depression; quality of life |
| Little (2021) | Systematic review with meta-analysis | Multiple Countries | Systematic review with meta-analysis | TBI (non-specific) | Psychotherapy (in-patient) | Anxiety |
| Liu (2018) | Systematic review with meta-analysis | Multiple Countries | Systematic review with meta-analysis | TBI (non-specific) | Psychotherapy (in-patient) | Depression; anxiety; quality of life |
| Liu (2019) | Systematic review with meta-analysis | Multiple Countries | Systematic review with meta-analysis | Stroke | Neuromodulation and neurofeedback | Depression |
| Liu (2020) | Impact evaluation | China | RCTs/experimental | Stroke | Acupuncture and massage | Anxiety |
| Liu (2020) | Impact evaluation | China | RCTs/experimental | Stroke | Neuromodulation and neurofeedback; antidepressants | Depression |
| Liu (2021) | Systematic review with meta-analysis | Multiple Countries | Systematic review with meta-analysis | Stroke | Acupuncture and massage | Depression |
| Liu (2023) | Impact evaluation | China | RCTs/experimental | Stroke | Psychotherapy (in-patient) | Depression |
| Liu (2023) | Systematic review with meta-analysis | China | Systematic review with meta-analysis | Stroke | Tele-health and technology-based | Depression |
| Liu (2024) | Impact evaluation | China | RCTs/experimental | Stroke | Exercise and movement-based | Depression |
| Liu (2024) | Impact evaluation | China | Non-experimental designs | Stroke | Non-psychotropic medications | Depression |
| Liu (2024) | Impact evaluation | China | RCTs/experimental | Stroke | Neuromodulation and neurofeedback | Depression |
| Lo (2021) | Published study protocol | China | RCTs/experimental | Stroke | Social support programs | Relationships; depression |
| Love (2019) | Systematic review | Multiple Countries | Systematic review | Stroke | Mindfulness and meditation; exercise and movement-based | Depression; anxiety; quality of life |
| Lu (2017) | Impact evaluation | Taiwan | Lower-tier quasi-experimental designs | Stroke | Acupuncture and massage | Depression |
| Lu (2019) | Published study protocol | China | RCTs/experimental | Stroke | Acupuncture and massage | Depression |
| Lu (2020) | Impact evaluation | China | RCTs/experimental | Stroke | Neuromodulation and neurofeedback; antidepressants | Cognition; depression |
| Lu (2023) | Published study protocol | China | RCTs/experimental | Stroke | Acupuncture and massage; neuromodulation and neurofeedback | Depression |
| Lu (2023) | Impact evaluation | China | Non-experimental designs | Stroke | Tele-health and technology-based; home-based care | Healthcare utilization |
| Luauté (2016) | Clinical practice guideline | France | Clinical practice guideline | TBI (non-specific) | Social support programs | Quality of life |
| Ludwig (2024) | Impact evaluation | United States of America | RCTs/experimental | mTBI or concussion | Psychotherapy (out-patient) | Sleep and eating |
| Lundstrom (2021) | Impact evaluation | Sweden | RCTs/experimental | Stroke | Antidepressants | Depression |
| Luo (2015) | Impact evaluation | China | Lower-tier quasi-experimental designs | TBI (non-specific) | Antidepressants; psychotherapy (out-patient) | Depression |
| Luo (2022) | Impact evaluation | China | Upper-tier quasi-experimental designs | Stroke | Patient-caregiver interventions | Quality of life |
| Luo (2022) | Published study protocol | China | RCTs/experimental | Stroke | Acupuncture and massage | Depression |
| Lv (2022) | Impact evaluation | China | Non-experimental designs | Stroke | Non-psychotropic medications | Depression; anxiety |
| Ma (2018) | Impact evaluation | China | RCTs/experimental | Stroke | Antidepressants | Depression; anxiety |
| Ma (2024) | Impact evaluation | China | RCTs/experimental | Stroke | Acupuncture and massage; antidepressants | Depression |
| Maccaron (2024) | Impact evaluation | Croatia | Lower-tier quasi-experimental designs | Stroke | Psychotherapy (in-patient); exercise and movement-based | Depression; anxiety |
| Maksimowski (2016) | Systematic review | Multiple Countries | Systematic review | TBI (non-specific) | Psychostimulants | Cognition; depression |
| Malarkey (2024) | Impact evaluation | United States of America | RCTs/experimental | mTBI or concussion | Psychotherapy (out-patient); tele-health and technology-based | Sleep and eating |
| Man (2014) | Impact evaluation | China | RCTs/experimental | Stroke | Acupuncture and massage | Depression |
| Manu (2019) | Clinical trial registry | India | RCTs/experimental | Stroke | Herbal medicine | Depression |
| Mao (2024) | Impact evaluation | China | Lower-tier quasi-experimental designs | Stroke | Music and art | Sleep and eating; depression; anxiety |
| Marshall (2014) | Impact evaluation | United States of America | Lower-tier quasi-experimental designs | Stroke | Mindfulness and meditation | Anxiety; cognition |
| Mavaddat (2017) | Impact evaluation | United Kingdom | Non-experimental designs | Stroke | Mindfulness and meditation | Depression |
| Mayo (2015) | Impact evaluation | Canada | RCTs/experimental | Stroke | Social support programs; exercise and movement-based | Community engagement |
| Mccarron (2019) | Impact evaluation | United States of America | RCTs/experimental | TBI (non-specific) | Social support programs | Activities of daily living/life skills |
| Mcgeary (2022) | Impact evaluation | United States of America | RCTs/experimental | mTBI or concussion | Psychotherapy (out-patient) | Trauma and stress related |
| Mead (2022) | Impact evaluation | United Kingdom | RCTs/experimental | Stroke | Tele-health and technology-based | Quality of life |
| Mead (2022) | Impact evaluation | United Kingdom | RCTs/experimental | Stroke | Psychotherapy (out-patient) | Depression; anxiety |
| Meek (2021) | Impact evaluation | Canada | RCTs/experimental | mTBI or concussion | Neuromodulation and neurofeedback | Somatic symptoms; depression |
| Mehta (2022) | Clinical trial registry | Canada | RCTs/experimental | Stroke | Tele-health and technology-based; psychotherapy (out-patient) | Depression; anxiety |
| Menn (2014) | Impact evaluation | United States of America | RCTs/experimental | Moderate TBI | Non-psychotropic medications | Sleep and eating |
| Mensahotabil (2023) | Impact evaluation | United States of America | RCTs/experimental | Stroke | Tele-health and technology-based | Anxiety |
| Mercier (2020) | Published study protocol | Canada | RCTs/experimental | mTBI or concussion | Exercise and movement-based | Somatic symptoms |
| Messina (2020) | Impact evaluation | Italy | Upper-tier quasi-experimental designs | Stroke | Psychotherapy (in-patient); patient-caregiver interventions | Self-awareness and self-efficacy; quality of life; physical activity |
| Mi (2024) | Systematic review with meta-analysis | China | More than one of the above-mentioned categories | Stroke | Social support programs | Quality of life |
| Mikami (2014) | Impact evaluation | United States of America | RCTs/experimental | Stroke | Antidepressants; cognitive training/rehabilitation | Anxiety |
| Mikolic (2019) | Systematic review | Multiple Countries | Systematic review | TBI (non-specific) | Psychotherapy (out-patient) | Trauma and stress related |
| Minshall (2020) | Impact evaluation | United Kingdom | RCTs/experimental | Stroke | Psychotherapy (out-patient) | Quality of life |
| Mohabbat (2021) | Clinical trial registry | United States of America | RCTs/experimental | Stroke | Psychotherapy (in-patient) | Anxiety; depression; quality of life |
| Moore (2014) | Impact evaluation | United States of America | RCTs/experimental | mTBI or concussion | Acute in-patient services | Addiction-related |
| Moriarty (2021) | Clinical trial registry | United States of America | RCTs/experimental | TBI (non-specific) | Psychotherapy (out-patient); exercise and movement-based | Depression |
| Morris (2019) | Impact evaluation | United Kingdom | RCTs/experimental | Stroke | Music and art | Depression; activities of daily living/life skills; self-awareness and self-efficacy |
| Moshtaghi (2024) | Impact evaluation | Iran | Lower-tier quasi-experimental designs | Stroke | Psychotherapy (in-patient); antidepressants; neuromodulation and neurofeedback | Depression; cognition |
| Mou (2021) | Systematic review with meta-analysis | Multiple Countries | Systematic review with meta-analysis | Stroke | Patient-caregiver interventions | Quality of life |
| Mroczkowska (2019) | Impact evaluation | Poland | Lower-tier quasi-experimental designs | Stroke | Psychotherapy (out-patient); cognitive training/rehabilitation | Depression |
| Mueller (2022) | Clinical trial registry | United States of America | RCTs/experimental | TBI (non-specific) | Non-psychotropic medications | Cognition |
| Muresanu (2022) | Impact evaluation | Romania | RCTs/experimental | Moderate TBI; severe TBI | Non-psychotropic medications | Anxiety; depression |
| Narapareddy (2020) | Systematic review | Multiple Countries | Systematic review | TBI (non-specific) | Antidepressants; neuromodulation and neurofeedback | Depression |
| Neumann (2017) | Impact evaluation | United States of America | Lower-tier quasi-experimental designs | Moderate TBI; severe TBI | Mindfulness and meditation | Self-awareness and self-efficacy |
| Neumann (2018) | Clinical trial registry | United States of America | RCTs/experimental | TBI (non-specific) | Psychotherapy (out-patient) | Depression |
| Neumann (2024) | Impact evaluation | United States of America | RCTs/experimental | TBI (non-specific) | Psychotherapy (out-patient) | Depression |
| Ng (2017) | Impact evaluation | Australia | RCTs/experimental | Stroke | Psychotherapy (in-patient) | Quality of life; depression |
| Niu (2022) | Impact evaluation | China | RCTs/experimental | Stroke | Psychotherapy (in-patient) | Depression |
| Novakovic-agopian (2021) | Impact evaluation | United States of America | RCTs/experimental | TBI (non-specific) | Cognitive training/rehabilitation | Cognition; trauma and stress related |
| Odarushchenko (2023) | Impact evaluation | Russia | Upper-tier quasi-experimental designs | Stroke | Tele-health and technology-based; psychotherapy (out-patient) | Depression; anxiety |
| Oh (2022) | Systematic review with meta-analysis | Multiple Countries | Systematic review with meta-analysis | Stroke | Psychotherapy (out-patient) | Quality of life; depression; anxiety |
| Olukolade (2017) | Impact evaluation | Nigeria | RCTs/experimental | Stroke | Cognitive training/rehabilitation | Depression |
| Özakgül (2018) | Impact evaluation | Turkey | RCTs/experimental | Stroke | Patient-caregiver interventions | Depression |
| Ozen (2016) | Impact evaluation | Canada | RCTs/experimental | TBI (non-specific) | Mindfulness and meditation | Depression |
| Palumbo (2022) | Impact evaluation | United States of America | RCTs/experimental | Stroke | Music and art | Depression; quality of life |
| Pan (2023) | Systematic review with meta-analysis | China | Systematic review with meta-analysis | Stroke | Neuromodulation and neurofeedback; antidepressants | Depression |
| Paraschakis (2017) | Systematic review with meta-analysis | Multiple Countries | Systematic review with meta-analysis | TBI (non-specific) | Antidepressants | Depression |
| Parkinson (2022) | Impact evaluation | United Kingdom | Non-experimental designs | Stroke | Patient-caregiver interventions ; mindfulness and meditation; tele-health and technology-based | Anxiety; depression |
| Payne (2020) | Impact evaluation | United States of America | RCTs/experimental | TBI (non-specific) | Social support programs | Quality of life; community engagement |
| Peng (2014) | Systematic review | China | Systematic review with meta-analysis | Stroke | Herbal medicine | Depression |
| Pennington (2020) | Impact evaluation | United States of America | RCTs/experimental | TBI (non-specific) | Antipsychotics and anticonvulsants | Addiction-related |
| Pennington (2022) | Impact evaluation | United States of America | RCTs/experimental | TBI (non-specific) | Exercise and movement-based; tele-health and technology-based | Addiction-related |
| Peppel (2020) | Systematic review with meta-analysis | Multiple Countries | Systematic review with meta-analysis | Moderate TBI; severe TBI | Antidepressants; psychotherapy (out-patient) | Depression |
| Perez-de la cruz (2020) | Impact evaluation | Spain | RCTs/experimental | Stroke | Exercise and movement-based | Quality of life; somatic symptoms |
| Perez-rodriguez (2021) | Impact evaluation | Spain | Upper-tier quasi-experimental designs | ABI (non-specific) | Exercise and movement-based | Depression |
| Perovic (2017) | Impact evaluation | Montenegro | Non-experimental designs | Stroke | Acute in-patient services | Anxiety; depression |
| Perry (2020) | Systematic review with meta-analysis | Multiple Countries | Systematic review with meta-analysis | TBI (non-specific) | Exercise and movement-based | Depression |
| Philip (2023) | Impact evaluation | United States of America | Lower-tier quasi-experimental designs | mTBI or concussion | Neuromodulation and neurofeedback | Trauma and stress related; depression |
| Phyland (2023) | Impact evaluation | Australia | RCTs/experimental | TBI (non-specific) | Antipsychotics and anticonvulsants | Aggression |
| Pilon (2023) | Impact evaluation | Netherlands | RCTs/experimental | ABI (non-specific) | Psychotherapy (out-patient) | Sleep and eating |
| Pisegna (2023) | Impact evaluation | United States of America | Upper-tier quasi-experimental designs | Stroke | Acute in-patient services | Depression; anxiety |
| Plantier (2016) | Systematic review | Multiple Countries | Systematic review | TBI (non-specific) | Antipsychotics and anticonvulsants; non-psychotropic medications | Aggression; psychosis-related |
| Ponsford (2016) | Impact evaluation | Australia | RCTs/experimental | TBI (non-specific) | Psychotherapy (in-patient) | Depression; anxiety |
| Ponsford (2020) | Impact evaluation | Australia | Upper-tier quasi-experimental designs | TBI (non-specific) | Psychotherapy (in-patient) | Cognition; depression; anxiety |
| Porter (2024) | Impact evaluation | Usa | RCTs/experimental | mTBI or concussion | Antidepressants; psychotherapy (out-patient) | Trauma and stress related |
| Pui kei (2020) | Published study protocol | Australia | RCTs/experimental | Stroke | Home-based care | Self-awareness and self-efficacy; anxiety |
| Qian (2015) | Impact evaluation | China | RCTs/experimental | Stroke | Acupuncture and massage; antidepressants | Depression |
| Qin (2018) | Meta analysis | Multiple Countries | Meta analysis | Stroke | Antidepressants | Depression |
| Qin (2021) | Systematic review | Multiple Countries | Systematic review | Stroke | Cognitive training/rehabilitation; social support programs | Quality of life; anxiety |
| Qiqi (2021) | Meta analysis | Multiple Countries | Meta analysis | Stroke | Psychotherapy (out-patient) | Depression |
| Qiu (2023) | Impact evaluation | China | RCTs/experimental | Stroke | Neuromodulation and neurofeedback | Depression |
| Quinn (2020) | Impact evaluation | United States of America | RCTs/experimental | mTBI or concussion | Neuromodulation and neurofeedback; cognitive training/rehabilitation | Depression |
| Rafiq (2020) | Impact evaluation | Pakistan | Upper-tier quasi-experimental designs | Stroke | Psychotherapy (out-patient) | Depression; anxiety |
| Raglio (2017) | Impact evaluation | Italy | RCTs/experimental | Stroke | Music and art | Quality of life; depression; anxiety |
| Raikes (2020) | Impact evaluation | United States of America | RCTs/experimental | mTBI or concussion | Tele-health and technology-based | Sleep and eating; depression |
| Rao (2019) | Impact evaluation | United States of America | RCTs/experimental | TBI (non-specific) | Neuromodulation and neurofeedback | Depression |
| Rash (2022) | Published study protocol | Canada | RCTs/experimental | Stroke | Tele-health and technology-based | Depression; physical activity |
| Rauwenhoff (2019) | Published study protocol | Netherlands | RCTs/experimental | ABI (non-specific) | Psychotherapy (out-patient) | Depression; anxiety |
| Rauwenhoff (2024) | Impact evaluation | Netherlands | RCTs/experimental | ABI (non-specific) | Psychotherapy (out-patient) | Depression; anxiety |
| Raya-ruiz (2022) | Published study protocol | Spain | RCTs/experimental | ABI (non-specific) | Social support programs | Quality of life |
| Reeves (2019) | Impact evaluation | United States of America | RCTs/experimental | Stroke | Long-term in-patient services | Quality of life; depression |
| Reyes (2019) | Systematic review with meta-analysis | Multiple Countries | Systematic review with meta-analysis | TBI (non-specific) | Antidepressants | Depression; quality of life |
| Rice (2022) | Impact evaluation | Tanzania | Upper-tier quasi-experimental designs | Stroke | Antidepressants | Depression |
| Richter (2021) | Systematic review with meta-analysis | Multiple Countries | Systematic review with meta-analysis | Stroke | Antidepressants | Depression |
| Rodrigues (2020) | Impact evaluation | Brazil | RCTs/experimental | Moderate TBI; severe TBI | Neuromodulation and neurofeedback | Anxiety |
| Ross (2023) | Impact evaluation | United States of America | RCTs/experimental | Stroke | Exercise and movement-based; neuromodulation and neurofeedback | Physical activity; depression |
| Roy (2022) | Impact evaluation | United States of America | RCTs/experimental | mTBI or concussion | Psychotherapy (out-patient); tele-health and technology-based | Trauma and stress related |
| Rushing (2022) | Impact evaluation | United States of America | Upper-tier quasi-experimental designs | Stroke | Music and art | Depression |
| Ryan (2022) | Systematic review | Multiple Countries | Systematic review | Stroke | Psychotherapy (out-patient) | Anxiety |
| Rzezak (2015) | Impact evaluation | Brazil | Lower-tier quasi-experimental designs | TBI (non-specific) | Exercise and movement-based | Anxiety |
| Saha (2022) | Clinical practice guideline | India | Clinical practice guideline | ABI (non-specific) | Antipsychotics and anticonvulsants; antidepressants | Psychosis-related; depression; anxiety |
| Salter (2016) | Systematic review with meta-analysis | Multiple Countries | Systematic review with meta-analysis | TBI (non-specific) | Antidepressants | Depression |
| Sander (2021) | Impact evaluation | United States of America | RCTs/experimental | Moderate TBI | Psychotherapy (out-patient) | Depression |
| Sarkamo (2021) | Impact evaluation | Finland | RCTs/experimental | Severe TBI | Exercise and movement-based | Cognition; physical activity |
| Sasaki (2017) | Impact evaluation | Japan | RCTs/experimental | Stroke | Neuromodulation and neurofeedback | Depression |
| Scheenen (2017) | Impact evaluation | Netherlands | RCTs/experimental | mTBI or concussion | Tele-health and technology-based; psychotherapy (in-patient) | Anxiety; depression; trauma and stress related |
| Schwertfeger (2024) | Impact evaluation | United States of America | Lower-tier quasi-experimental designs | Stroke | Social support programs | Quality of life |
| Schy (2023) | Clinical trial registry | United States of America | RCTs/experimental | mTBI or concussion | Neuromodulation and neurofeedback; cognitive training/rehabilitation | Depression |
| Seidi (2023) | Impact evaluation | Iran | RCTs/experimental | Stroke | Acute in-patient services | Anxiety |
| Setiyowati (2019) | Impact evaluation | Taiwan | Lower-tier quasi-experimental designs | Stroke | Exercise and movement-based | Depression |
| Shabani (2022) | Impact evaluation | Iran | RCTs/experimental | Stroke | Antidepressants | Quality of life |
| Shao (2020) | Systematic review with meta-analysis | Multiple Countries | Systematic review with meta-analysis | Stroke | Herbal medicine | Depression |
| Shao (2021) | Systematic review with meta-analysis | Multiple Countries | Systematic review with meta-analysis | Stroke | Neuromodulation and neurofeedback | Depression |
| Shaw (2021) | Clinical trial registry | United States of America | RCTs/experimental | TBI (non-specific) | Tele-health and technology-based; psychotherapy (out-patient) | Depression |
| Shek (2021) | Systematic review with meta-analysis | Multiple Countries | Systematic review with meta-analysis | Stroke | Tele-health and technology-based | Depression |
| Shen (2017) | Systematic review with meta-analysis | Multiple Countries | Systematic review with meta-analysis | Stroke | Neuromodulation and neurofeedback | Depression |
| Shen (2022) | Systematic review with meta-analysis | Multiple Countries | Systematic review with meta-analysis | Stroke | Neuromodulation and neurofeedback | Depression |
| Sherer (2024) | Impact evaluation | United States of America | RCTs/experimental | TBI (non-specific) | Tele-health and technology-based | Depression |
| Shin (2015) | Impact evaluation | South Korea | RCTs/experimental | Stroke | Tele-health and technology-based | Physical activity; depression |
| Shiroff (2023) | Impact evaluation | United States of America | RCTs/experimental | TBI (non-specific) | Social support programs | Trauma and stress related |
| Shirvani (2021) | Impact evaluation | Iran | RCTs/experimental | mTBI or concussion | Neuromodulation and neurofeedback; mindfulness and meditation | Sleep and eating; quality of life; aggression |
| Si (2022) | Impact evaluation | China | RCTs/experimental | Stroke | Patient-caregiver interventions | Quality of life |
| Sianturi (2018) | Impact evaluation | Indonesia | Lower-tier quasi-experimental designs | Severe TBI | Psychotherapy (in-patient) | Depression |
| Siddiqi (2019) | Impact evaluation | United States of America | RCTs/experimental | TBI (non-specific) | Neuromodulation and neurofeedback | Depression |
| Siddiqi (2023) | Impact evaluation | United States of America | RCTs/experimental | TBI (non-specific) | Neuromodulation and neurofeedback | Depression |
| Silverberg (2021) | Clinical trial registry | Canada | RCTs/experimental | mTBI or concussion | Acute in-patient services | Depression |
| Silverberg (2022) | Published study protocol | Canada | RCTs/experimental | mTBI or concussion | Social support programs | Depression; anxiety |
| Simblett (2017) | Impact evaluation | United Kingdom | RCTs/experimental | Stroke | Psychotherapy (out-patient); tele-health and technology-based | Anxiety; depression; activities of daily living/life skills |
| Skidmore (2015) | Impact evaluation | United States of America | RCTs/experimental | Stroke | Acute in-patient services | Depression |
| Slowinski (2019) | Meta analysis | Multiple Countries | Meta analysis | TBI (non-specific) | Antidepressants | Depression |
| Smeets (2017) | Impact evaluation | Netherlands | Non-experimental designs | ABI (non-specific) | Integrated care and case management; psychotherapy (out-patient) | Self-awareness and self-efficacy |
| Song (2021) | Impact evaluation | China | Non-experimental designs | Stroke | Acupuncture and massage | Sleep and eating |
| Song (2022) | Impact evaluation | China | Non-experimental designs | Stroke | Acute in-patient services | Depression; anxiety |
| Song (2023) | Systematic review with meta-analysis | China | Systematic review with meta-analysis | Stroke | Exercise and movement-based | Quality of life; depression; anxiety |
| Speicher (2014) | Impact evaluation | United States of America | RCTs/experimental | TBI (non-specific) | Integrated care and case management | Trauma and stress related; depression |
| Srisurapanont (2021) | Systematic review with meta-analysis | Multiple Countries | Systematic review with meta-analysis | TBI (non-specific) | Tele-health and technology-based | Sleep and eating |
| Stahl (2022) | Impact evaluation | United Kingdom | Upper-tier quasi-experimental designs | Stroke | Social support programs | Depression |
| Stiekema (2020) | Published study protocol | Netherlands | RCTs/experimental | ABI (non-specific) | Integrated care and case management | Depression |
| Storm (2019) | Systematic review | Multiple Countries | Systematic review | Stroke | Exercise and movement-based | Depression; self-awareness and self-efficacy |
| Su (2021) | Systematic review with meta-analysis | Multiple Countries | Systematic review with meta-analysis | Stroke | Acupuncture and massage | Depression |
| Su (2024) | Systematic review with meta-analysis | China | Systematic review with meta-analysis | Stroke | Herbal medicine; acupuncture and massage | Sleep and eating |
| Su (2024) | Systematic review | China | Systematic review with meta-analysis | Stroke | Exercise and movement-based | Quality of life; depression |
| Suarilah (2024) | Systematic review with meta-analysis | Taiwan | Systematic review with meta-analysis | TBI (non-specific) | Tele-health and technology-based | Somatic symptoms; depression |
| Sullan (2021) | Impact evaluation | United States of America | RCTs/experimental | TBI (non-specific) | Psychotherapy (in-patient) | Sleep and eating; trauma and stress related |
| Sumakul (2020) | Impact evaluation | Indonesia | Upper-tier quasi-experimental designs | Stroke | Music and art | Depression |
| Sun (2017) | Systematic review with meta-analysis | Multiple Countries | Systematic review with meta-analysis | Stroke | Antidepressants | Depression |
| Sun (2022) | Impact evaluation | China | RCTs/experimental | Stroke | Psychotherapy (out-patient) | Depression |
| Sun (2022) | Impact evaluation | China | RCTs/experimental | Stroke | Exercise and movement-based | Depression |
| Sun (2024) | Impact evaluation | China | RCTs/experimental | Stroke | #ref! | Depression |
| Sun (2024) | Impact evaluation | China | Lower-tier quasi-experimental designs | Stroke | Acute in-patient services | Anxiety; depression |
| Tadayon (2022) | Published study protocol | Iran | RCTs/experimental | Stroke | Tele-health and technology-based | Physical activity; activities of daily living/life skills; quality of life |
| Tan (2015) | Meta analysis | Multiple Countries | Meta analysis | Stroke | Antidepressants | Depression |
| Tan (2022) | Impact evaluation | Singapore | RCTs/experimental | Stroke | Non-psychotropic medications | Depression; anxiety |
| Tang (2018) | Published study protocol | China | RCTs/experimental | Stroke | Neuromodulation and neurofeedback | Depression; somatic symptoms; cognition |
| Tao (2021) | Published study protocol | China | RCTs/experimental | Stroke | Acupuncture and massage | Depression |
| Tao (2022) | Systematic review with meta-analysis | Multiple Countries | Systematic review with meta-analysis | Stroke | Mindfulness and meditation | Depression |
| Tao (2022) | Impact evaluation | China | RCTs/experimental | Stroke | Acupuncture and massage | Depression |
| Tay (2022) | Impact evaluation | United Kingdom | RCTs/experimental | Stroke | Antidepressants | Depression |
| Taylor-piliae (2021) | Impact evaluation | United States of America | Non-experimental designs | Stroke | Exercise and movement-based | Depression; sleep and eating |
| Tazopoulou (2016) | Impact evaluation | France | Non-experimental designs | Hypoxic-anoxic | Long-term in-patient services | Cognition; community engagement; depression |
| Termors (2019) | Systematic review | Multiple Countries | Systematic review | ABI (non-specific) | Non-psychotropic medications | Aggression |
| Terrill (2022) | Impact evaluation | United States of America | RCTs/experimental | Stroke | Patient-caregiver interventions | Depression |
| Tham (2023) | Impact evaluation | Singapore | Lower-tier quasi-experimental designs | Stroke | Tele-health and technology-based | Depression |
| The university of hong kong ((2022)) | Clinical trial registry | China | RCTs/experimental | Stroke | Tele-health and technology-based | Depression |
| Thomas (2019) | Impact evaluation | United Kingdom | RCTs/experimental | Stroke | Psychotherapy (in-patient) | Depression |
| Thuan (2024) | Meta analysis | Vietnam | Systematic review with meta-analysis | Stroke | Tele-health and technology-based | Depression; quality of life |
| Tian (2022) | Impact evaluation | China | Non-experimental designs | Stroke | Antidepressants; psychotherapy (in-patient) | Depression |
| Tjokrowijoto (2023) | Impact evaluation | United Kingdom | Non-experimental designs | Stroke | Psychotherapy (out-patient) | Depression |
| Torabi (2023) | Impact evaluation | Iran | RCTs/experimental | Stroke | Mindfulness and meditation | Anxiety |
| Tornas (2016) | Impact evaluation | Norway | RCTs/experimental | ABI (non-specific) | Cognitive training/rehabilitation | Cognition |
| Torrisi (2021) | Impact evaluation | Italy | RCTs/experimental | Stroke | Non-psychotropic medications | Depression; self-awareness and self-efficacy |
| Torrisi (2022) | Impact evaluation | Italy | Non-experimental designs | Stroke | Acute in-patient services | Depression; cognition |
| Towfighi (2017) | Systematic review | Multiple Countries | Systematic review | Stroke | Integrated care and case management | Depression |
| Tracey (2023) | Systematic review | United States of America | Systematic review with meta-analysis | mTBI or concussion | Cognitive training/rehabilitation; psychotherapy (in-patient) | Cognition |
| Trihandini (2018) | Impact evaluation | Indonesia | Upper-tier quasi-experimental designs | Stroke | Acute in-patient services | Anxiety |
| Trofimova (2021) | Impact evaluation | Russia | RCTs/experimental | Stroke | Antidepressants; non-psychotropic medications | Depression |
| Tsai (2021) | Systematic review with meta-analysis | Multiple Countries | Systematic review with meta-analysis | TBI (non-specific) | Neuromodulation and neurofeedback | Depression |
| Tsai (2022) | Impact evaluation | Taiwan | Upper-tier quasi-experimental designs | Stroke | Integrated care and case management | Depression; quality of life |
| Tsaousides (2017) | Impact evaluation | United States of America | Lower-tier quasi-experimental designs | TBI (non-specific) | Tele-health and technology-based; psychotherapy (out-patient) | Self-awareness and self-efficacy; quality of life |
| Tseng (2017) | Impact evaluation | Taiwan | Lower-tier quasi-experimental designs | Stroke | Acupuncture and massage | Depression |
| Tulip (2020) | Impact evaluation | United Kingdom | Non-experimental designs | ABI (non-specific) | Psychotherapy (out-patient) | Self-awareness and self-efficacy |
| Twamley (2014) | Impact evaluation | United States of America | RCTs/experimental | TBI (non-specific) | Social support programs | Cognition; depression |
| Uchida (2020) | Published study protocol | Japan | RCTs/experimental | Stroke | Tele-health and technology-based | Depression |
| Udvardi (2024) | Impact evaluation | Hungary | RCTs/experimental | Stroke | Psychotherapy (in-patient) | Depression |
| Uzdavines (2021) | Published study protocol | United States of America | RCTs/experimental | TBI (non-specific) | Psychotherapy (out-patient) | Depression; anxiety |
| Valiengo (2017) | Impact evaluation | Brazil | RCTs/experimental | Stroke | Neuromodulation and neurofeedback | Depression |
| Vallury (2015) | Systematic review | Multiple Countries | Systematic review | Stroke | Patient-caregiver interventions | Depression |
| Vaneeden (2015) | Impact evaluation | Netherlands | RCTs/experimental | Stroke | Psychotherapy (in-patient); exercise and movement-based | Depression; anxiety |
| Vanvugt (2014) | Impact evaluation | Germany | RCTs/experimental | Stroke | Music and art; exercise and movement-based | Depression |
| Vasu (2020) | Impact evaluation | Malaysia | Lower-tier quasi-experimental designs | Stroke | Exercise and movement-based | Depression |
| Vasu (2021) | Published study protocol | Malaysia | RCTs/experimental | Stroke | Mindfulness and meditation; exercise and movement-based | Anxiety; depression |
| Verberne (2019) | Systematic review | Multiple Countries | Systematic review | ABI (non-specific) | Psychotherapy (out-patient) | Anxiety; aggression |
| Verberne (2022) | Impact evaluation | Netherlands | RCTs/experimental | Stroke | Integrated care and case management | Depression; anxiety; activities of daily living/life skills |
| Visser (2016) | Impact evaluation | Belgium | RCTs/experimental | Stroke | Psychotherapy (out-patient) | Quality of life; self-awareness and self-efficacy |
| Vonmensenkampff (2015) | Impact evaluation | Ireland | Non-experimental designs | ABI (non-specific) | Psychotherapy (out-patient) | Trauma and stress related |
| Vujkovic (2022) | Impact evaluation | Bosnia and Herzegovina | Lower-tier quasi-experimental designs | Stroke | Non-psychotropic medications | Anxiety; depression |
| Waid-ebbs (2022) | Clinical trial registry | United States of America | RCTs/experimental | TBI (non-specific) | Cognitive training/rehabilitation | Cognition; community engagement |
| Wallace (2022) | Impact evaluation | United States of America | Lower-tier quasi-experimental designs | mTBI or concussion | Integrated care and case management | Depression; sleep and eating |
| Wallace (2022) | Impact evaluation | United States of America | RCTs/experimental | mTBI or concussion | Tele-health and technology-based | Trauma and stress related |
| Wan (2021) | Systematic review with meta-analysis | Multiple Countries | Systematic review with meta-analysis | Stroke | Peer-to-peer programs; patient-caregiver interventions | Somatic symptoms; depression |
| Wan (2021) | Systematic review with meta-analysis | Multiple Countries | Systematic review with meta-analysis | Stroke | Herbal medicine | Depression |
| Wan (2022) | Published study protocol | China | RCTs/experimental | Stroke | Peer-to-peer programs; patient-caregiver interventions | Relationships; self-awareness and self-efficacy |
| Wan (2024) | Systematic review with meta-analysis | China | Systematic review with meta-analysis | Stroke | Psychotherapy (out-patient) | Depression; quality of life |
| Wang (2018) | Systematic review with meta-analysis | Multiple Countries | Systematic review with meta-analysis | Stroke | Psychotherapy (out-patient) | Depression |
| Wang (2018) | Impact evaluation | China | Lower-tier quasi-experimental designs | Stroke | Acupuncture and massage; antidepressants | Depression |
| Wang (2019) | Meta analysis | Multiple Countries | Meta analysis | Stroke | Herbal medicine | Depression |
| Wang (2020) | Impact evaluation | Taiwan | RCTs/experimental | Stroke | Home-based care | Depression |
| Wang (2020) | Published study protocol | China | RCTs/experimental | Stroke | Neuromodulation and neurofeedback | Depression |
| Wang (2020) | Impact evaluation | China | RCTs/experimental | Stroke | Mindfulness and meditation | Depression |
| Wang (2021) | Systematic review with meta-analysis | Multiple Countries | Systematic review with meta-analysis | Stroke | Acupuncture and massage | Depression |
| Wang (2021) | Systematic review with meta-analysis | Multiple Countries | Systematic review with meta-analysis | Stroke | Acupuncture and massage; non-psychotropic medications | Depression |
| Wang (2022) | Systematic review with meta-analysis | Multiple Countries | Systematic review with meta-analysis | Stroke | Herbal medicine | Depression |
| Wang (2022) | Impact evaluation | China | RCTs/experimental | Stroke | Acupuncture and massage | Depression |
| Wang (2022) | Clinical trial registry | China | RCTs/experimental | Stroke | Neuromodulation and neurofeedback; acupuncture and massage | Depression |
| Wang (2022) | Impact evaluation | China | RCTs/experimental | Stroke | Psychotherapy (in-patient) | Sleep and eating; depression; anxiety |
| Wang (2023) | Impact evaluation | China | RCTs/experimental | Stroke | Herbal medicine; psychotherapy (in-patient) | Depression |
| Wang (2024) | Impact evaluation | China | Non-experimental designs | Stroke | Acute in-patient services | Depression |
| Wang (2024) | Impact evaluation | China | RCTs/experimental | Stroke | Psychotherapy (in-patient); herbal medicine | Depression |
| Ward (2016) | Impact evaluation | Australia | RCTs/experimental | Stroke | Psychotherapy (out-patient); patient-caregiver interventions | Depression |
| Weaver (2018) | Impact evaluation | United States of America | RCTs/experimental | mTBI or concussion | Acute in-patient services | Trauma and stress related; quality of life |
| Wei (2021) | Impact evaluation | China | RCTs/experimental | Stroke | Psychotherapy (in-patient) | Depression |
| Wei (2023) | Impact evaluation | China | RCTs/experimental | Stroke | Neuromodulation and neurofeedback; exercise and movement-based | Depression |
| Weightman (2023) | Published study protocol | United Kingdom | RCTs/experimental | Stroke | Tele-health and technology-based | Sleep and eating |
| Weinstein (2017) | Impact evaluation | United States of America | Upper-tier quasi-experimental designs | TBI (non-specific) | Exercise and movement-based | Depression; anxiety |
| Whiting (2020) | Impact evaluation | Australia | RCTs/experimental | Severe TBI | Psychotherapy (out-patient) | Depression; anxiety |
| Whiting (2021) | Published study protocol | Australia | RCTs/experimental | Severe TBI | Psychotherapy (out-patient) | Depression; anxiety |
| Wiart (2016) | Systematic review | Multiple Countries | Systematic review | TBI (non-specific) | Psychotherapy (out-patient) | Cognition; depression; aggression |
| Wichowicz (2017) | Impact evaluation | Poland | RCTs/experimental | Stroke | Psychotherapy (out-patient) | Depression; anxiety |
| Wilkie (2021) | Impact evaluation | United Kingdom | Non-experimental designs | ABI (non-specific) | Tele-health and technology-based | Quality of life |
| Wilkie (2022) | Impact evaluation | United Kingdom | Non-experimental designs | ABI (non-specific) | Exercise and movement-based | Depression; anxiety |
| Williamson (2019) | Systematic review | Multiple Countries | Systematic review | TBI (non-specific) | Psychostimulants; anxiolytics; antidepressants | Aggression |
| Winkens (2019) | Impact evaluation | Netherlands | Non-experimental designs | ABI (non-specific) | Acute in-patient services | Aggression |
| Winter (2020) | Impact evaluation | United States of America | Non-experimental designs | TBI (non-specific) | Home-based care; patient-caregiver interventions | Depression |
| Wium-andersen (2017) | Impact evaluation | Denmark | RCTs/experimental | Stroke | Non-psychotropic medications | Depression |
| Wolf (2015) | Impact evaluation | United States of America | RCTs/experimental | TBI (non-specific) | Psychotherapy (in-patient) | Trauma and stress related; depression |
| Wolf (2018) | Impact evaluation | United States of America | RCTs/experimental | TBI (non-specific) | Psychotherapy (out-patient) | Trauma and stress related; somatic symptoms; self-awareness and self-efficacy |
| Woranush (2021) | Systematic review | Multiple Countries | Systematic review | Stroke | Antidepressants; psychotherapy (out-patient) | Depression |
| Wrapson (2022) | Impact evaluation | New Zealand | Upper-tier quasi-experimental designs | Stroke | Mindfulness and meditation | Depression; anxiety |
| Wu (2017) | Impact evaluation | China | RCTs/experimental | Stroke | Acute in-patient services | Depression; anxiety |
| Wu (2021) | Impact evaluation | China | RCTs/experimental | Stroke | Acute in-patient services | Quality of life |
| Wu (2022) | Meta analysis | Multiple Countries | Meta analysis | Stroke | Herbal medicine | Depression |
| Wu (2022) | Impact evaluation | China | RCTs/experimental | Stroke | Tele-health and technology-based | Depression |
| Wu (2023) | Impact evaluation | China | RCTs/experimental | Stroke | Music and art; antidepressants | Depression; anxiety |
| Wu (2024) | Impact evaluation | China | Lower-tier quasi-experimental designs | Stroke | Music and art | Depression; anxiety |
| Xiao (2021) | Impact evaluation | China | RCTs/experimental | Stroke | Antidepressants | Depression |
| Xiao (2024) | Systematic review with meta-analysis | China | Systematic review with meta-analysis | Stroke | Acupuncture and massage; neuromodulation and neurofeedback | Depression |
| Xie (2022) | Impact evaluation | China | RCTs/experimental | Stroke | Herbal medicine; acupuncture and massage | Sleep and eating; anxiety |
| Xie (2024) | Impact evaluation | China | Non-experimental designs | Stroke | Psychotherapy (out-patient) | Depression; anxiety |
| Xin (2024) | Impact evaluation | China | Lower-tier quasi-experimental designs | Stroke | Non-psychotropic medications | Cognition |
| Xu (2016) | Systematic review with meta-analysis | Multiple Countries | Systematic review with meta-analysis | Stroke | Antidepressants | Depression |
| Xu (2018) | Impact evaluation | China | RCTs/experimental | Stroke | Antidepressants | Depression |
| Xu (2019) | Systematic review with meta-analysis | Multiple Countries | Systematic review with meta-analysis | Stroke | Herbal medicine | Depression |
| Xu (2024) | Impact evaluation | China | Upper-tier quasi-experimental designs | Stroke | Psychotherapy (out-patient) | Depression; anxiety |
| Xux-y (2023) | Impact evaluation | China | RCTs/experimental | Stroke | Long-term in-patient services | Depression; quality of life |
| Yak?üi (2023) | Impact evaluation | Turkey | Non-experimental designs | Stroke | Exercise and movement-based | Quality of life |
| Yan (2015) | Impact evaluation | China | RCTs/experimental | Stroke | Acute in-patient services; antidepressants | Depression |
| Yan (2024) | Impact evaluation | China | RCTs/experimental | Stroke | Antidepressants | Depression |
| Yang (2022) | Impact evaluation | China | Lower-tier quasi-experimental designs | Stroke | Acupuncture and massage | Depression |
| Yanyu (2023) | Meta analysis | China | Systematic review with meta-analysis | Stroke | Neuromodulation and neurofeedback | Depression |
| Yao (2021) | Impact evaluation | China | RCTs/experimental | Stroke | Antidepressants | Depression |
| Yao (2022) | Impact evaluation | China | RCTs/experimental | Stroke | Herbal medicine | Depression |
| Yao (2023) | Impact evaluation | Taiwan | Lower-tier quasi-experimental designs | Stroke | Acute in-patient services | Depression; quality of life |
| Yi (2024) | Systematic review with meta-analysis | China | Systematic review with meta-analysis | Stroke | Herbal medicine; psychotherapy (out-patient); neuromodulation and neurofeedback | Depression |
| Yilmaz (2020) | Clinical trial registry | Turkey | RCTs/experimental | Stroke | Acupuncture and massage | Anxiety |
| Yin (2022) | Impact evaluation | China | RCTs/experimental | Stroke | Acupuncture and massage | Depression |
| Yin (2024) | Impact evaluation | China | RCTs/experimental | Stroke | Herbal medicine | Depression; sleep and eating |
| Yisma (2024) | Systematic review with meta-analysis | United Kingdom | Systematic review with meta-analysis | Stroke | Psychotherapy (in-patient) | Depression |
| You (2020) | Impact evaluation | China | RCTs/experimental | Stroke | Acute in-patient services | Depression |
| You (2020) | Impact evaluation | China | RCTs/experimental | Stroke | Acupuncture and massage; antidepressants | Depression; quality of life |
| Yu (2019) | Impact evaluation | China | RCTs/experimental | Stroke | Patient-caregiver interventions | Depression; anxiety; cognition |
| Yu (2019) | Impact evaluation | China | RCTs/experimental | Stroke | Cognitive training/rehabilitation | Cognition; depression; anxiety |
| Yu (2021) | Impact evaluation | China | RCTs/experimental | Stroke | Neuromodulation and neurofeedback | Depression |
| Yu (2022) | Impact evaluation | China | RCTs/experimental | Stroke | Psychotherapy (out-patient) | Depression |
| Yuan (2023) | Impact evaluation | China | RCTs/experimental | Stroke | Antidepressants | Depression |
| Yue (2017) | Meta analysis | Multiple Countries | Meta analysis | TBI (non-specific) | Antidepressants | Depression |
| Yue (2024) | Impact evaluation | China | RCTs/experimental | Stroke | Psychotherapy (out-patient) | Depression |
| Zeng (2016) | Systematic review with meta-analysis | Multiple Countries | Systematic review with meta-analysis | Stroke | Non-psychotropic medications | Depression |
| Zeng (2017) | Systematic review | Multiple Countries | Systematic review | Stroke | Herbal medicine | Depression |
| Zeng (2022) | Clinical trial registry | China | RCTs/experimental | Stroke | Acupuncture and massage | Depression |
| Zeng (2022) | Clinical trial registry | China | RCTs/experimental | Stroke | Antidepressants | Depression |
| Zhang (2014) | Systematic review with meta-analysis | China | Systematic review with meta-analysis | Stroke | Acupuncture and massage | Depression |
| Zhang (2014) | Meta analysis | China | Systematic review with meta-analysis | Stroke | Acupuncture and massage | Depression |
| Zhang (2016) | Impact evaluation | China | RCTs/experimental | Stroke | Acupuncture and massage; antidepressants | Depression |
| Zhang (2017) | Impact evaluation | China | RCTs/experimental | Stroke | Acupuncture and massage | Depression |
| Zhang (2019) | Impact evaluation | China | RCTs/experimental | Stroke | Acupuncture and massage; antidepressants | Depression |
| Zhang (2019) | Systematic review with meta-analysis | Multiple Countries | Systematic review with meta-analysis | Stroke | Acupuncture and massage | Depression |
| Zhang (2020) | Impact evaluation | China | RCTs/experimental | Stroke | Neuromodulation and neurofeedback; non-psychotropic medications | Depression; quality of life |
| Zhang (2020) | Impact evaluation | China | RCTs/experimental | Stroke | Acupuncture and massage | Depression |
| Zhang (2021) | Systematic review with meta-analysis | Multiple Countries | Systematic review with meta-analysis | Stroke | Tele-health and technology-based | Cognition; depression |
| Zhang (2021) | Impact evaluation | China | RCTs/experimental | Stroke | Psychotherapy (out-patient); exercise and movement-based | Depression; activities of daily living/life skills |
| Zhang (2021) | Systematic review with meta-analysis | Multiple Countries | Systematic review with meta-analysis | Stroke | Acupuncture and massage; antidepressants | Depression |
| Zhang (2021) | Meta analysis | Multiple Countries | Meta analysis | Stroke | Herbal medicine | Depression |
| Zhang (2021) | Systematic review with meta-analysis | Multiple Countries | Systematic review with meta-analysis | Stroke | Acupuncture and massage; antidepressants | Depression |
| Zhang (2022) | Meta analysis | Multiple Countries | Meta analysis | Stroke | Antidepressants; acupuncture and massage | Depression |
| Zhang (2023) | Systematic review with meta-analysis | China | Systematic review with meta-analysis | Stroke | Psychotherapy (out-patient) | Quality of life; depression; anxiety |
| Zhang (2023) | Systematic review with meta-analysis | China | Systematic review with meta-analysis | Stroke | Herbal medicine; antidepressants | Depression |
| Zhang (2024) | Systematic review with meta-analysis | China | Systematic review with meta-analysis | Stroke | Acupuncture and massage; music and art | Depression |
| Zhang (2024) | Impact evaluation | China | RCTs/experimental | Stroke | Acute in-patient services; integrated care and case management | Quality of life; cognition |
| Zhangs-h (2021) | Impact evaluation | China | RCTs/experimental | Stroke | Acupuncture and massage | Depression; anxiety |
| Zhao (2018) | Published study protocol | China | RCTs/experimental | Stroke | Herbal medicine | Depression |
| Zhao (2018) | Clinical practice guideline | China | Clinical practice guideline | Stroke | Antidepressants; psychotherapy (out-patient) | Depression |
| Zhao (2019) | Impact evaluation | China | RCTs/experimental | Stroke | Acupuncture and massage | Depression |
| Zhao (2022) | Impact evaluation | China | RCTs/experimental | Stroke | Exercise and movement-based; music and art | Depression |
| Zhen (2022) | Systematic review with meta-analysis | Multiple Countries | Systematic review with meta-analysis | Stroke | Herbal medicine | Depression |
| Zheng (2023) | Systematic review with meta-analysis | China | Systematic review with meta-analysis | Stroke | Tele-health and technology-based | Depression |
| Zhi (2021) | Impact evaluation | China | RCTs/experimental | Stroke | Acupuncture and massage | Depression |
| Zhong (2023) | Systematic review with meta-analysis | China | Systematic review with meta-analysis | Stroke | Acupuncture and massage; psychotherapy (out-patient) | Depression |
| Zhong (2024) | Systematic review with meta-analysis | China | Systematic review with meta-analysis | Stroke | Music and art | Depression |
| Zhou (2020) | Meta analysis | Multiple Countries | Meta analysis | Stroke | Antidepressants | Depression |
| Zhou (2022) | Published study protocol | China | RCTs/experimental | Stroke | Acute in-patient services | Anxiety |
| Zhu (2022) | Impact evaluation | China | RCTs/experimental | Stroke | Neuromodulation and neurofeedback; antidepressants | Depression |
| Zhu (2022) | Impact evaluation | China | RCTs/experimental | Stroke | Non-psychotropic medications | Depression |
| Zhu (2023) | Impact evaluation | China | RCTs/experimental | Stroke | Herbal medicine | Cognition; sleep and eating |
| Zou (2018) | Systematic review with meta-analysis | Multiple Countries | Systematic review with meta-analysis | Stroke | Exercise and movement-based | Depression; activities of daily living/life skills; physical activity |

***2.5***	**Supplementary Table 5.** *AMSTAR-2 Ratings for Included Systematic Reviews.*

| **First Author (Year)** | **Systematic Review Title** | **AMSTAR-2 Rating** | | | |
| --- | --- | --- | --- | --- | --- |
|  |  | **Critically Low** | **Low** | **Moderate** | **High** |
| Acabchuk (2021) | Therapeutic effects of meditation, yoga, and mindfulness-based interventions for chronic symptoms of mild traumatic brain injury: a systematic review and meta-analysis. |  | ✔️ |  |  |
| Ackland (2019) | Effectiveness and harms of mental health treatments in service members and veterans with deployment-related mild traumatic brain injury. |  | ✔️ |  |  |
| Ahrens (2022) | Cognitive-behavioral therapy for managing depressive and anxiety symptoms after stroke: a systematic review and meta-analysis. | ✔️ |  |  |  |
| Ahrens (2023) | Cognitive -behavioral therapy for managing depressive and anxiety symptoms after stroke: a systematic review and meta-analysis. | ✔️ |  |  |  |
| Allida (2019) | Pharmaceutical interventions for emotionalism after stroke. |  |  |  | ✔️ |
| Allida (2020) | Pharmacological, psychological and non-invasive brain stimulation interventions for preventing depression after stroke. |  |  |  | ✔️ |
| Allida (2020) | Pharmacological, psychological, and non-invasive brain stimulation interventions for treating depression after stroke |  |  |  | ✔️ |
| Allida (2023) | Pharmacological, non-invasive brain stimulation and psychological interventions, and their combination, for treating depression after stroke |  |  |  | ✔️ |
| Baker (2018) | A systematic review of rehabilitation interventions to prevent and treat depression in post-stroke aphasia. |  | ✔️ |  |  |
| Balram (2024) | Non-pharmacological interventions to treat mood disturbances post-stroke: a systematic review. | ✔️ |  |  |  |
| Bang-jiang (2018) | Assessment of meta-analysis: systematic review on acupuncture therapy for post stroke depression (psd) | ✔️ |  |  |  |
| Baylan (2016) | The effects of music listening interventions on cognition and mood post-stroke: a systematic review. |  | ✔️ |  |  |
| Beedham (2020) | The management of depression following traumatic brain injury: a systematic review with meta-analysis. |  |  | ✔️ |  |
| Blazquez-gonzalez (2024) | Impact of virtual reality-based therapy on post-stroke depression: a systematic review and meta-analysis of randomized controlled trials. |  | ✔️ |  |  |
| Bucur (2018) | A systematic review of noninvasive brain stimulation for post-stroke depression. |  | ✔️ |  |  |
| Byrne (2016) | The effectiveness of psychological interventions for aggressive behavior following acquired brain injury: a meta-analysis and systematic review. | ✔️ |  |  |  |
| Cai (2021) | Efficacy and safety of modified sini san for treating poststroke depression: a meta-analysis of randomized controlled trials. | ✔️ |  |  |  |
| Chan (2022) | A systematic review on integrated care for traumatic brain injury, mental health, and substance use. |  | ✔️ |  |  |
| Chan (2023) | Effects of visual arts-based interventions on physical and psychosocial outcomes of people with stroke: a systematic review and meta-analysis. |  | ✔️ |  |  |
| Chen (2020) | Development of a care bundle for stroke survivors with psychological symptoms: evidence summary and delphi study. |  |  | ✔️ |  |
| Chen (2020) | Effects of cognitive behavioral therapy for adults with post-concussion syndrome: a systematic review and meta-analysis of randomized controlled trials. |  | ✔️ |  |  |
| Chen (2024) | Effects of home-based exercise interventions on post-stroke depression: a systematic review and network meta-analysis. |  | ✔️ |  |  |
| Chen (2024) | Efficacy and safety of agomelatine versus ssris/snris for post-stroke depression: a systematic review and meta-analysis of randomized controlled trials. | ✔️ |  |  |  |
| Cheng (2021) | Therapeutic benefits of pharmacologic and nonpharmacologic treatments for depressive symptoms after traumatic brain injury: a systematic review and network meta-analysis. |  |  | ✔️ |  |
| Choo (2022) | Effectiveness of caregiver-mediated exercise interventions on activities of daily living, anxiety and depression post-stroke rehabilitation: a systematic review and meta-analysis. |  |  |  | ✔️ |
| Chu (2023) | Systematic review: pharmacological interventions for the treatment of post-stroke fatigue |  |  | ✔️ |  |
| Chun (2018) | A systematic review of anxiety interventions in stroke and acquired brain injury: efficacy and trial design. |  | ✔️ |  |  |
| Clay (2019) | Prophylaxis pharmacotherapy to prevent the onset of post-traumatic brain injury depression: a systematic review. |  |  | ✔️ |  |
| Cui (2018) | Efficacy and safety of citalopram for the treatment of poststroke depression: a meta-analysis. | ✔️ |  |  |  |
| D'anci (2019) | Treatments for poststroke motor deficits and mood disorders: a systematic review for the (2019) u.s. department of veterans affairs and u.s. department of defense guidelines for stroke rehabilitation. | ✔️ |  |  |  |
| Dayuan (2022) | The effect of music as an intervention for post-stroke depression: a systematic review and meta-analysis. |  | ✔️ |  |  |
| Deng (2017) | Interventions for management of post-stroke depression: a bayesian network meta-analysis of 23 randomized controlled trials. | ✔️ |  |  |  |
| Deng (2018) | Efficacy and tolerability of pharmacotherapy for post-stroke depression: a network meta-analysis. |  | ✔️ |  |  |
| Dolbow (2020) | The effects of physical exercise on depression for individuals with traumatic brain injury: a systematic review | ✔️ |  |  |  |
| Eng (2014) | Exercise for depressive symptoms in stroke patients: a systematic review and meta-analysis. |  | ✔️ |  |  |
| Feng (2022) | Efficacy of escitalopram for poststroke depression: a systematic review and meta-analysis. | ✔️ |  |  |  |
| Galimberti (2024) | Effectiveness of rtms and tdcs treatment for chronic tbi symptoms: a systematic review and meta-analysis. | ✔️ |  |  |  |
| Gao (2019) | The influence of sertraline on depressive disorder after traumatic brain injury: a meta-analysis of randomized controlled studies. | ✔️ |  |  |  |
| Gertler (2015) | Non-pharmacological interventions for depression in traumatic brain injury |  |  |  | ✔️ |
| Guo (2022) | Moxibustion for treating patients with post-stroke depression: a systematic review and meta-analysis. |  | ✔️ |  |  |
| Han (2023) | Mindfulness- and acceptance-based interventions for stroke survivors: a systematic review and meta-analysis |  |  | ✔️ |  |
| Hao (2023) | Transcranial direct current stimulation for the treatment of post-stroke depression: a systematic review |  |  |  | ✔️ |
| Hicks (2020) | Pharmacotherapy for the pseudobulbar affect in individuals who have sustained a traumatic brain injury: a systematic review. |  | ✔️ |  |  |
| Hicks (2021) | Efficacy and harms of pharmacological interventions for anxiety after traumatic brain injury: systematic review. |  | ✔️ |  |  |
| Hung (2019) | Overview of systematic reviews with meta-analyses on acupuncture in post-stroke cognitive impairment and depression management. |  | ✔️ |  |  |
| Hwang (2021) | Telehealth interventions to support self-management in stroke survivors: a systematic review. |  | ✔️ |  |  |
| Jennings (2023) | Examining the interdisciplinary approach for treatment of persistent post-concussion symptoms in adults: a systematic review. |  | ✔️ |  |  |
| Jiang (2023) | Efficacy and safety of scalp acupuncture for poststroke depression: a meta-analysis and systematic review. |  | ✔️ |  |  |
| Kalbouneh (2022) | Safety and efficacy of ssris in improving poststroke recovery: a systematic review and meta-analysis. | ✔️ |  |  |  |
| Knapp (2017) | Interventions for treating anxiety after stroke. |  |  | ✔️ |  |
| Komber (2024) | Non-pharmacological interventions for the treatment of post-stroke fatigue: a systematic review |  |  |  | ✔️ |
| Kwon (2019) | Efficacy and safety of sihogayonggolmoryeo-tang (saikokaryukotsuboreito, chai-hu-jia-long-gu-mu-li-tang) for post-stroke depression: a systematic review and meta-analysis. |  |  |  | ✔️ |
| Kwon (2019) | Herbal medicine for post-stroke anxiety: a systematic review and meta-analysis of randomized controlled trials. | ✔️ |  |  |  |
| Lamching (2023) | Acupuncture for post-stroke depression: a systematic review and network meta-analysis. |  | ✔️ |  |  |
| Lavu (2022) | Evaluation and treatment of depression in stroke patients: a systematic review. | ✔️ |  |  |  |
| Le (2024) | Effectiveness of problem-solving therapy in improving patient mental health, function, quality of life, and mortality post-stroke: a systematic review. | ✔️ |  |  |  |
| Lee (2019) | Content and effectiveness of interventions focusing on community participation poststroke: a systematic review. | ✔️ |  |  |  |
| Lee (2021) | Effectiveness of non-pharmacological interventions for treating post-stroke depressive symptoms: systematic review and meta-analysis of randomized controlled trials. |  |  |  | ✔️ |
| Li (2018) | Clinical effects and safety of electroacupuncture for the treatment of post-stroke depression: a systematic review and meta-analysis of randomised controlled trials. |  | ✔️ |  |  |
| Li (2020) | Comparative efficacy of nine antidepressants in treating chinese patients with post-stroke depression: a network meta-analysis. |  | ✔️ |  |  |
| Li (2022) | Effects of transcranial direct current stimulation for post-stroke depression: a systematic review and meta-analysis. |  | ✔️ |  |  |
| Liang 202 | Hyperbaric oxygen therapy for post-stroke depression: a systematic review  and meta-analysis |  | ✔️ |  |  |
| Liang (2022) | Effects of noninvasive brain stimulation combined with antidepressants in patients with poststroke depression: a systematic review and meta-analysis. | ✔️ |  |  |  |
| Little (2021) | The effectiveness of cognitive behaviour therapy for reducing anxiety symptoms following traumatic brain injury: a meta-analysis and systematic review. |  |  | ✔️ |  |
| Liu (2018) | Neuropsychological rehabilitation and psychotherapy of adult traumatic brain injury patients with depression: a systematic review and meta-analysis. | ✔️ |  |  |  |
| Liu (2019) | Efficacy and safety of high-frequency repetitive transcranial magnetic stimulation for poststroke depression: a systematic review and meta-analysis. |  | ✔️ |  |  |
| Liu (2021) | Acupuncture for post-stroke depression: a systematic review and meta-analysis. |  | ✔️ |  |  |
| Liu (2023) | Effects of virtual reality-based intervention on depression in stroke patients: a meta-analysis. |  | ✔️ |  |  |
| Love (2019) | Mind-body interventions, psychological stressors, and quality of life in stroke survivors: a systematic review | ✔️ |  |  |  |
| Maksimowski (2016) | Efficacy of stimulants for psychiatric symptoms in individuals with traumatic brain injury. |  | ✔️ |  |  |
| Mi (2024) | Effective evaluations of community nursing on rehabilitation for stroke survivors: a meta-analysis. |  | ✔️ |  |  |
| Mikolic (2019) | Treatment for posttraumatic stress disorder in patients with a history of traumatic brain injury: a systematic review. | ✔️ |  |  |  |
| Mou (2021) | Effectiveness of dyadic psychoeducational intervention for stroke survivors and family caregivers on functional and psychosocial health: a systematic review and meta-analysis |  |  | ✔️ |  |
| Narapareddy (2020) | Treatment of depression after traumatic brain injury: a systematic review focused on pharmacological and neuromodulatory interventions. | ✔️ |  |  |  |
| Oh (2022) | The effectiveness of self-management interventions with action-taking components in improving health-related outcomes for adult stroke survivors: a systematic review and meta-analysis. |  | ✔️ |  |  |
| Pan (2023) | Effects of low-frequency rtms combined with antidepressants on depression in patients with post-stroke depression: a systematic review and meta-analysis. |  | ✔️ |  |  |
| Paraschakis (2017) | Antidepressants for depression associated with traumatic brain injury: a meta-analytical study of randomised controlled trials. | ✔️ |  |  |  |
| Peng (2014) | Effectiveness and safety of wuling capsule for post stroke depression: a systematic review. | ✔️ |  |  |  |
| Peppel (2020) | Pharmacological and non-pharmacological interventions for depression after moderate-to-severe traumatic brain injury: a systematic review and meta-analysis. | ✔️ |  |  |  |
| Perry (2020) | The effectiveness of physical exercise as an intervention to reduce depressive symptoms following traumatic brain injury: a meta-analysis and systematic review. |  |  | ✔️ |  |
| Plantier (2016) | Drugs for behavior disorders after traumatic brain injury: systematic review and expert consensus leading to french recommendations for good practice. | ✔️ |  |  |  |
| Qin (2021) | Environmental enrichment for stroke and other non-progressive brain injury |  |  | ✔️ |  |
| Reyes (2019) | Efficacy of sertraline in post-traumatic brain injury (post-tbi) depression and quality of life: a systematic review and meta-analysis of randomized controlled trials. |  | ✔️ |  |  |
| Richter (2021) | Selective serotonin reuptake inhibitors for the prevention of post-stroke depression: a systematic review and meta-analysis | ✔️ |  |  |  |
| Ryan (2022) | A systematic review of non-drug interventions to prevent and treat anxiety in people with aphasia after stroke. |  |  | ✔️ |  |
| Salter (2016) | Pharmacotherapy for depression posttraumatic brain injury: a meta-analysis. | ✔️ |  |  |  |
| Shao (2020) | Effectiveness and safety of the xuefu zhuyu tang for post-stroke depression: a systematic review and meta-analysis |  | ✔️ |  |  |
| Shao (2021) | Efficacy of repetitive transcranial magnetic stimulation for post-stroke depression: a systematic review and meta-analysis of randomized clinical trials. |  |  |  | ✔️ |
| Shek (2021) | Technology-based interventions for mental health support after stroke: a systematic review of their acceptability and feasibility. | ✔️ |  |  |  |
| Shen (2017) | Repetitive transcranial magnetic stimulation for the treatment of post-stroke depression: a systematic review and meta-analysis of randomized controlled clinical trials. |  | ✔️ |  |  |
| Shen (2022) | Repetitive transcranial magnetic stimulation and transcranial direct current stimulation as treatment of poststroke depression: a systematic review and meta-analysis. | ✔️ |  |  |  |
| Song (2023) | Effects of multicomponent exercise on quality of life, depression and anxiety among stroke survivors: a systematic review and meta-analysis. |  | ✔️ |  |  |
| Srisurapanont (2021) | Blue-wavelength light therapy for post-traumatic brain injury sleepiness, sleep disturbance, depression, and fatigue: a systematic review and network meta-analysis. |  | ✔️ |  |  |
| Storm (2019) | The effectiveness of mental practice interventions on psychological health in stroke patients: a systematic review | ✔️ |  |  |  |
| Su (2021) | A meta-analysis of the effect of abdominal acupuncture on post-stroke depression. |  | ✔️ |  |  |
| Su (2024) | Chinese herbal medicine and acupuncture for insomnia in stroke patients: a systematic review and meta-analysis of randomised controlled trials |  |  |  | ✔️ |
| Su (2024) | Effects of mind-body exercise on physical and psychosocial well-being of stroke patients: a systematic review and network meta-analysis. |  | ✔️ |  |  |
| Suarilah (2024) | Effectiveness of telehealth interventions among traumatic brain injury survivors: a systematic review and meta-analysis. | ✔️ |  |  |  |
| Sun (2017) | Comparative efficacy and acceptability of antidepressant treatment in poststroke depression: a multiple-treatments meta-analysis. |  | ✔️ |  |  |
| Tao (2022) | Effectiveness of mindfulness-based stress reduction and mindfulness-based cognitive therapy on depression in poststroke patients-a systematic review and meta-analysis of randomized controlled trials. |  | ✔️ |  |  |
| Termors (2019) | Efficacy of amantadine on behavioural problems due to acquired brain injury: a systematic review. |  | ✔️ |  |  |
| Towfighi (2017) | Poststroke depression: a scientific statement for healthcare professionals from the american heart association/american stroke association. | ✔️ |  |  |  |
| Tracey (2023) | Effectiveness of interventions for the improvement of mental health and well-being post-concussion: a systematic review. | ✔️ |  |  |  |
| Tsai (2021) | Effect of repetitive transcranial magnetic stimulation on depression and cognition in individuals with traumatic brain injury: a systematic review and meta-analysis. | ✔️ |  |  |  |
| Vallury (2015) | Do family-oriented interventions reduce poststroke depression? a systematic review and recommendations for practice. |  |  |  | ✔️ |
| Verberne (2019) | Psychological interventions for treating neuropsychiatric consequences of acquired brain injury: a systematic review. | ✔️ |  |  |  |
| Wan (2021) | Effects of peer support interventions on physical and psychosocial outcomes among stroke survivors: a systematic review and meta-analysis. | ✔️ |  |  |  |
| Wan (2021) | Efficacy and safety of chaihu jia longgu muli decoction in the treatment of poststroke depression: a systematic review and meta-analysis. |  |  |  | ✔️ |
| Wan (2024) | Cognitive behavioural therapy for depression, quality of life, and cognitive function in the post-stroke period: systematic review and meta-analysis. | ✔️ |  |  |  |
| Wang (2018) | Cognitive behavioral therapy for post-stroke depression: a meta-analysis. |  | ✔️ |  |  |
| Wang (2021) | Is electroacupuncture an effective and safe treatment for poststroke depression? an updated systematic review and meta-analysis. | ✔️ |  |  |  |
| Wang (2021) | Meta-analysis of the clinical effectiveness of combined acupuncture and western medicine to treat post-stroke depression. | ✔️ |  |  |  |
| Wang (2022) | Efficacy and safety of xiaoyao recipe in the treatment of poststroke depression: a systematic review and meta-analysis. |  | ✔️ |  |  |
| Wiart (2016) | Non pharmacological treatments for psychological and behavioural disorders following traumatic brain injury (tbi). a systematic literature review and expert opinion leading to recommendations. | ✔️ |  |  |  |
| Williamson (2019) | Pharmacological interventions for agitated behaviours in patients with traumatic brain injury: a systematic review. |  | ✔️ |  |  |
| Woranush (2021) | Preventive approaches for post-stroke depression: where do we stand? a systematic review. |  | ✔️ |  |  |
| Xiao (2024) | Acupuncture combined with repetitive transcranial magnetic stimulation for the treatment of post-stroke depression: a systematic evaluation and meta-analysis based on a randomised controlled trial. |  | ✔️ |  |  |
| Xu (2016) | Efficacy and feasibility of antidepressant treatment in patients with post-stroke depression. | ✔️ |  |  |  |
| Xu (2019) | Clinical efficacy and safety of xiaoyao pill in post-stroke depression: a systematic review and meta-analysis of randomized controlled trials | ✔️ |  |  |  |
| Yi (2024) | Effectiveness of non-pharmacological therapies for treating post-stroke depression: a systematic review and network meta-analysis. |  | ✔️ |  |  |
| Yisma (2024) | Effect of behavioural activation for individuals with post-stroke depression: systematic review and meta-analysis. | ✔️ |  |  |  |
| Zeng (2016) | Is adjunctive treatment with medication of liver-soothing-oriented method beneficial for depression after cerebrovascular accident?: a prisma-compliant meta-analysis. | ✔️ |  |  |  |
| Zeng (2017) | Role of medicinal plants for liver-qi regulation adjuvant therapy in post-stroke depression: a systematic review of literature. |  | ✔️ |  |  |
| Zhang (2014) | System review on treating post-stroke depression with acupuncture | ✔️ |  |  |  |
| Zhang (2019) | The effectiveness of acupuncture therapy in patients with post-stroke depression: an updated meta-analysis of randomized controlled trials. | ✔️ |  |  |  |
| Zhang (2021) | Comparison between acupuncture and antidepressant therapy for the treatment of poststroke depression: systematic review and meta-analysis. | ✔️ |  |  |  |
| Zhang (2021) | Does acupuncture combined with antidepressants have a better therapeutic effect on post-stroke depression? a systematic review and meta-analysis. |  |  | ✔️ |  |
| Zhang (2021) | Impact of virtual reality-based therapies on cognition and mental health of stroke patients: systematic review and meta-analysis |  | ✔️ |  |  |
| Zhang (2023) | Dyadic psycho-social interventions for stroke survivors and family caregivers: a systematic review and meta-analysis of randomized controlled trials. |  | ✔️ |  |  |
| Zhang (2023) | Herbal medicine as adjunctive therapy with antidepressants for post-stroke depression: a systematic review and network meta-analysis of randomized controlled trials. | ✔️ |  |  |  |
| Zhang (2024) | Effectiveness of acupuncture plus music therapy for post-stroke depression: systematic review and meta-analysis. | ✔️ |  |  |  |
| Zhen (2022) | Efficacy and safety of buyang huanwu decoction in the treatment of post-stroke depression: a systematic review and meta-analysis of 15 randomized controlled trials. |  | ✔️ |  |  |
| Zheng (2023) | Effectiveness of telehealth interventions on depression symptoms after stroke: a systematic review and meta-analysis. | ✔️ |  |  |  |
| Zhong (2023) | Efficacy of scalp acupuncture combined with conventional therapy in the intervention of post-stroke depression: a systematic review and meta-analysis. |  | ✔️ |  |  |
| Zhong (2024) | The effectiveness of five-element music therapy for post-stroke depression: a systematic review and meta-analysis. |  | ✔️ |  |  |
| Zou (2018) | Effects of mind-body exercises for mood and functional capabilities in patients with stroke: an analytical review of randomized controlled trials. | ✔️ |  |  |  |

*Note:* Included systematic reviews are ordered alphabetically by first author last name.
